# Syntheses and crystal structures of [Ir^III^{C(CHCO_2_Et)(dppm)_2_-κ^4^ *P*,*C*,*C*′,*P*′}ClH]Cl·2.75CH_2_Cl_2_ and its derivatives, [Ir^III^{C(CHCO_2_Et)(dppm)_2_-κ^4^ *P*,*C*,*C*′,*P*′}(CH_2_CO_2_Et)Cl]Cl·CH_3_OH·0.5H_2_O, [Ir^III^{C(CHCO_2_Et)(dppm)_2_-κ^4^ *P*,*C*,*C*′,*P*′}Cl_2_]Cl·CH_3_OH·2H_2_O and [Ir^III^{C(CHCO_2_Et)(dppm)_2_-κ^4^ *P*,*C*,*C*′,*P*′}(CH_2_CO_2_Et)(CO)]Cl_2_·2CH_2_Cl_2_·1.5H_2_O

**DOI:** 10.1107/S2056989018017024

**Published:** 2019-01-01

**Authors:** Inge Schlapp-Hackl, Christoph Falschlunger, Kathrin Zauner, Walter Schuh, Holger Kopacka, Klaus Wurst, Paul Peringer

**Affiliations:** a University of Innsbruck, Faculty of Chemistry and Pharmacy, Innrain 80-82, 6020 Innsbruck, Austria

**Keywords:** carbodi­phospho­rane (CDP), PCP pincer, diazo compounds, cyclo­addition, iridium(III), C–C coupling reaction, non-innocent behaviour, alkyl­idene bridge, carbene inter­mediate, insertion reaction, crystal structure

## Abstract

The common feature of the four iridium(III) salt complexes, (bis­{[(di­phenyl­phosphan­yl)meth­yl]di­phenyl­phosphanyl­idene}(eth­oxy­oxoethanyl­idene)methane-κ^4^ *P*,*C*,*C*′,*P*′)chlorido­hydridoiridium(III) chloride methyl­ene chloride 2.75-solvate (**4**), (bis­{[(di­phenyl­phosphan­yl)meth­yl]di­phenyl­phosphanyl­idene}(eth­oxy­oxoethanyl­idene)methane-κ^4^ *P*,*C*,*C*′,*P*′)chlorido­(eth­oxy­oxoethanido)iridium(III) chloride–methanol–water (1/1/0.5) (**5**), (bis­{[(di­phenyl­phosphan­yl)meth­yl]di­phenyl­phosphanyl­idene}(eth­oxy­oxoethanyl­idene)methane-κ^4^ *P*,*C*,*C*′,*P*′)di­chlorido­iridium(III) chloride–methanol–water (1/1/2) (**6**) and (bis­{[(di­phenyl­phosphan­yl)meth­yl]di­phenyl­phosphanyl­idene}(eth­oxy­oxoethanyl­idene)methane-κ^4^ *P*,*C*,*C*′,*P*′)carbon­yl(eth­oxy­oxoethanide)iridium(III) dichloride–meth­yl­ene chloride–water (1/2/1.5) **(7)** or in terms of their formulae [Ir(C_55_H_50_O_2_P_4_)ClH]Cl·2.75CH_2_Cl_2_ (**4**), [Ir(C_4_H_7_O_2_)(C_55_H_50_O_2_P_4_)Cl]Cl·CH_3_OH·0.5H_2_O (**5**), [Ir(C_55_H_50_O_2_P_4_)Cl_2_]Cl·CH_3_OH·2H_2_O (**6**) and [Ir(C_4_H_7_O_2_)(C_55_H_50_O_2_P_4_)(CO)]Cl_2_·2CH_2_Cl_2_·1.5H_2_O (**7**) is a central Ir^III^ atom coordin­ated in a distorted octa­hedral fashion by a PCCP ligand system and two additional residues, such as chlorides, a hydride, a carbonyl or an alkyl unit. Thereby, the PCP pincer ligand system and the residue *trans* to the carbodi­phospho­rane (CDP) C atom surround the iridium(III) transition metal in the equatorial plane under the formation of two five-membered dissimilar chelate rings [C—C_CDP_—P (**4**, **5**, **6** and **7**) for the first ring: 120.2 (3), 121.9 (5), 111.2 (3) and 121.7 (2) °; for the second ring: 112.1 (3), 113.5 (5), 120.5 (3) and 108.3 (2)°]. A cyclo­propane-like heterocycle is positioned approximately orthogonal (84.21–88.85°) to the equatorial plane, including an alkyl­idene bridge connecting the Ir^III^ atom and the coordinating CDP atom of the PCP subunit. In general, the neutral PCCP ligand system coordinates the metal in a tetra­dentate way via three Lewis acid/base bonds and by an alkyl­idene unit presenting strengthened inter­actions. In all the crystal structures, (disordered) solvent mol­ecules are present in the voids of the packed mol­ecules that inter­act with the positively charged complex and its chloride counter-ion(s) through weak hydrogen bonding.

## Abstract 2

The common structural feature of the four title Ir^III^ compounds is the octa­hedral coordination of the Ir^III^ atom by a PCP pincer complex, a C atom of a (eth­oxy­oxoethanyl­idene)methane group and two variable ligands *X* (H, CH_2_CO_2_Et, Cl) and *Y* (Cl, CO).

## Chemical context   

Carbodi­phospho­ranes (CDP) in combination with transition metals initialize a huge variety of functionalities. As a result of the presence of two *σ-*electron-donor groups, preferred in the form of tertiary phosphines, the stabilization of two free-electron pairs with *σ*- and simultaneously *π*-symmetry, the establishment of a localized electron octet and further the creation of a zero-valent, naked carbon atom in an excited singlet (^1^ *D*) state is possible (Petz & Frenking, 2010 ▸). The carbodi­phospho­rane C atom can be considered as a four-electron donor, and accordingly enables the coordination of two Lewis acids, such as protons or different metal cations. Our inter­ests focus on the combination of a carbodi­phospho­rane pincer ligand system, [CH(dppm)_2_]Cl (dppm = 1,1-bis­(di­phenyl­phosphino)methane; Reitsamer *et al.*, 2012 ▸), with reactive functionalities to enter new reaction pathways, to create new complexes and to analyse in detail the new properties obtained. In general, C—C coupling reactions can be induced via the use of diazo compounds such as ethyl diazo­acetate. As a result of the presence of two nitro­gen atoms acting together as an excellent leaving group, and an alkyl­idene group stabilized by different functionalities, the electrons are delocalized between three atoms and thus a positive and one negative charge theoretically allows by a disregard of the coordinating residuals and chemical conditions four different resonance structures to be gained in total. Therefore, the diazo compound can be regarded as both a nucleophilic and as an electrophilic reaction partner. By the use of this compound, a targeted synthesis of cyclo­propanes or rather hetero­cyclo­propanes, consisting of a transition metal, an electron-donor atom and a carbene carbon, is possible and has been reported several times in the literature (*e.g*. Nomura *et al.*, 2011 ▸; Liu & Yan, 2015 ▸; Malisch *et al.*, 1998 ▸; Strecker *et al.*, 1991 ▸; Zhang *et al.*, 2005 ▸, and references cited therein). An electrophilic reaction partner such as a transition metal establishes a nucleophilic attack of the diazo subunit and, according to the choice of the reaction conditions, the elimination of the nitro­gen leaving group is supported. Consequently, the alkyl­idene carbon atom is stabilized by coordination of an electron-accepting atom and a reactive carbene inter­mediate complex is formed. The existence of a nucleophilic reaction partner in the vicinity of the carbene atom results in the formation of a ring including an alkyl­idene bridging subunit. In summary, the reaction of a diazo compound with an electrophilic and additionally a nucleophilic reaction partner initiates a mechanism that can be described as a cheletropic-like process. Inspired by this reaction sequence, we have synthesized a three-membered heterocycle by the combination of an ethyl diazo­acetate and an iridium(III) PCP pincer carbodi­phospho­rane complex.

**Figure 1:**
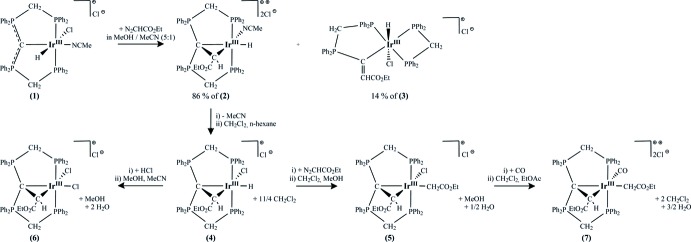
Scheme (Cambridge Soft, 2001 ▸) for the synthesis and crystallization of the title compounds **4**–**7**.

If the starting materials [CH(dppm)_2_]Cl (Reitsamer *et al.*, 2012 ▸) and [IrCl(cod)]_2_ are mixed, a reaction sequence is initialized that consists of the following steps: Coordination of the iridium(I) atom, followed by deprotonation of the carbodi­phospho­rane carbon atom, the generation of a hydrido ligand caused by an oxidation of the iridium(I) atom and the formation of the [Ir{C(dppm)_2_-*κ^3^P,C,P‘*}ClH(MeCN)]Cl complex **1** (Schlapp-Hackl *et al.*, 2018 ▸; Fig. 1 ▸). In summary, the iridium(III) transition metal is stabilized by the PCP pincer ligand system, and by a chlorido and a hydrido ligand and an aceto­nitrile solvent mol­ecule. The addition of ethyl diazo­acetate causes, via loss of the di­nitro­gen subunit, the formation of an Ir^III^–carbene bond. As a result of the presence of the second free lone-electron pair at the carbodi­phospho­rane carbon atom, a cyclization and further the creation of an alkyl­idene bridge is accomplished. The formation of the three-membered Ir—C_CDP_—C ring is accompanied by a surprising displacement of the hydrido ligand from a position perpendicular to the plane of the PCP pincer system to a meridional arrangement *trans* to the carbodi­phospho­rane carbon atom. Supported by the polar solvent mixture methanol/aceto­nitrile (*v*/*v* 5:1) an [Ir^III^{C(CHCO_2_Et)(dppm)_2_-κ^4^ *P*,*C*,*C*′,*P*′}H(MeCN)]Cl_2_ precursor system (**2**) is generated in high yields (86%). Moreover, the preparation of complex **2** in a less polar solvent environment like chloro­form/aceto­nitrile or in a solvent mixture of methyl­ene chloride/aceto­nitrile (*v*/*v* 5:1) is not possible and qu­anti­tatively results in the substitution of one phosphine moiety of the carbodi­phospho­rane functionality against the carbene CHCO_2_Et subunit. An [Ir{C(CHCO_2_Et)(dppm)-*κ^2^P,C*}Cl(dppm)H]Cl complex **3** is generated, offering a phospho­rus ylide carbon backbone (Schlapp-Hackl *et al.*, 2018 ▸). To a lesser extent (14% yield), this complex is additionally obtained as by-product by the production of complex **2**. Heating of complex **2** in methanol/aceto­nitrile (*v*/*v* 5:1) to 333 K for 2 h benefits the ring-opening reaction of the PCCP pincer ligand system. Therefore, a reorganization of the ligand system is supported, resulting in the qu­anti­tative formation of complex **3**. Furthermore, evaporation of the reaction mixture of complex **2** causes an exchange of the aceto­nitrile solvent ligand with a chloride counter-ion and the creation of the desired [Ir^III^{C(CHCO_2_Et)(dppm)_2_-κ^4^ *P*,*C*,*C*′,*P*′}ClH]Cl complex **4**. 
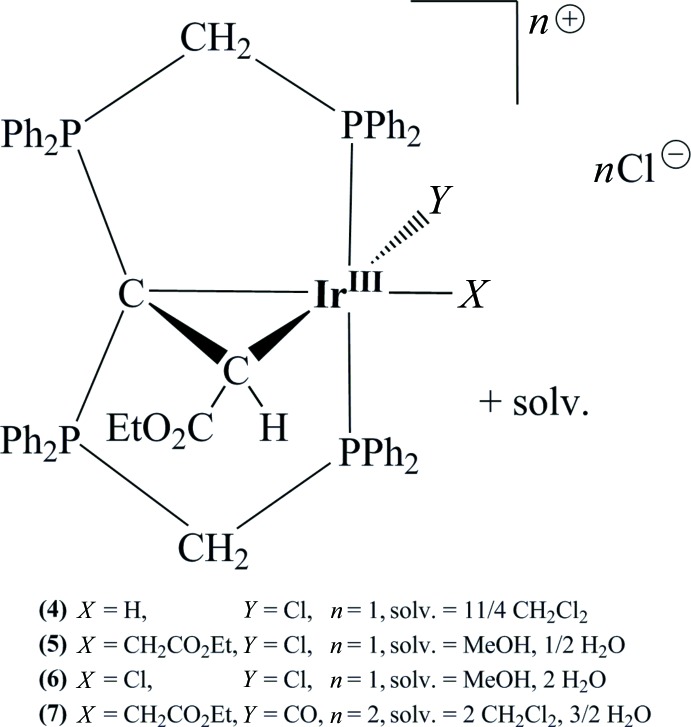


The stucture of this irid­ium(III) PCCP complex was completely determined by NMR spectroscopy and X-ray crystallography, but up to now crystallization attempts of the inter­mediates, **1** and **2**, were unsuccessful. With regard to a ruthenium PCP pincer complex, a related cyclo­addition was monitored (Zhang *et al.*, 2005 ▸). Thereby, the ruthenium transition metal first stabilizes the phenyl­diazo­methane by coordination. After the elimination of the di­nitro­gen mol­ecule, the formation of the corresponding carbene complex and finally a carbon–carbon coupling reaction between the central carbon atom of the phenyl-based PCP ligand and the carbene was detected. As a consequence, the arene backbone of the PCP ligand system is transformed to an arenium moiety. Treatment of complex **4** with an additional equivalent amount of ethyl diazo­acetate causes an insertion reaction of the alkyl­idene to the iridium(III)–hydrido bond and the formation of the [Ir^III^{C(CHCO_2_Et)(dppm)_2_-κ^4^ *P*,*C*,*C*′,*P*′}(CH_2_CO_2_Et)Cl]Cl alkyl derivative **5**. This reaction procedure is well known, and the mechanism of the inter­molecular insertion reaction has been clarified via an inter­mediate carbene complex (Cohen *et al.*, 2003 ▸). Moreover, treatment of complexes **4** and **5** with hydro­chloric acid leads to a ligand substitution at the position *trans* to the central carbon atom of the PCP pincer ligand system with a chloride ion and to the formation of the [Ir^III^{C(CHCO_2_Et)(dppm)_2_-κ^4^ *P*,*C*,*C*′,*P*′}Cl_2_]Cl complex **6**. Besides, a replacement of the chlorido ligand of compound **5** by a carbonyl group is possible and results in the [Ir^III^{C(CHCO_2_Et)(dppm)_2_-κ^4^ *P*,*C*,*C*′,*P*′}(CH_2_CO_2_Et)(CO)]Cl_2_ complex **7.**

Here we report details of the syntheses and crystal structures of complexes **4**–**7**.

## Structural commentary   

**Figure 2:**
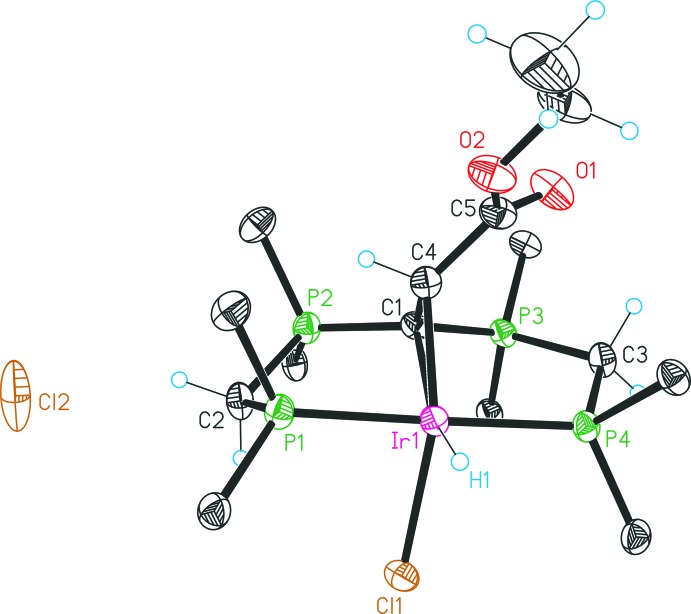
Mol­ecular structure of the complex cation in **4** and the counter-anion. Displacement ellipsoids are drawn at the 30% probability level. For clarity, only the *ipso* carbon atoms of the phenyl groups are presented and the solvent mol­ecules are omitted.

The asymmetric unit of compound **4**, [Ir^III^{C(CHCO_2_Et)(dppm)_2_-κ^4^ *P*,*C*,*C*′,*P*′}ClH]Cl, comprises of one formula unit of **4** and additionally of 2.75 mol­ecules of methyl­ene chloride solvent mol­ecules. The central iridium(III) transition metal is surrounded in a distorted octa­hedral fashion by a PCCP pincer-like ligand system, and anionic chlorido and hydrido ligands (Fig. 2 ▸). The neutral [C(CHCO_2_Et)(dppm)_2_-κ^4^ *P*,*C*,*C*′,*P*′] ligand coordinates the Ir^III^ metal in a tetra­dentate fashion via two P and two C atoms under formation of two five-membered, dissimilar chelate rings [C4—C1—P3 = 120.2 (3)°, C4—C1—P2 = 112.1 (3)°] and one three membered heterocycle. The PCP ligand exhibits a meridional arrangement with the hydrido ligand completing the equatorial plane *trans* to the C1 carbodi­phospho­rane atom. A cyclo­propane-like chelate ring is positioned nearly normal (84.21°) to the equatorial plane, and a chlorido ligand is positioned *trans* to the alkyl­idene carbon atom C4. The Ir—C1 [2.273 (4) Å] and Ir—C4 [2.072 (5) Å] distances differ significantly and consequently these values substanti­ate a strengthened inter­action between the iridium(III) metal and the alkyl­idene carbon atom. The C1—C4 separation [1.515 (6) Å] is slightly shorter in comparison to a typical C—C single bond but, in general, very close to that of cyclo­propanes. However, in comparison with a cyclo­propane mol­ecule the C4—Ir1—C1 [40.5 (2)°], C4—C1—Ir1 [62.6 (2)°] and C1—C4—Ir1 [76.9 (3)°] angles emphasise a significant distortion of the synthesized three-membered heterocycle. All mentioned geometric features of this strained Ir—C1—C4 metallacycle can be associated with the structural results of the Ru—C—C triangle reported by Zhang *et al.* (2005 ▸). Furthermore, the three-membered ring causes a distortion of the octa­hedral coordination geometry (Table 1 ▸). The P1—Ir1—P4 [178.4 (1)°] atoms are less affected and show only a slight deviation from linearity. Though, the tetra­hedral environment of the carbodi­phospho­rane C1 atom is strongly influenced and thus distorted, which is reflected by a P3—C1—P2 angle of 124.2 (3)°. Overall, the transition metal and its ligand system present a cationic complex balanced by one chloride.

**Figure 3:**
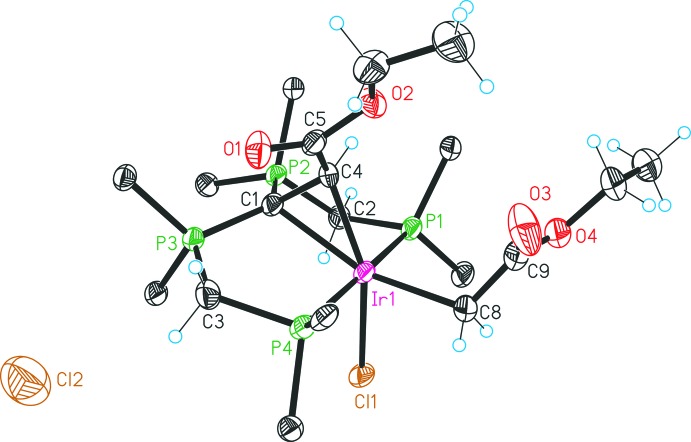
Mol­ecular structure of the complex cation in **5** and the counter-anion. Displacement ellipsoids are drawn at the 30% probability level. For clarity, only the *ipso* carbon atoms of the phenyl groups are presented and the solvent mol­ecules are omitted.

The asymmetric unit of compound **5**, [Ir^III^{C(CHCO_2_Et)(dppm)_2_-κ^4^ *P*,*C*,*C*′,*P*′}(CH_2_CO_2_Et)Cl]Cl, is defined by one complex **5**, one half-occupied water mol­ecule and one disordered methanol solvent mol­ecule. In comparison with the structural features discussed in detail for compound **4**, significant differences pertain only to the equatorial position *trans* to C1. Here the hydrido ligand in **4** is exchanged by an ethyl acetate unit (Fig. 3 ▸).

**Figure 4:**
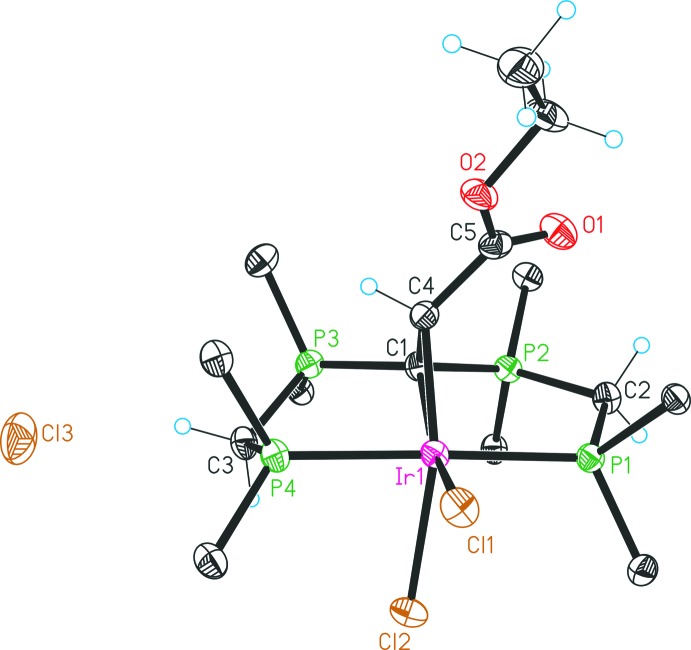
Mol­ecular structure of the complex cation in **6** and the counter-anion. Displacement ellipsoids are drawn at the 30% probability level. For clarity, only the *ipso* carbon atoms of the phenyl groups are presented and the solvent mol­ecules are omitted.

The replacement of the hydrido ligand of compound **4** by a chlorido ligand led to formation of **6**, [Ir^III^{C(CHCO_2_Et)(dppm)_2_-κ^4^ *P*,*C*,*C*′,*P*′}Cl_2_]Cl. In its crystalline form, besides one formula unit of **6**, one solvent mol­ecule of MeOH and two water mol­ecules in total are present in the asymmetric unit. Overall, this PCCP derivative shows very similar structural characteristics (Fig. 4 ▸) to complex **4**.

**Figure 5:**
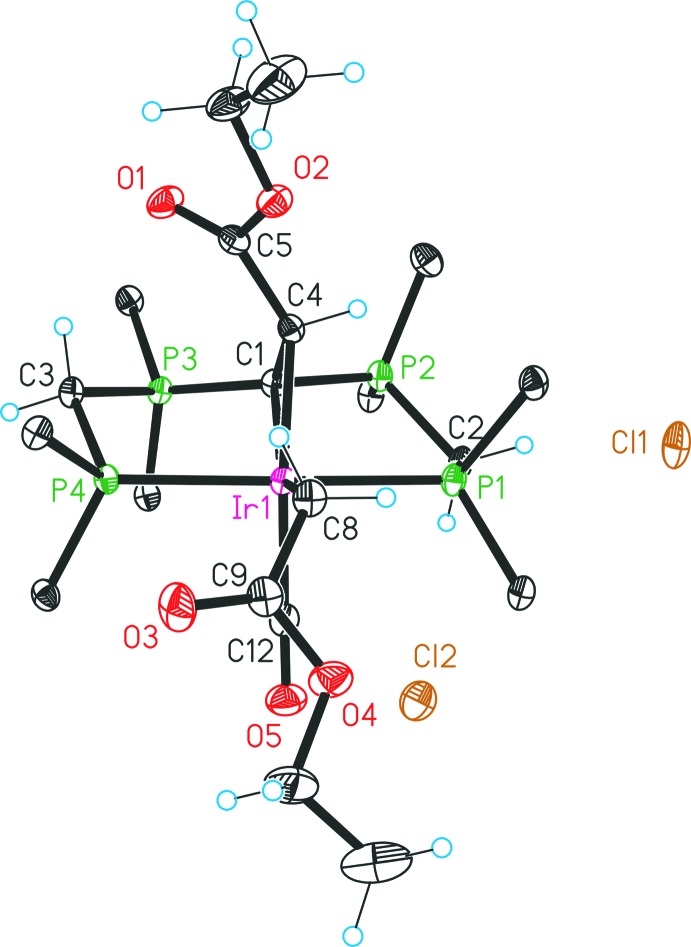
Mol­ecular structure of the complex cation in **7** and the two counter-ions. Displacement ellipsoids are drawn at the 30% probability level. For clarity, only the *ipso* carbon atoms of the phenyl groups are presented and the solvent mol­ecules are omitted.

Finally, an elimination of the chlorido ligand of complex **5** and its replacement by a carbonyl ligand results in compound **7**, [Ir^III^{C(CHCO_2_Et)(dppm)_2_-κ^4^ *P*,*C*,*C*′,*P*′}(CH_2_CO_2_Et)(CO)]Cl_2_ (Fig. 5 ▸). The asymmetric unit comprises one complex mol­ecule of **7** and additionally of two methyl­ene chloride solvent mol­ecules and 1.5 mol­ecules of water. In comparison with complex **5**, the structural features have not changed dramatically, with some slight variations for bond lengths and angles (Table 1 ▸).

## Supra­molecular features   

In all crystal structures, the complex cations and counter-ions are packed in a way that leaves voids for various types of solvent mol­ecules. Weak non-classical hydrogen-bonding inter­actions are observed between complex cations, chloride counter-ions and solvent mol­ecules. Numerical details of these inter­actions are given in Tables 2 ▸–5 ▸ ▸ ▸, and discussed briefly below.

**Figure 6:**
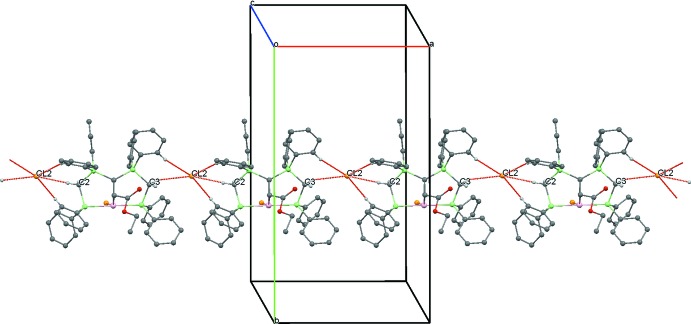
A view along the *c* axis of the crystal packing of compound **4**. Only the H atoms involved in the most significant inter­molecular inter­actions (Table 2 ▸) are displayed and the intra­molecular inter­action is omitted.

In the structure of **4**, there are weak C—H⋯Cl inter­actions between the chloride counter-ion and the methyl­ene groups of the PCP pincer ligand system [Cl2⋯H2*B* = 2.58 Å, H3*B*⋯Cl2(*x* − 1, *y*, *z*) = 2.83 Å] exhibiting distances shorter than the sum of the van der Waals radii (Table 2 ▸, Fig. 6 ▸). Such C—H⋯*X* inter­actions are a common feature of complexes containing dppm or related ligands (Jones & Ahrens, 1998 ▸).

**Figure 7:**
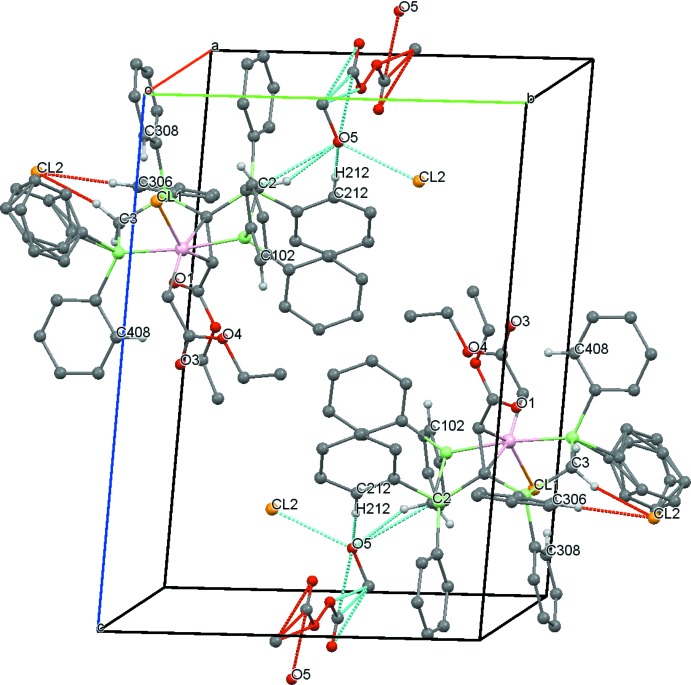
A view along the *a* axis of the crystal packing of compound **5**. Only the H atoms involved in the most significant inter­molecular inter­actions (Table 3 ▸) are presented and the intra­molecular inter­actions are omitted. One phenyl group and the solvent mol­ecules show positional disorder.

Moreover, compound **5** shows C—H⋯O and C—H⋯Cl inter­actions (Table 3 ▸) between the methyl­ene groups of the dppm moieties and the solvent mol­ecules and additionally the counter-ion, forming short contacts of 2.22 Å [H2*A*⋯O5 (*x*, *y* − 1, *z*)], 2.91 Å (H3*A*⋯Cl2) and 2.40 Å (H3*B*⋯O1) (Fig. 7 ▸).

**Figure 8:**
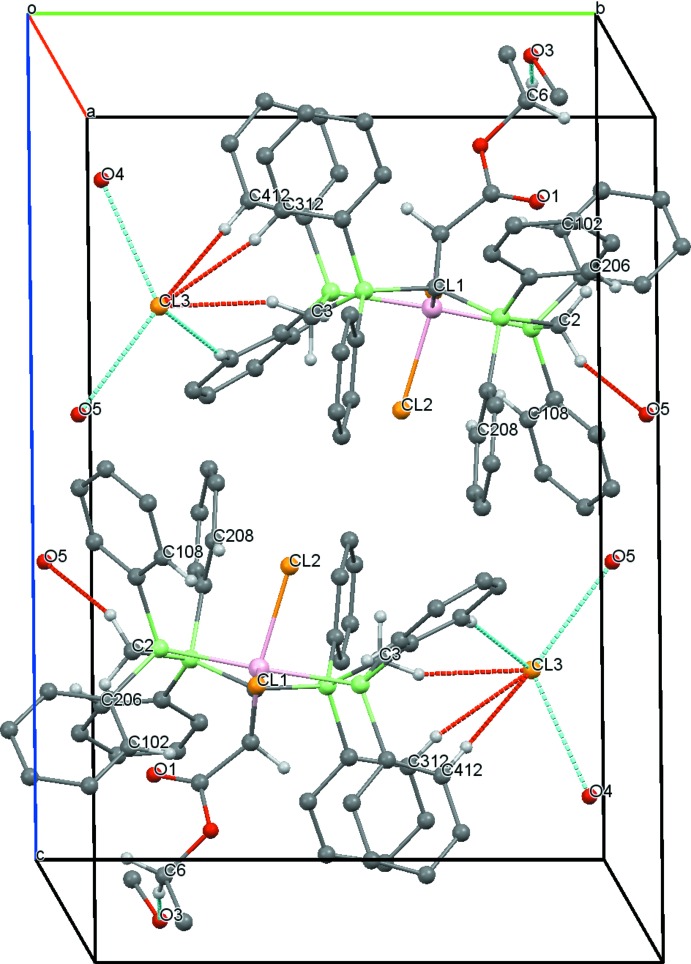
A view along the *a* axis of the crystal packing of compound **6**. Only the H atoms involved in the most significant inter­molecular inter­actions (Table 4 ▸) are presented and the intra­molecular inter­actions are omitted.

In the structure of **6**, the methyl­ene groups of the PCP unit and the chloride counter-ion and the solvent mol­ecules form C—H⋯O and C—H⋯Cl inter­actions (Table 4 ▸), exhibiting distances of 2.45 Å [H2*B*⋯O5(*x*, *y* − 1, *z*)], 2.66 Å (H3*B*⋯Cl3) and 2.40 Å [H6*A*⋯O3 (−*x* + 1, −*y*, −*z* + 2)] (Fig. 8 ▸).

**Figure 9:**
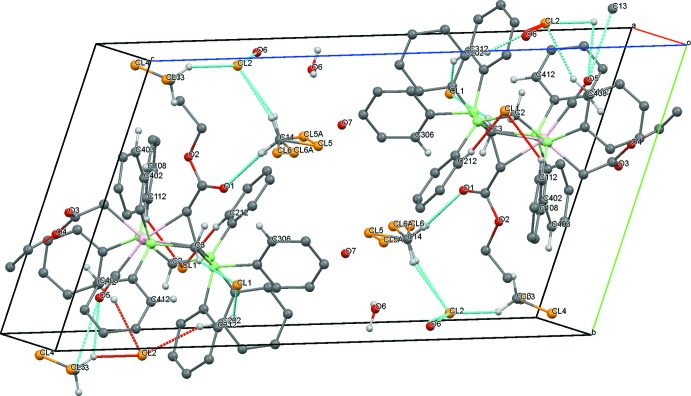
A view along the *a* axis of the crystal packing of compound **7**. Only the H atoms involved in the most significant inter­molecular inter­actions (Table 5 ▸) are presented and the intra­molecular inter­actions are omitted. The solvent mol­ecules are disordered.

In compound **7**, the chloride counter-ions inter­act with both the PCP pincer ligand system and the solvent mol­ecules. The solvent mol­ecules also show inter­actions with the iridium complex (Table 5 ▸, Fig. 9 ▸).

## Synthesis and crystallization   

Each reaction step was carried out under an atmosphere of nitro­gen by the use of standard Schlenk techniques. All starting materials and solvents were obtained from commercial suppliers, excluding the compound [CH(dppm)_2_]Cl that was prepared by a previously reported procedure (Reitsamer *et al.*, 2012 ▸). ^1^H-, ^13^C- and ^31^P-NMR spectra were recorded on a Bruker DPX 300 NMR spectrometer and were referenced against the ^13^C/^1^H peaks of deuterated solvents chloro­form and methanol or an external 85% H_3_PO_4_ standard, respectively. For the following assignment of the NMR data, atoms are labelled as in Figs. 2 ▸, 3 ▸, 4 ▸, 5 ▸.

**Synthesis of [Ir^III^{C(CHCO_2_Et)(dppm)_2_-** **κ^4^*P*,*C*,*C*′,*P*′}ClH]Cl·2.75CH_2_Cl_2_ (4)**: A mixture of [CH(dppm)_2_]Cl (0.0250 mmol, 20.4 mg) and [IrCl(cod)]_2_ (0.0125 mmol, 8.4 mg) was solved in 0.1 ml of MeCN. After a reaction time of one minute, a solution of ethyl diazo­acetate (0.0250 mmol, 2.85 mg) in MeOH (0.5 ml) was added. 10 min later, a deep yellow liquid was obtained. The volatiles were removed and the remaining solid was dissolved in methyl­ene chloride (0.6 ml), leading to complex **4** in high yield (0.0250 mmol, 28.3 mg). Single crystals of complex **4** were grown from a solvent mixture of *n*-hexane (1.2 ml) and CH_2_Cl_2_ (0.2 ml). ^31^P {^1^H} NMR (CHCl_3_): δ = 18.8 (*ddd*, P1, ^2^ *J*_P1P2_ = 16.9 Hz, ^4^ *J*_P1P3_ = 16.6 Hz, ^2^ *J*_P1P4_ = 399.2 Hz), 38.1 (*ddd*, P2, ^2^ *J*_P2P3_ = 38.3 Hz, ^4^ *J*_P2P4_ = 16.9 Hz), 34.7 (*ddd*, P3, ^2^ *J*_P3P4_ = 29.0 Hz), 10.7 (*ddd*, P4) ppm; ^1^H NMR (CDCl_3_/MeOH, 5:1): δ = −15.2 (*ddddd*, hydride, ^3^ *J*_P2H_ = 5.5 Hz, ^3^ *J*_P3H_ = 5.5 Hz, ^2^ *J*_P1H_ = 13.1 Hz, ^2^ *J*_P4H_ = 13.1 Hz, ^2^ *J*_C1H_ = 14.3 Hz) ppm; ^13^C {^1^H} NMR (CDCl_3_): δ = 3.6 (*dddd*, C1, ^1^ *J*_C1P2_ = 63.5 Hz, ^2^ *J*_C1P3_ = 74.6 Hz, ^2^ *J*_C1P1_ = 3.9 Hz, ^2^ *J*_C1P4_ = 3.9 Hz) ppm.

**Synthesis of [Ir^III^{C(CHCO_2_Et)(dppm)_2_-** **κ^4^*P*,*C*,*C*′,*P*′** **}(CH_2_CO_2_Et)Cl]Cl·CH_3_OH·0.5 H_2_O (5):** Ethyl diazo­acetate (0.116 mmol, 13.2 mg) was added to a solution of complex **4** (0.0250 mmol, 28.3 mg) in CH_2_Cl_2_ (0.6 ml), and the reaction mixture was stirred for 30 min. Complex **5** (0.0250 mmol, 30.7 mg) was formed qu­anti­tatively. Single crystals were obtained via slow evaporation of a 1:1methyl­ene chloride/methanol mixture. ^31^P {^1^H} NMR (CHCl_3_): δ = 0.3 (*ddd*, P1, ^2^ *J*_P1P2_ = 24.4 Hz, ^4^ *J*_P1P3_ = 10.6 Hz, ^2^ *J*_P1P4_ = 436.4 Hz), 40.6 (*dddd*, P2, ^2^ *J*_P2P3_ = 35.1 Hz, ^4^ *J*_P2P4_ = 15.3 Hz), 36.4 (*dddd*, P3, ^2^ *J*_P3P4_ = 15.9 Hz), −4.4 (*ddd*, P4) ppm; ^13^C {^1^H} NMR (CDCl_3_): δ = 3.1 (*dddd*, C1, ^1^ *J*_C1P2_ = 68.8 Hz, ^1^ *J*_C1P3_ = 55.6 Hz, ^2^ *J*_C1P1_ = 3.5 Hz, ^2^ *J*_C1P4_ = 3.5 Hz) ppm.

**Synthesis of [Ir^III^{C(CHCO_2_Et)(dppm)_2_-** **κ^4^*P*,*C*,*C*′,*P*′** **}Cl_2_]Cl·CH_3_OH·2H_2_O (6):** A solution of complex **4** (0.0250 mmol, 28.3 mg) in CH_2_Cl_2_ (0.6 ml) was treated with hydro­chloric acid (77.0 µl, 37%, 0.925 mmol) and stirred vigorously for approximately 10 min. The organic phase was separated and washed with water (0.5 ml) three times in total. Complex **6** (0.0250 mmol, 29.1 mg) was formed almost qu­anti­tatively. Yellow single crystals were generated by slow evaporation of a 1:1 solvent mixture of MeCN and MeOH. ^31^P {^1^H} NMR (CHCl_3_): δ = −6.1 (*ddd*, P1, ^2^ *J*_P1P2_ = 19.9 Hz, ^4^J_P1P3_ = 19.8 Hz, ^2^ *J*_P1P4_ = 452.1 Hz), 46.9 (*ddd*, P2, ^2^ *J*_P2P3_ = 38.3 Hz, ^4^ *J*_P2P4_ = 30.6 Hz), 45.8 (*ddd*, P3, ^2^ *J*_P3P4_ = 19.8 Hz), −10.3 (*ddd*, P4) ppm; ^13^C {^1^H} NMR (CDCl_3_): δ = 3.8 (*dd*, C1, ^1^ *J*_C1P2_ = 50.2 Hz, ^1^ *J*_C1P3_ = 50.2 Hz) ppm.

**Synthesis of [Ir^III^{C(CHCO_2_Et)(dppm)_2_-** **κ^4^*P*,*C*,*C*′,*P*′** **}(CH_2_CO_2_Et)(CO)]Cl_2_·2CH_2_Cl_2_·1.5H_2_O (7):** A solution of complex **5** (0.025 mmol, 29.1 mg) in CH_2_Cl_2_ was placed under an atmosphere of CO. After a reaction time of 1 h, complex **7** had formed qu­anti­tatively (0.0250 mmol, 31.1 mg). Single crystals were grown from a solution of methyl­ene chloride, covered with a small amount of ethyl acetate. ^31^P {^1^H} NMR (CH_2_Cl_2_): δ = −6.5 (*ddd*, P1, ^2^ *J*_P1P2_ = 14.2 Hz, ^4^ *J*_P1P3_ = 9.5 Hz, ^2^ *J*_P1P4_ = 339.8 Hz), 41.2 (*ddd*, P2, ^2^ *J*_P2P3_ = 27.7 Hz, ^4^ *J*_P2P4_ = 18.4 Hz), 39.7 (*ddd*, P3, ^2^ *J*_P3P4_ = 12.3 Hz), −16.4 (*ddd*, P4) ppm; ^13^C {^1^H} NMR (CD_2_Cl_2_): δ = 16.1 (*ddd*, C1, ^1^J_C1P2_ = 59.8 Hz, ^1^ *J*_C1P3_ = 49.3 Hz, ^2^ *J*_C1P4_ = 2.7 Hz, ^2^ *J*_C1C12_ = 1.5 Hz), 172.8 (*ddd*, C12, ^2^J_C12P1_ = 8.6 Hz, ^2^ *J*_C12P4_ = 8.6 Hz) ppm.

## Refinement   

Crystal data, data collection and structure refinement details are summarized in Table 6 ▸. Diffraction data for all crystals were measured by using multiple scans to increase the number of redundant reflections. We found the data of sufficient quality to proceed without semi-empirical absorption methods.

Unless noted otherwise, H atoms in the four structures were placed geometrically and refined in the riding-model approximation with *U*_iso_(H) = 1.2*U*_eq_(C) for phenyl and methyl­ene H atoms and 1.5*U*_eq_(C) for methyl H atoms.

For compound **4**, the two hydrogen atoms bound to the central Ir1 atom and the C4 atom of the eth­oxy­oxoethanyl­idene moiety were discernible from a difference-Fourier map. They were refined with bond-length restraints of 0.96 Å (C4) and 1.60 Å (Ir1) and with individual *U*_iso_ values. Three of the four methyl­ene chloride solvent mol­ecules are disordered. One solvent mol­ecule (C9, Cl3, Cl4) shows half-occupation, one (C12, Cl9, Cl10) is disordered around an inversion centre (occupancy 0.25) and for one (C11, Cl7, Cl8) the Cl atoms show a positional disorder over two sites (ratio 0.7:0.3). All H atoms of the solvent mol­ecules were omitted from the final model.

The scattering power of the crystal of compound **5** was poor. Hence, it was possible to collect reflections only up to 45°/2θ. The H atom attached to the C4 position was treated as described above. The methanol (C13, O6) and water (O7) solvent mol­ecules are disordered around an inversion centre and were refined with half-occupation. H atoms of the disordered solvent mol­ecules were omitted from the model. Furthermore, one phenyl group shows a 1:1 positional disorder and was refined over two sets of sites (C401–C406; C41**A**–C46**A**). All atoms of the disordered phenyl ring were refined isotropically.

In compounds **6** and **7**, the H atom attached to the C4 position was treated as described above. For **6**, localization of the H atoms of the methanol and water solvent mol­ecules was not possible and hence they were omitted from the model. For **7**, H atoms of water mol­ecule O6 were located from a difference-Fourier map and refined with bond-length restraints of 0.84 Å. The O7 atom of the other water mol­ecule was treated as being half-occupied, and its H atoms were omitted from the model. One methyl­ene chloride solvent mol­ecule (C14, Cl5, Cl6) was refined over two sets of sites (ratio 0.65:0.35).

## Supplementary Material

Crystal structure: contains datablock(s) global, 4, 5, 6, 7. DOI: 10.1107/S2056989018017024/wm5471sup1.cif

Structure factors: contains datablock(s) 4. DOI: 10.1107/S2056989018017024/wm54714sup6.hkl

Structure factors: contains datablock(s) 5. DOI: 10.1107/S2056989018017024/wm54715sup7.hkl

Structure factors: contains datablock(s) 6. DOI: 10.1107/S2056989018017024/wm54716sup8.hkl

Structure factors: contains datablock(s) 7. DOI: 10.1107/S2056989018017024/wm54717sup9.hkl

CCDC references: 1873392, 1873391, 1873390, 1873389

Additional supporting information:  crystallographic information; 3D view; checkCIF report

## Figures and Tables

**Table 1:** Selected bond lengths (Å) and angles (°) of the compounds **4**, **5**, **6** and **7**

	**4**	**5**	**6**	**7**
Ir1—C1	2.273 (4)	2.279 (6)	2.149 (4)	2.225 (3)
Ir1—C4	2.072 (5)	2.046 (7)	2.076 (4)	2.119 (3)
Ir1—P1	2.290 (1)	2.318 (2)	2.309 (1)	2.339 (1)
Ir1—P4	2.278 (1)	2.306 (2)	2.330 (1)	2.366 (1)
P2—C1	1.791 (5)	1.788 (7)	1.822 (4)	1.837 (3)
Ir1—*L*_*x*_ (*L*_*x*_= –H, –Cl, –CH_2_CO_2_Et)	1.62 (2)	2.163 (7)	2.427 (1)	2.177 (3)
Ir1—*L*_*y*_ (*L*_*y*_ = –Cl, –CO)	2.462 (1)	2.461 (2)	2.460 (1)	1.910 (3)
P3—C1	1.788 (5)	1.789 (7)	1.833 (4)	1.791 (3)
C1—C4	1.515 (6)	1.507 (9)	1.513 (5)	1.515 (4)
C4—Ir1—C1	40.5 (2)	40.3 (2)	41.9 (2)	40.72 (11)
C4—C1—Ir1	62.6 (2)	61.5 (3)	66.5 (2)	65.88 (15)
C1—C4—Ir1	76.9 (3)	78.2 (4)	71.6 (2)	73.40 (16)
C4—Ir1—*L*_*y*_ (*L*_*y*_ = –Cl, –CO)	150.3 (1)	152.5 (2)	151.9 (1)	158.8 (1)
C1—Ir1—*L*_*y*_ (*L*_*y*_ = –Cl, –CO)	111.3 (1)	112.8 (2)	111.5 (1)	118.8 (1)
C4—Ir1—*L*_*x*_ (*L*_*x*_ = –H, –Cl, –CH_2_CO_2_Et)	119.7 (18)	120.8 (3)	116.2 (1)	106.7 (1)
C1—Ir1—*L*_*x*_ (*L*_*x*_ = –H, –Cl, –CH_2_CO_2_Et)	159.8 (18)	161.1 (3)	158.1 (1)	147.4 (1)
P1—Ir1—P4	178.4 (1)	173.5 (1)	177.6 (1)	176.4 (1)
P1—Ir1—(C1—C4)	84.21	88.85	85.57	84.56

**Table 2:** Hydrogen-bond geometry (Å, °) for **4** [Chem scheme1]

*D*—H⋯*A*	*D*—H	H⋯*A*	*D*⋯*A*	*D*—H⋯*A*
C2—H2*B*⋯Cl2	0.98	2.58	3.488 (5)	154
C3—H3*A*⋯O1	0.98	2.31	2.892 (7)	117
C3—H3*B*⋯Cl2^i^	0.98	2.83	3.456 (5)	122

Symmetry code: (i) 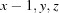.

**Table 3:** Hydrogen-bond geometry (Å, °) for **5** [Chem scheme1]

*D*—H⋯*A*	*D*—H	H⋯*A*	*D*⋯*A*	*D*—H⋯*A*
C2—H2*A*⋯O5^i^	0.98	2.22	3.139 (15)	156
C3—H3*A*⋯Cl2	0.98	2.91	3.693 (8)	137
C3—H3*B*⋯O1	0.98	2.40	2.895 (10)	111
C102—H102⋯O4	0.94	2.48	3.263 (11)	141
C212—H212⋯O5^i^	0.94	2.54	3.445 (18)	163
C306—H306⋯Cl2	0.94	2.57	3.491 (9)	167
C308—H308⋯Cl1	0.94	2.56	3.464 (8)	162
C408—H408⋯O3	0.94	2.23	3.046 (10)	145

Symmetry code: (i) 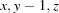.

**Table 4:** Hydrogen-bond geometry (Å, °) for **6** [Chem scheme1]

*D*—H⋯*A*	*D*—H	H⋯*A*	*D*⋯*A*	*D*—H⋯*A*
C2—H2*A*⋯O1	0.98	2.33	2.852 (5)	112
C2—H2*B*⋯O5^i^	0.98	2.45	3.320 (8)	148
C3—H3*B*⋯Cl3	0.98	2.66	3.589 (4)	158
C6—H6*A*⋯O3^ii^	0.98	2.40	3.369 (8)	169
C102—H102⋯Cl1	0.94	2.63	3.343 (4)	133
C108—H108⋯Cl1	0.94	2.82	3.671 (5)	151
C206—H206⋯Cl3^i^	0.94	2.87	3.742 (5)	156
C208—H208⋯Cl2	0.94	2.64	3.487 (5)	150
C312—H312⋯Cl3	0.94	2.84	3.749 (6)	164
C402—H402⋯Cl1	0.94	2.59	3.398 (6)	144
C406—H406⋯Cl3	0.94	2.88	3.757 (6)	156
C412—H412⋯Cl3	0.94	2.95	3.870 (5)	167

Symmetry codes: (i) 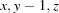; (ii) 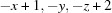.

**Table 5:** Hydrogen-bond geometry (Å, °) for **7** [Chem scheme1]

*D*—H⋯*A*	*D*—H	H⋯*A*	*D*⋯*A*	*D*—H⋯*A*
C2—H2*A*⋯Cl1	0.98	2.48	3.421 (3)	162
C3—H3*A*⋯Cl1^i^	0.98	2.59	3.488 (3)	152
C3—H3*B*⋯O1	0.98	2.21	2.968 (4)	134
C102—H102⋯Cl2	0.94	2.61	3.505 (4)	160
C108—H108⋯O2	0.94	2.61	3.313 (4)	132
C112—H112⋯Cl1	0.94	2.80	3.595 (4)	143
C202—H202⋯Cl2	0.94	2.70	3.574 (4)	156
C212—H212⋯Cl1	0.94	2.80	3.733 (5)	173
C306—H306⋯O1	0.94	2.47	3.061 (4)	121
C312—H312⋯Cl1^i^	0.94	2.73	3.503 (4)	140
C402—H402⋯O2	0.94	2.47	3.375 (4)	162
C408—H408⋯O3	0.94	2.44	3.326 (5)	156
C412—H412⋯Cl1^i^	0.94	2.97	3.866 (4)	161
C13—H13*A*⋯O5^ii^	0.98	2.58	3.194 (6)	121
C13—H13*A*⋯Cl2^ii^	0.98	2.68	3.500 (7)	141
C14—H14*A*⋯Cl2^iii^	0.98	2.65	3.553 (6)	153
C14—H14*B*⋯O1^iv^	0.98	2.37	3.327 (6)	164
C14*A*—H14*C*⋯O1^iv^	0.98	2.38	3.327 (6)	163
C14*A*—H14*D*⋯Cl2^iii^	0.98	2.59	3.553 (6)	168
O6—H6*OA*⋯Cl2	0.85 (2)	2.39 (4)	3.178 (5)	154 (7)
O6—H6*OB*⋯Cl1	0.85 (2)	2.39 (2)	3.239 (6)	178 (6)

Symmetry codes: (i) 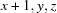; (ii) 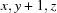; (iii) 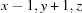; (iv) 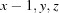.

**Table 6:** Experimental details

	**4**	**5**	**6**	**7**
Crystal data				
Chemical formula	[IrClH(C_55_H_50_O_2_P_4_)]Cl·2.75CH_2_Cl_2_	[Ir(C_4_H_7_O_2_)Cl(C_55_H_50_O_2_P_4_)]Cl·CH_4_O·0.5H_2_O	[IrCl_2_(C_55_H_50_O_2_P_4_)]Cl·CH_4_O·2H_2_O	[Ir(C_4_H_7_O_2_)(C_55_H_50_O_2_P_4_)(CO)]Cl_2_·2CH_2_Cl_2_·1.5H_2_O
*M*_r_	1364.48	1258.07	1233.45	1441.91
Crystal system, space group	Monoclinic, *P*2_1_/*n*	Triclinic, *P* 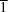	Triclinic, *P* 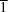	Triclinic, *P* 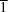
Temperature (K)	233	233	233	233
*a*, *b*, *c* (Å)	12.4425 (2), 22.4020 (3), 22.5393 (3)	12.4253 (3), 13.7081 (4), 17.6780 (6)	11.2371 (2), 12.9144 (2), 19.2371 (3)	11.7326 (2), 13.8815 (2), 22.2615 (3)
α, β, γ (°)	90, 94.826 (1), 90	93.152 (2), 97.960 (2), 103.771 (2)	89.439 (1), 77.863 (1), 83.114 (1)	75.477 (1), 86.508 (1), 65.212 (1)
*V* (Å^3^)	6260.26 (16)	2884.18 (15)	2709.27 (8)	3182.38 (9)
*Z*	4	2	2	2
Radiation type	Mo *K*α	Mo *K*α	Mo *K*α	Mo *K*α
μ (mm^−1^)	2.59	2.57	2.78	2.50
Crystal size (mm)	0.11 × 0.08 × 0.05	0.15 × 0.05 × 0.02	0.11 × 0.05 × 0.03	0.31 × 0.23 × 0.19

Data collection				
Diffractometer	Nonius KappaCCD	Nonius KappaCCD	Nonius KappaCCD	Nonius KappaCCD
No. of measured, independent and observed [*I* > 2σ(*I*)] reflections	39699, 11006, 8888	13821, 7453, 6326	17984, 9526, 8083	23329, 12496, 11695
*R*_int_	0.045	0.037	0.035	0.024
θ_max_ (°)	25.0	22.4	25.0	26.0

Refinement				
*R*[*F* ^2^ > 2σ(*F* ^2^)], *wR*(*F* ^2^), *S*	0.040, 0.112, 1.04	0.044, 0.106, 1.07	0.034, 0.073, 1.05	0.028, 0.070, 1.05
No. of reflections	11006	7453	9526	12496
No. of parameters	711	674	626	751
No. of restraints	2	1	1	3
H-atom treatment	H atoms treated by a mixture of independent and constrained refinement	H atoms treated by a mixture of independent and constrained refinement	H atoms treated by a mixture of independent and constrained refinement	H atoms treated by a mixture of independent and constrained refinement
Δρ_max_, Δρ_min_ (e Å^−3^)	1.03, −0.86	0.90, −0.96	0.75, −1.01	0.97, −1.29

Computer programs: *COLLECT* (Nonius, 1999 ▸), *DENZO* and *SCALEPACK* (Otwinowski & Minor, 1997 ▸), *XP* in *SHELXTL* and *SHELXS97* (Sheldrick, 2008 ▸), *SHELXL2014/7* (Sheldrick, 2015 ▸), (Sheldrick, 2008 ▸), *publCIF* (Westrip, 2010 ▸) and *CHEMDRAW* (Cambridge Soft, 2001 ▸).

## supplementary crystallographic information

### (Bis{[(diphenylphosphanyl)methyl]diphenylphosphanylidene}(ethoxyoxoethanylidene)methane-κ4P,C,C',P')chloridohydridoiridium(III) chloride methylene chloride 2.75-solvate (4) Crystal data

**Table:**

[IrClH(C_55_H_50_O_2_P_4_)]Cl·2.75CH_2_Cl_2_	*F*(000) = 2734
*M**_r_* = 1364.48	*D*_x_ = 1.448 Mg m^−^^3^
Monoclinic, *P*2_1_/*n*	Mo *K*α radiation, λ = 0.71073 Å
*a* = 12.4425 (2) Å	Cell parameters from 104186 reflections
*b* = 22.4020 (3) Å	θ = 1.0–25.3°
*c* = 22.5393 (3) Å	µ = 2.59 mm^−^^1^
β = 94.826 (1)°	*T* = 233 K
*V* = 6260.26 (16) Å^3^	Prism, colorless
*Z* = 4	0.11 × 0.08 × 0.05 mm

### (Bis{[(diphenylphosphanyl)methyl]diphenylphosphanylidene}(ethoxyoxoethanylidene)methane-κ4P,C,C',P')chloridohydridoiridium(III) chloride methylene chloride 2.75-solvate (4) Data collection

**Table:**

Nonius KappaCCD diffractometer	8888 reflections with *I* > 2σ(*I*)
Radiation source: fine-focus sealed tube	*R*_int_ = 0.045
Graphite monochromator	θ_max_ = 25.0°, θ_min_ = 1.8°
phi– and ω–scans	*h* = −14→14
39699 measured reflections	*k* = −26→26
11006 independent reflections	*l* = −26→26

### (Bis{[(diphenylphosphanyl)methyl]diphenylphosphanylidene}(ethoxyoxoethanylidene)methane-κ4P,C,C',P')chloridohydridoiridium(III) chloride methylene chloride 2.75-solvate (4) Refinement

**Table:**

Refinement on *F*^2^	2 restraints
Least-squares matrix: full	Hydrogen site location: mixed
*R*[*F*^2^ > 2σ(*F*^2^)] = 0.040	H atoms treated by a mixture of independent and constrained refinement
*wR*(*F*^2^) = 0.112	*w* = 1/[σ^2^(*F*_o_^2^) + (0.0606*P*)^2^ + 10.2768*P*] where *P* = (*F*_o_^2^ + 2*F*_c_^2^)/3
*S* = 1.04	(Δ/σ)_max_ = 0.001
11006 reflections	Δρ_max_ = 1.03 e Å^−^^3^
711 parameters	Δρ_min_ = −0.86 e Å^−^^3^

### (Bis{[(diphenylphosphanyl)methyl]diphenylphosphanylidene}(ethoxyoxoethanylidene)methane-κ4P,C,C',P')chloridohydridoiridium(III) chloride methylene chloride 2.75-solvate (4) Special details

**Table:**

Geometry. All esds (except the esd in the dihedral angle between two l.s. planes) are estimated using the full covariance matrix. The cell esds are taken into account individually in the estimation of esds in distances, angles and torsion angles; correlations between esds in cell parameters are only used when they are defined by crystal symmetry. An approximate (isotropic) treatment of cell esds is used for estimating esds involving l.s. planes.
Refinement. Hyrogen atoms at Ir1 and C4 were localized and refined with bond restraints: 96 pm at C4 and 160 pm at Ir1, respectively. There are four solvent molecules into the asymmetric unit, which are partial disordered (C9 occupational disorder with factor 0.5, C11 positional disorder of chlorine atoms wiht ratio 7:3 and C12 occupational disorder with factor 0.25). Hydrogen atoms at solvent were omitted.

### (Bis{[(diphenylphosphanyl)methyl]diphenylphosphanylidene}(ethoxyoxoethanylidene)methane-κ4P,C,C',P')chloridohydridoiridium(III) chloride methylene chloride 2.75-solvate (4) Fractional atomic coordinates and isotropic or equivalent isotropic displacement parameters (Å^2^)

**Table:**

	*x*	*y*	*z*	*U*_iso_*/*U*_eq_	Occ. (<1)
Ir1	0.49207 (2)	0.11833 (2)	0.20622 (2)	0.03228 (8)	
H1	0.484 (4)	0.1906 (9)	0.209 (2)	0.067 (18)*	
P1	0.67637 (10)	0.11984 (5)	0.22113 (6)	0.0370 (3)	
P2	0.62189 (10)	−0.01107 (5)	0.22879 (6)	0.0365 (3)	
P3	0.36683 (10)	−0.01314 (5)	0.21373 (5)	0.0326 (3)	
P4	0.30844 (10)	0.11776 (5)	0.19399 (6)	0.0322 (3)	
Cl1	0.52098 (11)	0.12563 (6)	0.09975 (6)	0.0461 (3)	
Cl2	0.98655 (13)	0.01266 (9)	0.20289 (13)	0.1081 (9)	
O1	0.3419 (3)	0.04012 (18)	0.33389 (18)	0.0627 (11)	
O2	0.4452 (4)	0.11608 (16)	0.36960 (17)	0.0579 (11)	
C1	0.4926 (4)	0.02169 (19)	0.2370 (2)	0.0323 (10)	
C2	0.7115 (4)	0.0439 (2)	0.1997 (2)	0.0424 (12)	
H2A	0.7070	0.0410	0.1562	0.051*	
H2B	0.7860	0.0352	0.2148	0.051*	
C3	0.2600 (4)	0.0420 (2)	0.2099 (2)	0.0396 (11)	
H3A	0.2270	0.0425	0.2479	0.048*	
H3B	0.2042	0.0304	0.1788	0.048*	
C4	0.4981 (4)	0.0697 (2)	0.2846 (2)	0.0360 (11)	
H4	0.571 (2)	0.068 (2)	0.3031 (19)	0.036 (13)*	
C5	0.4185 (5)	0.0725 (2)	0.3304 (2)	0.0443 (12)	
C7	0.3729 (7)	0.1253 (3)	0.4152 (3)	0.076 (2)	
H7A	0.3676	0.0890	0.4392	0.091*	
H7B	0.3007	0.1357	0.3975	0.091*	
C8	0.4203 (8)	0.1761 (4)	0.4533 (4)	0.118 (3)	
H8A	0.3744	0.1842	0.4851	0.177*	
H8B	0.4918	0.1650	0.4704	0.177*	
H8C	0.4252	0.2115	0.4289	0.177*	
C101	0.7549 (4)	0.1701 (2)	0.1780 (2)	0.0463 (13)	
C102	0.8362 (7)	0.1526 (4)	0.1455 (5)	0.115 (4)	
H102	0.8538	0.1119	0.1438	0.138*	
C103	0.8944 (8)	0.1940 (5)	0.1145 (6)	0.149 (5)	
H103	0.9491	0.1811	0.0911	0.179*	
C104	0.8713 (6)	0.2521 (4)	0.1183 (4)	0.095 (3)	
H104	0.9105	0.2800	0.0978	0.114*	
C105	0.7917 (8)	0.2712 (3)	0.1516 (4)	0.093 (3)	
H105	0.7776	0.3123	0.1549	0.112*	
C106	0.7311 (7)	0.2298 (3)	0.1808 (3)	0.080 (2)	
H106	0.6740	0.2427	0.2024	0.096*	
C107	0.7388 (5)	0.1311 (2)	0.2962 (3)	0.0514 (14)	
C108	0.8387 (6)	0.1063 (3)	0.3146 (3)	0.073 (2)	
H108	0.8762	0.0827	0.2886	0.088*	
C109	0.8822 (8)	0.1176 (4)	0.3736 (5)	0.112 (4)	
H109	0.9490	0.1009	0.3874	0.134*	
C110	0.8274 (11)	0.1528 (5)	0.4107 (4)	0.115 (4)	
H110	0.8576	0.1599	0.4497	0.138*	
C111	0.7302 (8)	0.1776 (4)	0.3923 (3)	0.091 (3)	
H111	0.6938	0.2020	0.4181	0.110*	
C112	0.6863 (6)	0.1664 (3)	0.3353 (3)	0.0651 (17)	
H112	0.6190	0.1831	0.3225	0.078*	
C201	0.6228 (4)	−0.0736 (2)	0.1787 (2)	0.0409 (12)	
C202	0.6137 (5)	−0.0644 (3)	0.1167 (2)	0.0499 (14)	
H202	0.6052	−0.0255	0.1014	0.060*	
C203	0.6174 (6)	−0.1124 (3)	0.0783 (3)	0.0681 (18)	
H203	0.6104	−0.1063	0.0369	0.082*	
C204	0.6316 (5)	−0.1700 (3)	0.1014 (3)	0.0673 (18)	
H204	0.6355	−0.2027	0.0755	0.081*	
C205	0.6399 (5)	−0.1793 (3)	0.1620 (3)	0.0648 (17)	
H205	0.6478	−0.2183	0.1771	0.078*	
C206	0.6368 (4)	−0.1317 (2)	0.2010 (3)	0.0524 (14)	
H206	0.6441	−0.1383	0.2423	0.063*	
C207	0.6860 (5)	−0.0357 (2)	0.2990 (3)	0.0491 (14)	
C208	0.7957 (5)	−0.0481 (3)	0.3027 (3)	0.0682 (18)	
H208	0.8347	−0.0441	0.2690	0.082*	
C209	0.8477 (8)	−0.0666 (4)	0.3568 (5)	0.100 (3)	
H209	0.9219	−0.0752	0.3592	0.120*	
C210	0.7930 (10)	−0.0726 (4)	0.4060 (4)	0.106 (4)	
H210	0.8299	−0.0842	0.4422	0.127*	
C211	0.6813 (9)	−0.0614 (3)	0.4032 (3)	0.096 (3)	
H211	0.6435	−0.0664	0.4373	0.116*	
C212	0.6259 (6)	−0.0425 (3)	0.3485 (3)	0.0657 (18)	
H212	0.5514	−0.0347	0.3457	0.079*	
C301	0.3231 (4)	−0.0735 (2)	0.2590 (2)	0.0384 (11)	
C302	0.2144 (5)	−0.0820 (3)	0.2652 (3)	0.0572 (15)	
H302	0.1637	−0.0544	0.2486	0.069*	
C303	0.1802 (6)	−0.1309 (3)	0.2959 (3)	0.077 (2)	
H303	0.1062	−0.1366	0.2996	0.092*	
C304	0.2540 (7)	−0.1712 (3)	0.3209 (3)	0.078 (2)	
H304	0.2303	−0.2040	0.3423	0.094*	
C305	0.3607 (6)	−0.1639 (3)	0.3150 (3)	0.0711 (19)	
H305	0.4110	−0.1917	0.3319	0.085*	
C306	0.3953 (5)	−0.1149 (2)	0.2835 (3)	0.0554 (15)	
H306	0.4692	−0.1100	0.2791	0.067*	
C307	0.3642 (4)	−0.0446 (2)	0.1398 (2)	0.0356 (11)	
C308	0.3715 (5)	−0.0068 (2)	0.0913 (2)	0.0494 (13)	
H308	0.3852	0.0341	0.0973	0.059*	
C309	0.3585 (6)	−0.0297 (3)	0.0343 (3)	0.0690 (18)	
H309	0.3634	−0.0042	0.0015	0.083*	
C310	0.3382 (6)	−0.0895 (3)	0.0250 (3)	0.0686 (18)	
H310	0.3291	−0.1048	−0.0140	0.082*	
C311	0.3314 (6)	−0.1269 (3)	0.0729 (3)	0.0625 (17)	
H311	0.3179	−0.1678	0.0667	0.075*	
C312	0.3444 (5)	−0.1046 (2)	0.1300 (2)	0.0472 (13)	
H312	0.3397	−0.1305	0.1626	0.057*	
C401	0.2343 (4)	0.1642 (2)	0.2433 (2)	0.0402 (11)	
C402	0.2883 (5)	0.2051 (2)	0.2807 (2)	0.0483 (13)	
H402	0.3637	0.2086	0.2817	0.058*	
C403	0.2304 (5)	0.2412 (3)	0.3171 (3)	0.0607 (16)	
H403	0.2671	0.2690	0.3426	0.073*	
C404	0.1209 (5)	0.2365 (3)	0.3161 (3)	0.0636 (17)	
H404	0.0827	0.2606	0.3412	0.076*	
C405	0.0667 (5)	0.1966 (3)	0.2782 (4)	0.078 (2)	
H405	−0.0088	0.1936	0.2772	0.094*	
C406	0.1231 (5)	0.1603 (3)	0.2410 (3)	0.0690 (19)	
H406	0.0858	0.1334	0.2147	0.083*	
C407	0.2442 (4)	0.1381 (2)	0.1209 (2)	0.0421 (12)	
C408	0.2823 (5)	0.1899 (2)	0.0957 (2)	0.0507 (14)	
H408	0.3416	0.2100	0.1150	0.061*	
C409	0.2344 (6)	0.2122 (3)	0.0426 (3)	0.0676 (18)	
H409	0.2597	0.2478	0.0268	0.081*	
C410	0.1496 (6)	0.1820 (4)	0.0132 (3)	0.080 (2)	
H410	0.1162	0.1971	−0.0227	0.096*	
C411	0.1144 (7)	0.1301 (4)	0.0363 (4)	0.093 (3)	
H411	0.0587	0.1087	0.0151	0.111*	
C412	0.1591 (6)	0.1078 (3)	0.0910 (3)	0.0708 (19)	
H412	0.1317	0.0728	0.1072	0.085*	
C9	0.250 (3)	−0.0515 (13)	0.4305 (9)	0.223 (19)	0.5
Cl3	0.3575 (9)	−0.0543 (4)	0.4754 (3)	0.201 (4)	0.5
Cl4	0.1305 (7)	−0.0182 (4)	0.4559 (3)	0.167 (3)	0.5
C10	0.5629 (8)	0.2613 (4)	0.0386 (4)	0.112 (3)	
Cl5	0.4809 (2)	0.31145 (11)	0.07504 (11)	0.1086 (7)	
Cl6	0.6123 (3)	0.29238 (12)	−0.02166 (12)	0.1243 (9)	
C11	0.9453 (10)	−0.1288 (5)	0.1519 (7)	0.139 (4)	
Cl7	0.9402 (6)	−0.1902 (3)	0.1966 (6)	0.197 (4)	0.7
Cl8	1.0617 (3)	−0.1332 (3)	0.1087 (2)	0.1348 (16)	0.7
Cl7A	0.9153 (19)	−0.1694 (9)	0.231 (2)	0.31 (2)	0.3
Cl8A	1.0197 (18)	−0.1825 (9)	0.1312 (12)	0.239 (10)	0.3
C12	0.951 (5)	0.0067 (16)	0.026 (3)	0.16 (2)	0.25
Cl9	1.0704 (12)	−0.0453 (9)	0.0654 (12)	0.238 (11)	0.25
Cl10	0.8652 (16)	0.0098 (9)	0.0607 (12)	0.234 (11)	0.25

### (Bis{[(diphenylphosphanyl)methyl]diphenylphosphanylidene}(ethoxyoxoethanylidene)methane-κ4P,C,C',P')chloridohydridoiridium(III) chloride methylene chloride 2.75-solvate (4) Atomic displacement parameters (Å^2^)

**Table:**

	*U*^11^	*U*^22^	*U*^33^	*U*^12^	*U*^13^	*U*^23^
Ir1	0.03166 (12)	0.02717 (12)	0.03855 (12)	0.00054 (7)	0.00623 (8)	0.00117 (8)
P1	0.0320 (7)	0.0297 (7)	0.0491 (8)	−0.0032 (5)	0.0014 (6)	0.0026 (5)
P2	0.0328 (7)	0.0302 (6)	0.0466 (7)	0.0015 (5)	0.0025 (5)	0.0026 (6)
P3	0.0327 (6)	0.0274 (6)	0.0387 (7)	−0.0006 (5)	0.0085 (5)	0.0005 (5)
P4	0.0309 (6)	0.0272 (6)	0.0392 (7)	0.0035 (5)	0.0069 (5)	−0.0008 (5)
Cl1	0.0470 (7)	0.0514 (8)	0.0414 (7)	0.0001 (6)	0.0130 (6)	0.0066 (6)
Cl2	0.0366 (9)	0.0594 (11)	0.229 (3)	−0.0004 (7)	0.0143 (12)	−0.0035 (13)
O1	0.071 (3)	0.059 (3)	0.063 (3)	−0.014 (2)	0.029 (2)	−0.008 (2)
O2	0.082 (3)	0.051 (2)	0.043 (2)	−0.0040 (19)	0.017 (2)	−0.0143 (18)
C1	0.036 (3)	0.026 (2)	0.035 (3)	−0.0005 (19)	0.007 (2)	0.002 (2)
C2	0.030 (3)	0.034 (3)	0.064 (3)	0.000 (2)	0.007 (2)	0.003 (2)
C3	0.032 (3)	0.035 (3)	0.052 (3)	0.001 (2)	0.009 (2)	−0.001 (2)
C4	0.037 (3)	0.029 (3)	0.042 (3)	0.000 (2)	0.004 (2)	0.000 (2)
C5	0.054 (3)	0.039 (3)	0.041 (3)	0.002 (3)	0.011 (2)	0.002 (2)
C7	0.105 (6)	0.079 (5)	0.049 (4)	0.004 (4)	0.034 (4)	−0.014 (3)
C8	0.153 (9)	0.120 (7)	0.087 (6)	−0.003 (6)	0.036 (6)	−0.058 (6)
C101	0.036 (3)	0.045 (3)	0.056 (3)	−0.006 (2)	−0.002 (2)	0.010 (3)
C102	0.087 (6)	0.064 (5)	0.206 (10)	0.008 (4)	0.085 (6)	0.036 (6)
C103	0.112 (8)	0.098 (7)	0.254 (14)	0.014 (6)	0.117 (9)	0.062 (8)
C104	0.063 (5)	0.103 (7)	0.117 (7)	−0.036 (5)	0.000 (5)	0.047 (6)
C105	0.128 (7)	0.047 (4)	0.106 (6)	−0.020 (4)	0.020 (6)	0.022 (4)
C106	0.112 (6)	0.045 (4)	0.089 (5)	−0.006 (4)	0.039 (5)	0.007 (4)
C107	0.050 (3)	0.043 (3)	0.059 (4)	−0.015 (3)	−0.011 (3)	0.008 (3)
C108	0.063 (4)	0.069 (4)	0.084 (5)	−0.009 (3)	−0.025 (4)	0.009 (4)
C109	0.095 (7)	0.098 (7)	0.129 (9)	−0.027 (5)	−0.072 (7)	0.031 (6)
C110	0.149 (10)	0.114 (8)	0.074 (6)	−0.053 (7)	−0.027 (6)	−0.005 (6)
C111	0.115 (7)	0.091 (6)	0.066 (5)	−0.045 (5)	−0.004 (5)	−0.007 (4)
C112	0.076 (4)	0.057 (4)	0.062 (4)	−0.022 (3)	0.002 (3)	−0.009 (3)
C201	0.032 (3)	0.033 (3)	0.059 (3)	0.001 (2)	0.009 (2)	−0.006 (2)
C202	0.057 (4)	0.043 (3)	0.052 (3)	−0.001 (3)	0.018 (3)	−0.001 (3)
C203	0.071 (4)	0.072 (5)	0.064 (4)	−0.007 (3)	0.024 (3)	−0.017 (3)
C204	0.066 (4)	0.051 (4)	0.088 (5)	−0.004 (3)	0.024 (4)	−0.028 (4)
C205	0.058 (4)	0.039 (3)	0.099 (5)	0.003 (3)	0.015 (4)	−0.009 (3)
C206	0.041 (3)	0.039 (3)	0.077 (4)	0.002 (2)	0.008 (3)	−0.001 (3)
C207	0.055 (3)	0.035 (3)	0.054 (3)	0.002 (2)	−0.013 (3)	0.002 (2)
C208	0.055 (4)	0.055 (4)	0.090 (5)	0.005 (3)	−0.020 (3)	0.010 (3)
C209	0.089 (6)	0.074 (5)	0.126 (8)	0.009 (4)	−0.060 (6)	0.015 (5)
C210	0.154 (9)	0.064 (5)	0.086 (6)	0.037 (5)	−0.066 (6)	−0.004 (5)
C211	0.171 (9)	0.064 (5)	0.052 (4)	0.025 (5)	−0.005 (5)	0.002 (3)
C212	0.092 (5)	0.046 (3)	0.057 (4)	0.016 (3)	−0.006 (3)	0.010 (3)
C301	0.047 (3)	0.035 (3)	0.034 (3)	−0.005 (2)	0.011 (2)	−0.002 (2)
C302	0.053 (4)	0.050 (3)	0.071 (4)	−0.005 (3)	0.016 (3)	0.013 (3)
C303	0.076 (5)	0.070 (5)	0.091 (5)	−0.016 (4)	0.034 (4)	0.014 (4)
C304	0.102 (6)	0.056 (4)	0.079 (5)	−0.018 (4)	0.028 (4)	0.022 (4)
C305	0.093 (5)	0.047 (4)	0.073 (4)	0.003 (4)	0.007 (4)	0.017 (3)
C306	0.064 (4)	0.045 (3)	0.059 (4)	0.002 (3)	0.016 (3)	0.012 (3)
C307	0.034 (3)	0.034 (3)	0.040 (3)	0.002 (2)	0.009 (2)	0.000 (2)
C308	0.068 (4)	0.040 (3)	0.042 (3)	−0.002 (3)	0.015 (3)	0.001 (2)
C309	0.103 (5)	0.060 (4)	0.046 (3)	−0.008 (4)	0.017 (3)	0.003 (3)
C310	0.094 (5)	0.070 (4)	0.044 (3)	−0.005 (4)	0.016 (3)	−0.018 (3)
C311	0.082 (5)	0.052 (4)	0.055 (4)	−0.014 (3)	0.017 (3)	−0.022 (3)
C312	0.058 (3)	0.038 (3)	0.048 (3)	−0.003 (2)	0.015 (3)	−0.004 (2)
C401	0.041 (3)	0.034 (3)	0.047 (3)	0.001 (2)	0.013 (2)	−0.002 (2)
C402	0.047 (3)	0.048 (3)	0.050 (3)	0.008 (3)	0.008 (3)	−0.006 (3)
C403	0.064 (4)	0.058 (4)	0.060 (4)	0.013 (3)	0.005 (3)	−0.021 (3)
C404	0.072 (4)	0.045 (3)	0.079 (4)	0.008 (3)	0.035 (4)	−0.011 (3)
C405	0.053 (4)	0.061 (4)	0.127 (6)	−0.003 (3)	0.046 (4)	−0.031 (4)
C406	0.049 (4)	0.057 (4)	0.104 (5)	−0.011 (3)	0.030 (3)	−0.036 (4)
C407	0.039 (3)	0.043 (3)	0.045 (3)	0.010 (2)	0.004 (2)	−0.003 (2)
C408	0.056 (3)	0.046 (3)	0.050 (3)	0.008 (3)	0.003 (3)	0.002 (3)
C409	0.079 (5)	0.062 (4)	0.062 (4)	0.013 (4)	0.006 (4)	0.010 (3)
C410	0.087 (5)	0.092 (6)	0.059 (4)	0.013 (4)	−0.011 (4)	0.010 (4)
C411	0.076 (5)	0.115 (7)	0.080 (5)	−0.014 (5)	−0.034 (4)	−0.003 (5)
C412	0.062 (4)	0.076 (5)	0.070 (4)	−0.010 (3)	−0.021 (3)	0.004 (3)
C9	0.41 (4)	0.20 (2)	0.070 (12)	−0.24 (3)	0.11 (2)	−0.077 (14)
Cl3	0.324 (12)	0.154 (6)	0.121 (5)	0.103 (7)	0.006 (6)	−0.008 (5)
Cl4	0.197 (7)	0.205 (7)	0.099 (4)	−0.039 (6)	0.008 (4)	−0.037 (4)
C10	0.142 (8)	0.072 (5)	0.126 (8)	−0.001 (5)	0.032 (6)	0.029 (5)
Cl5	0.1132 (17)	0.1046 (17)	0.1117 (17)	−0.0339 (13)	0.0310 (14)	−0.0231 (13)
Cl6	0.160 (2)	0.1072 (18)	0.1116 (18)	0.0022 (17)	0.0446 (17)	0.0005 (15)
C11	0.101 (8)	0.117 (9)	0.195 (13)	0.018 (7)	−0.007 (8)	−0.027 (8)
Cl7	0.103 (5)	0.089 (4)	0.393 (14)	−0.018 (3)	−0.010 (6)	0.043 (6)
Cl8	0.088 (3)	0.194 (5)	0.118 (3)	0.029 (3)	−0.020 (2)	−0.020 (3)
Cl7A	0.128 (13)	0.102 (12)	0.69 (7)	0.008 (9)	0.05 (2)	−0.04 (2)
Cl8A	0.201 (19)	0.174 (15)	0.32 (3)	0.048 (14)	−0.098 (18)	−0.041 (16)
C12	0.22 (6)	0.04 (2)	0.22 (6)	−0.08 (3)	0.03 (4)	−0.05 (3)
Cl9	0.120 (11)	0.208 (18)	0.39 (3)	−0.022 (11)	0.010 (14)	−0.15 (2)
Cl10	0.159 (14)	0.213 (19)	0.31 (3)	0.077 (14)	−0.088 (16)	−0.088 (18)

### (Bis{[(diphenylphosphanyl)methyl]diphenylphosphanylidene}(ethoxyoxoethanylidene)methane-κ4P,C,C',P')chloridohydridoiridium(III) chloride methylene chloride 2.75-solvate (4) Geometric parameters (Å, º)

**Table:**

Ir1—C4	2.072 (5)	C207—C208	1.390 (8)
Ir1—C1	2.273 (4)	C207—C212	1.402 (9)
Ir1—P4	2.2783 (12)	C208—C209	1.394 (10)
Ir1—P1	2.2897 (13)	C208—H208	0.9400
Ir1—Cl1	2.4619 (13)	C209—C210	1.355 (13)
Ir1—H1	1.62 (2)	C209—H209	0.9400
P1—C107	1.819 (6)	C210—C211	1.408 (13)
P1—C101	1.824 (5)	C210—H210	0.9400
P1—C2	1.833 (5)	C211—C212	1.426 (9)
P2—C1	1.791 (5)	C211—H211	0.9400
P2—C207	1.798 (5)	C212—H212	0.9400
P2—C201	1.800 (5)	C301—C306	1.374 (8)
P2—C2	1.820 (5)	C301—C302	1.384 (7)
P3—C1	1.788 (5)	C302—C303	1.380 (9)
P3—C301	1.806 (5)	C302—H302	0.9400
P3—C307	1.807 (5)	C303—C304	1.376 (10)
P3—C3	1.811 (5)	C303—H303	0.9400
P4—C407	1.827 (5)	C304—C305	1.356 (10)
P4—C401	1.828 (5)	C304—H304	0.9400
P4—C3	1.846 (5)	C305—C306	1.396 (8)
O1—C5	1.205 (6)	C305—H305	0.9400
O2—C5	1.341 (6)	C306—H306	0.9400
O2—C7	1.437 (7)	C307—C312	1.383 (7)
C1—C4	1.515 (6)	C307—C308	1.391 (7)
C2—H2A	0.9800	C308—C309	1.380 (8)
C2—H2B	0.9800	C308—H308	0.9400
C3—H3A	0.9800	C309—C310	1.376 (9)
C3—H3B	0.9800	C309—H309	0.9400
C4—C5	1.490 (7)	C310—C311	1.375 (9)
C4—H4	0.968 (19)	C310—H310	0.9400
C7—C8	1.516 (10)	C311—C312	1.378 (8)
C7—H7A	0.9800	C311—H311	0.9400
C7—H7B	0.9800	C312—H312	0.9400
C8—H8A	0.9700	C401—C402	1.381 (7)
C8—H8B	0.9700	C401—C406	1.383 (8)
C8—H8C	0.9700	C402—C403	1.395 (8)
C101—C102	1.356 (9)	C402—H402	0.9400
C101—C106	1.371 (8)	C403—C404	1.364 (9)
C102—C103	1.399 (11)	C403—H403	0.9400
C102—H102	0.9400	C404—C405	1.375 (9)
C103—C104	1.337 (13)	C404—H404	0.9400
C103—H103	0.9400	C405—C406	1.397 (8)
C104—C105	1.360 (12)	C405—H405	0.9400
C104—H104	0.9400	C406—H406	0.9400
C105—C106	1.396 (10)	C407—C412	1.385 (8)
C105—H105	0.9400	C407—C408	1.392 (8)
C106—H106	0.9400	C408—C409	1.383 (8)
C107—C112	1.389 (9)	C408—H408	0.9400
C107—C108	1.393 (9)	C409—C410	1.376 (10)
C108—C109	1.416 (12)	C409—H409	0.9400
C108—H108	0.9400	C410—C411	1.362 (11)
C109—C110	1.372 (15)	C410—H410	0.9400
C109—H109	0.9400	C411—C412	1.402 (10)
C110—C111	1.364 (14)	C411—H411	0.9400
C110—H110	0.9400	C412—H412	0.9400
C111—C112	1.376 (10)	C9—Cl3	1.61 (4)
C111—H111	0.9400	C9—Cl4	1.80 (4)
C112—H112	0.9400	C10—Cl6	1.687 (9)
C201—C206	1.399 (7)	C10—Cl5	1.766 (10)
C201—C202	1.409 (8)	C11—Cl8A	1.61 (2)
C202—C203	1.384 (8)	C11—Cl7	1.709 (18)
C202—H202	0.9400	C11—Cl8	1.815 (15)
C203—C204	1.399 (9)	C11—Cl7A	2.07 (4)
C203—H203	0.9400	Cl8—Cl9	2.21 (3)
C204—C205	1.377 (10)	C12—Cl10	1.37 (5)
C204—H204	0.9400	C12—C12^i^	1.79 (12)
C205—C206	1.384 (8)	C12—Cl9	2.02 (7)
C205—H205	0.9400	C12—Cl9^i^	2.24 (6)
C206—H206	0.9400	Cl9—C12^i^	2.24 (6)
			
C4—Ir1—C1	40.49 (17)	C203—C202—H202	119.9
C4—Ir1—P4	93.68 (14)	C201—C202—H202	119.9
C1—Ir1—P4	90.52 (12)	C202—C203—C204	119.6 (6)
C4—Ir1—P1	85.34 (14)	C202—C203—H203	120.2
C1—Ir1—P1	89.54 (12)	C204—C203—H203	120.2
P4—Ir1—P1	178.42 (5)	C205—C204—C203	120.4 (6)
C4—Ir1—Cl1	150.26 (13)	C205—C204—H204	119.8
C1—Ir1—Cl1	111.34 (12)	C203—C204—H204	119.8
P4—Ir1—Cl1	96.30 (4)	C204—C205—C206	120.6 (6)
P1—Ir1—Cl1	85.15 (5)	C204—C205—H205	119.7
C4—Ir1—H1	119.7 (18)	C206—C205—H205	119.7
C1—Ir1—H1	159.8 (18)	C205—C206—C201	119.9 (6)
P4—Ir1—H1	87.0 (18)	C205—C206—H206	120.0
P1—Ir1—H1	92.4 (18)	C201—C206—H206	120.0
Cl1—Ir1—H1	88.8 (18)	C208—C207—C212	121.2 (6)
C107—P1—C101	101.8 (2)	C208—C207—P2	118.4 (5)
C107—P1—C2	106.3 (3)	C212—C207—P2	120.5 (5)
C101—P1—C2	106.4 (2)	C207—C208—C209	119.6 (8)
C107—P1—Ir1	118.9 (2)	C207—C208—H208	120.2
C101—P1—Ir1	120.39 (17)	C209—C208—H208	120.2
C2—P1—Ir1	101.87 (16)	C210—C209—C208	121.1 (8)
C1—P2—C207	111.7 (3)	C210—C209—H209	119.5
C1—P2—C201	115.9 (2)	C208—C209—H209	119.5
C207—P2—C201	106.7 (2)	C209—C210—C211	120.6 (7)
C1—P2—C2	110.1 (2)	C209—C210—H210	119.7
C207—P2—C2	106.6 (3)	C211—C210—H210	119.7
C201—P2—C2	105.3 (2)	C210—C211—C212	119.6 (8)
C1—P3—C301	117.3 (2)	C210—C211—H211	120.2
C1—P3—C307	112.8 (2)	C212—C211—H211	120.2
C301—P3—C307	104.2 (2)	C207—C212—C211	118.0 (7)
C1—P3—C3	109.7 (2)	C207—C212—H212	121.0
C301—P3—C3	106.4 (2)	C211—C212—H212	121.0
C307—P3—C3	105.4 (2)	C306—C301—C302	118.5 (5)
C407—P4—C401	101.6 (2)	C306—C301—P3	121.0 (4)
C407—P4—C3	106.2 (2)	C302—C301—P3	120.3 (4)
C401—P4—C3	102.5 (2)	C303—C302—C301	120.3 (6)
C407—P4—Ir1	117.78 (17)	C303—C302—H302	119.8
C401—P4—Ir1	118.39 (17)	C301—C302—H302	119.8
C3—P4—Ir1	108.78 (16)	C304—C303—C302	120.2 (7)
C5—O2—C7	116.2 (5)	C304—C303—H303	119.9
C4—C1—P3	120.2 (3)	C302—C303—H303	119.9
C4—C1—P2	112.1 (3)	C305—C304—C303	120.4 (6)
P3—C1—P2	124.2 (3)	C305—C304—H304	119.8
C4—C1—Ir1	62.6 (2)	C303—C304—H304	119.8
P3—C1—Ir1	110.2 (2)	C304—C305—C306	119.4 (7)
P2—C1—Ir1	109.8 (2)	C304—C305—H305	120.3
P2—C2—P1	111.5 (3)	C306—C305—H305	120.3
P2—C2—H2A	109.3	C301—C306—C305	121.1 (6)
P1—C2—H2A	109.3	C301—C306—H306	119.4
P2—C2—H2B	109.3	C305—C306—H306	119.4
P1—C2—H2B	109.3	C312—C307—C308	119.3 (5)
H2A—C2—H2B	108.0	C312—C307—P3	121.0 (4)
P3—C3—P4	112.7 (3)	C308—C307—P3	119.4 (4)
P3—C3—H3A	109.1	C309—C308—C307	119.7 (5)
P4—C3—H3A	109.1	C309—C308—H308	120.1
P3—C3—H3B	109.1	C307—C308—H308	120.1
P4—C3—H3B	109.1	C310—C309—C308	120.6 (6)
H3A—C3—H3B	107.8	C310—C309—H309	119.7
C5—C4—C1	121.8 (4)	C308—C309—H309	119.7
C5—C4—Ir1	126.1 (3)	C311—C310—C309	119.8 (6)
C1—C4—Ir1	76.9 (3)	C311—C310—H310	120.1
C5—C4—H4	111 (3)	C309—C310—H310	120.1
C1—C4—H4	105 (3)	C310—C311—C312	120.2 (6)
Ir1—C4—H4	111 (3)	C310—C311—H311	119.9
O1—C5—O2	123.2 (5)	C312—C311—H311	119.9
O1—C5—C4	126.5 (5)	C311—C312—C307	120.4 (5)
O2—C5—C4	110.2 (5)	C311—C312—H312	119.8
O2—C7—C8	106.0 (6)	C307—C312—H312	119.8
O2—C7—H7A	110.5	C402—C401—C406	119.9 (5)
C8—C7—H7A	110.5	C402—C401—P4	120.3 (4)
O2—C7—H7B	110.5	C406—C401—P4	119.7 (4)
C8—C7—H7B	110.5	C401—C402—C403	119.7 (5)
H7A—C7—H7B	108.7	C401—C402—H402	120.2
C7—C8—H8A	109.5	C403—C402—H402	120.2
C7—C8—H8B	109.5	C404—C403—C402	120.7 (6)
H8A—C8—H8B	109.5	C404—C403—H403	119.7
C7—C8—H8C	109.5	C402—C403—H403	119.7
H8A—C8—H8C	109.5	C403—C404—C405	119.9 (5)
H8B—C8—H8C	109.5	C403—C404—H404	120.1
C102—C101—C106	118.7 (6)	C405—C404—H404	120.1
C102—C101—P1	124.5 (5)	C404—C405—C406	120.4 (6)
C106—C101—P1	116.7 (5)	C404—C405—H405	119.8
C101—C102—C103	121.3 (8)	C406—C405—H405	119.8
C101—C102—H102	119.4	C401—C406—C405	119.5 (6)
C103—C102—H102	119.4	C401—C406—H406	120.3
C104—C103—C102	119.3 (9)	C405—C406—H406	120.3
C104—C103—H103	120.4	C412—C407—C408	118.9 (5)
C102—C103—H103	120.4	C412—C407—P4	125.1 (5)
C103—C104—C105	120.9 (7)	C408—C407—P4	116.0 (4)
C103—C104—H104	119.6	C409—C408—C407	121.2 (6)
C105—C104—H104	119.6	C409—C408—H408	119.4
C104—C105—C106	119.8 (7)	C407—C408—H408	119.4
C104—C105—H105	120.1	C410—C409—C408	119.8 (7)
C106—C105—H105	120.1	C410—C409—H409	120.1
C101—C106—C105	120.0 (7)	C408—C409—H409	120.1
C101—C106—H106	120.0	C411—C410—C409	119.5 (7)
C105—C106—H106	120.0	C411—C410—H410	120.2
C112—C107—C108	119.6 (6)	C409—C410—H410	120.2
C112—C107—P1	118.9 (5)	C410—C411—C412	121.7 (7)
C108—C107—P1	121.5 (5)	C410—C411—H411	119.2
C107—C108—C109	118.0 (8)	C412—C411—H411	119.2
C107—C108—H108	121.0	C407—C412—C411	118.9 (7)
C109—C108—H108	121.0	C407—C412—H412	120.5
C110—C109—C108	120.4 (9)	C411—C412—H412	120.5
C110—C109—H109	119.8	Cl3—C9—Cl4	118.8 (10)
C108—C109—H109	119.8	Cl6—C10—Cl5	112.1 (5)
C111—C110—C109	121.6 (8)	Cl7—C11—Cl8	110.0 (7)
C111—C110—H110	119.2	Cl8A—C11—Cl7A	94.2 (13)
C109—C110—H110	119.2	C11—Cl8—Cl9	105.0 (7)
C110—C111—C112	118.7 (9)	Cl10—C12—C12^i^	169 (4)
C110—C111—H111	120.6	Cl10—C12—Cl9	111 (4)
C112—C111—H111	120.6	C12^i^—C12—Cl9	72 (4)
C111—C112—C107	121.8 (7)	Cl10—C12—Cl9^i^	117 (4)
C111—C112—H112	119.1	C12^i^—C12—Cl9^i^	59 (4)
C107—C112—H112	119.1	Cl9—C12—Cl9^i^	131 (3)
C206—C201—C202	119.3 (5)	C12—Cl9—Cl8	130.1 (14)
C206—C201—P2	120.4 (4)	C12—Cl9—C12^i^	49 (3)
C202—C201—P2	120.2 (4)	Cl8—Cl9—C12^i^	138.4 (11)
C203—C202—C201	120.2 (6)		
			
C301—P3—C1—C4	−82.4 (4)	C201—C202—C203—C204	−0.8 (9)
C307—P3—C1—C4	156.4 (3)	C202—C203—C204—C205	1.2 (10)
C3—P3—C1—C4	39.2 (4)	C203—C204—C205—C206	−1.5 (10)
C301—P3—C1—P2	74.9 (4)	C204—C205—C206—C201	1.4 (9)
C307—P3—C1—P2	−46.3 (4)	C202—C201—C206—C205	−1.0 (8)
C3—P3—C1—P2	−163.5 (3)	P2—C201—C206—C205	−178.5 (4)
C301—P3—C1—Ir1	−151.7 (2)	C1—P2—C207—C208	−164.2 (4)
C307—P3—C1—Ir1	87.1 (3)	C201—P2—C207—C208	68.2 (5)
C3—P3—C1—Ir1	−30.1 (3)	C2—P2—C207—C208	−44.0 (5)
C207—P2—C1—C4	51.6 (4)	C1—P2—C207—C212	16.3 (5)
C201—P2—C1—C4	174.1 (3)	C201—P2—C207—C212	−111.3 (5)
C2—P2—C1—C4	−66.6 (4)	C2—P2—C207—C212	136.5 (5)
C207—P2—C1—P3	−107.3 (3)	C212—C207—C208—C209	−1.2 (9)
C201—P2—C1—P3	15.2 (4)	P2—C207—C208—C209	179.3 (5)
C2—P2—C1—P3	134.5 (3)	C207—C208—C209—C210	−0.2 (12)
C207—P2—C1—Ir1	119.1 (2)	C208—C209—C210—C211	1.5 (13)
C201—P2—C1—Ir1	−118.4 (2)	C209—C210—C211—C212	−1.4 (12)
C2—P2—C1—Ir1	0.9 (3)	C208—C207—C212—C211	1.2 (9)
C1—P2—C2—P1	28.3 (4)	P2—C207—C212—C211	−179.3 (5)
C207—P2—C2—P1	−93.0 (3)	C210—C211—C212—C207	0.1 (10)
C201—P2—C2—P1	153.9 (3)	C1—P3—C301—C306	−39.5 (5)
C107—P1—C2—P2	82.4 (3)	C307—P3—C301—C306	86.1 (5)
C101—P1—C2—P2	−169.7 (3)	C3—P3—C301—C306	−162.8 (4)
Ir1—P1—C2—P2	−42.7 (3)	C1—P3—C301—C302	146.1 (4)
C1—P3—C3—P4	28.1 (4)	C307—P3—C301—C302	−88.3 (5)
C301—P3—C3—P4	156.0 (3)	C3—P3—C301—C302	22.9 (5)
C307—P3—C3—P4	−93.7 (3)	C306—C301—C302—C303	0.4 (9)
C407—P4—C3—P3	114.6 (3)	P3—C301—C302—C303	174.9 (5)
C401—P4—C3—P3	−139.3 (3)	C301—C302—C303—C304	0.6 (11)
Ir1—P4—C3—P3	−13.1 (3)	C302—C303—C304—C305	−1.1 (12)
P3—C1—C4—C5	26.1 (6)	C303—C304—C305—C306	0.5 (11)
P2—C1—C4—C5	−133.8 (4)	C302—C301—C306—C305	−1.0 (9)
Ir1—C1—C4—C5	124.6 (5)	P3—C301—C306—C305	−175.4 (5)
P3—C1—C4—Ir1	−98.6 (3)	C304—C305—C306—C301	0.6 (10)
P2—C1—C4—Ir1	101.6 (3)	C1—P3—C307—C312	121.4 (4)
C7—O2—C5—O1	−3.8 (8)	C301—P3—C307—C312	−7.0 (5)
C7—O2—C5—C4	177.9 (5)	C3—P3—C307—C312	−118.9 (4)
C1—C4—C5—O1	−2.7 (8)	C1—P3—C307—C308	−65.1 (5)
Ir1—C4—C5—O1	94.4 (6)	C301—P3—C307—C308	166.5 (4)
C1—C4—C5—O2	175.6 (4)	C3—P3—C307—C308	54.6 (5)
Ir1—C4—C5—O2	−87.3 (5)	C312—C307—C308—C309	0.2 (8)
C5—O2—C7—C8	179.9 (6)	P3—C307—C308—C309	−173.4 (5)
C107—P1—C101—C102	100.6 (7)	C307—C308—C309—C310	0.1 (10)
C2—P1—C101—C102	−10.6 (8)	C308—C309—C310—C311	−0.3 (11)
Ir1—P1—C101—C102	−125.5 (7)	C309—C310—C311—C312	0.2 (11)
C107—P1—C101—C106	−77.3 (6)	C310—C311—C312—C307	0.1 (10)
C2—P1—C101—C106	171.5 (5)	C308—C307—C312—C311	−0.3 (8)
Ir1—P1—C101—C106	56.6 (6)	P3—C307—C312—C311	173.2 (5)
C106—C101—C102—C103	−0.9 (15)	C407—P4—C401—C402	−121.2 (5)
P1—C101—C102—C103	−178.7 (9)	C3—P4—C401—C402	129.1 (4)
C101—C102—C103—C104	1.9 (19)	Ir1—P4—C401—C402	9.5 (5)
C102—C103—C104—C105	−0.5 (18)	C407—P4—C401—C406	55.9 (5)
C103—C104—C105—C106	−1.8 (15)	C3—P4—C401—C406	−53.8 (5)
C102—C101—C106—C105	−1.4 (12)	Ir1—P4—C401—C406	−173.5 (4)
P1—C101—C106—C105	176.6 (6)	C406—C401—C402—C403	1.5 (9)
C104—C105—C106—C101	2.8 (13)	P4—C401—C402—C403	178.6 (4)
C101—P1—C107—C112	102.8 (5)	C401—C402—C403—C404	0.0 (9)
C2—P1—C107—C112	−146.0 (4)	C402—C403—C404—C405	−1.0 (10)
Ir1—P1—C107—C112	−32.0 (5)	C403—C404—C405—C406	0.5 (11)
C101—P1—C107—C108	−75.7 (5)	C402—C401—C406—C405	−2.0 (10)
C2—P1—C107—C108	35.5 (6)	P4—C401—C406—C405	−179.1 (6)
Ir1—P1—C107—C108	149.5 (4)	C404—C405—C406—C401	1.0 (11)
C112—C107—C108—C109	1.0 (10)	C401—P4—C407—C412	−94.0 (6)
P1—C107—C108—C109	179.5 (5)	C3—P4—C407—C412	12.8 (6)
C107—C108—C109—C110	−1.0 (12)	Ir1—P4—C407—C412	135.0 (5)
C108—C109—C110—C111	0.2 (15)	C401—P4—C407—C408	83.0 (4)
C109—C110—C111—C112	0.6 (14)	C3—P4—C407—C408	−170.2 (4)
C110—C111—C112—C107	−0.6 (11)	Ir1—P4—C407—C408	−48.0 (4)
C108—C107—C112—C111	−0.2 (9)	C412—C407—C408—C409	2.2 (9)
P1—C107—C112—C111	−178.7 (5)	P4—C407—C408—C409	−175.0 (5)
C1—P2—C201—C206	−105.6 (4)	C407—C408—C409—C410	−1.9 (9)
C207—P2—C201—C206	19.5 (5)	C408—C409—C410—C411	−0.7 (11)
C2—P2—C201—C206	132.5 (4)	C409—C410—C411—C412	3.0 (13)
C1—P2—C201—C202	77.0 (5)	C408—C407—C412—C411	0.1 (10)
C207—P2—C201—C202	−157.9 (4)	P4—C407—C412—C411	177.1 (6)
C2—P2—C201—C202	−44.9 (5)	C410—C411—C412—C407	−2.7 (13)
C206—C201—C202—C203	0.8 (8)	Cl7—C11—Cl8—Cl9	−171.2 (8)
P2—C201—C202—C203	178.2 (5)		

Symmetry code: (i) −*x*+2, −*y*, −*z*.

### (Bis{[(diphenylphosphanyl)methyl]diphenylphosphanylidene}(ethoxyoxoethanylidene)methane-κ4P,C,C',P')chloridohydridoiridium(III) chloride methylene chloride 2.75-solvate (4) Hydrogen-bond geometry (Å, º)

**Table:**

*D*—H···*A*	*D*—H	H···*A*	*D*···*A*	*D*—H···*A*
C2—H2*B*···Cl2	0.98	2.58	3.488 (5)	154
C3—H3*A*···O1	0.98	2.31	2.892 (7)	117
C3—H3*B*···Cl2^ii^	0.98	2.83	3.456 (5)	122

Symmetry code: (ii) *x*−1, *y*, *z*.

### (Bis{[(diphenylphosphanyl)methyl]diphenylphosphanylidene}(ethoxyoxoethanylidene)methane-κ4P,C,C',P')chlorido(ethoxyoxoethanido)iridium(III) chloride–methanol–water (1/1/0.5) (5) Crystal data

**Table:**

[Ir(C_4_H_7_O_2_)Cl(C_55_H_50_O_2_P_4_)]Cl·CH_4_O·0.5H_2_O	*Z* = 2
*M**_r_* = 1258.07	*F*(000) = 1274
Triclinic, *P*1	*D*_x_ = 1.449 Mg m^−^^3^
*a* = 12.4253 (3) Å	Mo *K*α radiation, λ = 0.71073 Å
*b* = 13.7081 (4) Å	Cell parameters from 57775 reflections
*c* = 17.6780 (6) Å	θ = 1.0–22.5°
α = 93.152 (2)°	µ = 2.57 mm^−^^1^
β = 97.960 (2)°	*T* = 233 K
γ = 103.771 (2)°	Plate, colorless
*V* = 2884.18 (15) Å^3^	0.15 × 0.05 × 0.02 mm

### (Bis{[(diphenylphosphanyl)methyl]diphenylphosphanylidene}(ethoxyoxoethanylidene)methane-κ4P,C,C',P')chlorido(ethoxyoxoethanido)iridium(III) chloride–methanol–water (1/1/0.5) (5) Data collection

**Table:**

Nonius KappaCCD diffractometer	6326 reflections with *I* > 2σ(*I*)
Radiation source: fine-focus sealed tube	*R*_int_ = 0.037
Graphite monochromator	θ_max_ = 22.4°, θ_min_ = 1.9°
phi– and ω–scans	*h* = −13→13
13821 measured reflections	*k* = −14→14
7453 independent reflections	*l* = −18→18

### (Bis{[(diphenylphosphanyl)methyl]diphenylphosphanylidene}(ethoxyoxoethanylidene)methane-κ4P,C,C',P')chlorido(ethoxyoxoethanido)iridium(III) chloride–methanol–water (1/1/0.5) (5) Refinement

**Table:**

Refinement on *F*^2^	1 restraint
Least-squares matrix: full	Hydrogen site location: mixed
*R*[*F*^2^ > 2σ(*F*^2^)] = 0.044	H atoms treated by a mixture of independent and constrained refinement
*wR*(*F*^2^) = 0.106	*w* = 1/[σ^2^(*F*_o_^2^) + (0.047*P*)^2^ + 6.9463*P*] where *P* = (*F*_o_^2^ + 2*F*_c_^2^)/3
*S* = 1.07	(Δ/σ)_max_ = 0.001
7453 reflections	Δρ_max_ = 0.90 e Å^−^^3^
674 parameters	Δρ_min_ = −0.96 e Å^−^^3^

### (Bis{[(diphenylphosphanyl)methyl]diphenylphosphanylidene}(ethoxyoxoethanylidene)methane-κ4P,C,C',P')chlorido(ethoxyoxoethanido)iridium(III) chloride–methanol–water (1/1/0.5) (5) Special details

**Table:**

Geometry. All esds (except the esd in the dihedral angle between two l.s. planes) are estimated using the full covariance matrix. The cell esds are taken into account individually in the estimation of esds in distances, angles and torsion angles; correlations between esds in cell parameters are only used when they are defined by crystal symmetry. An approximate (isotropic) treatment of cell esds is used for estimating esds involving l.s. planes.
Refinement. Small crystal with low diffraction, but good quality. Reflections were collected only until 45 degrees (2Theta). Hydrogen at C4 was found and refined isotropically with bond restraint (d=0.96 angs.). Solvent molecules methanole and water lies nearby an inversion centre and were all refined with multipicity of 0.5 (C12-O5, C13-O6 and O7). Hydrogens of these disordered molecules were not exact logalized and omitted. A 1:1 positional disorder occurs for one phenyl group of the phospane (C401-C406 and C41A-C46A). The distance of the carbon atoms between disordered rings are small and all atoms were refined isotropically.

### (Bis{[(diphenylphosphanyl)methyl]diphenylphosphanylidene}(ethoxyoxoethanylidene)methane-κ4P,C,C',P')chlorido(ethoxyoxoethanido)iridium(III) chloride–methanol–water (1/1/0.5) (5) Fractional atomic coordinates and isotropic or equivalent isotropic displacement parameters (Å^2^)

**Table:**

	*x*	*y*	*z*	*U*_iso_*/*U*_eq_	Occ. (<1)
Ir1	−0.16510 (2)	−0.10634 (2)	0.69970 (2)	0.04147 (12)	
P1	−0.15686 (15)	−0.26886 (16)	0.72460 (11)	0.0454 (5)	
P2	−0.35224 (14)	−0.24371 (15)	0.79978 (10)	0.0416 (5)	
P3	−0.35696 (14)	−0.02021 (14)	0.78288 (10)	0.0414 (5)	
P4	−0.17014 (15)	0.05959 (16)	0.68972 (11)	0.0474 (5)	
Cl1	−0.00070 (14)	−0.05642 (16)	0.80140 (11)	0.0558 (5)	
Cl2	−0.3358 (4)	0.3182 (3)	0.8216 (2)	0.1433 (14)	
C1	−0.3307 (5)	−0.1353 (5)	0.7465 (4)	0.0389 (16)	
C2	−0.2231 (6)	−0.2821 (6)	0.8122 (4)	0.0479 (19)	
H2A	−0.2378	−0.3526	0.8242	0.057*	
H2B	−0.1716	−0.2407	0.8555	0.057*	
C3	−0.2880 (6)	0.0839 (5)	0.7350 (4)	0.053 (2)	
H3A	−0.2597	0.1432	0.7722	0.063*	
H3B	−0.3433	0.0996	0.6954	0.063*	
C101	−0.0247 (6)	−0.3050 (6)	0.7536 (4)	0.053 (2)	
C102	0.0243 (7)	−0.3454 (7)	0.6989 (5)	0.073 (3)	
H102	−0.0094	−0.3522	0.6473	0.088*	
C103	0.1207 (8)	−0.3760 (8)	0.7172 (6)	0.088 (3)	
H103	0.1516	−0.4045	0.6785	0.106*	
C104	0.1718 (7)	−0.3654 (8)	0.7907 (7)	0.088 (3)	
H104	0.2376	−0.3873	0.8034	0.106*	
C105	0.1274 (8)	−0.3226 (9)	0.8466 (6)	0.091 (3)	
H105	0.1638	−0.3137	0.8977	0.110*	
C106	0.0293 (7)	−0.2922 (7)	0.8284 (5)	0.073 (3)	
H106	−0.0007	−0.2628	0.8671	0.087*	
C107	−0.2385 (6)	−0.3784 (6)	0.6601 (4)	0.0460 (19)	
C108	−0.2860 (5)	−0.3683 (6)	0.5870 (4)	0.0445 (18)	
H108	−0.2745	−0.3043	0.5684	0.053*	
C109	−0.3505 (7)	−0.4520 (7)	0.5407 (5)	0.061 (2)	
H109	−0.3840	−0.4444	0.4911	0.073*	
C110	−0.3662 (7)	−0.5465 (7)	0.5666 (5)	0.064 (2)	
H110	−0.4097	−0.6031	0.5345	0.077*	
C111	−0.3188 (8)	−0.5580 (7)	0.6388 (6)	0.069 (2)	
H111	−0.3305	−0.6224	0.6568	0.083*	
C112	−0.2531 (7)	−0.4745 (7)	0.6857 (5)	0.060 (2)	
H112	−0.2185	−0.4827	0.7349	0.072*	
C201	−0.3904 (6)	−0.2312 (5)	0.8937 (4)	0.0448 (18)	
C202	−0.5033 (6)	−0.2505 (6)	0.9013 (4)	0.053 (2)	
H202	−0.5579	−0.2658	0.8570	0.064*	
C203	−0.5363 (8)	−0.2477 (6)	0.9718 (5)	0.066 (2)	
H203	−0.6129	−0.2602	0.9758	0.079*	
C204	−0.4576 (8)	−0.2267 (7)	1.0364 (5)	0.072 (3)	
H204	−0.4804	−0.2267	1.0849	0.086*	
C205	−0.3439 (9)	−0.2053 (7)	1.0310 (5)	0.074 (3)	
H205	−0.2900	−0.1884	1.0756	0.089*	
C206	−0.3110 (7)	−0.2089 (6)	0.9600 (4)	0.060 (2)	
H206	−0.2342	−0.1963	0.9562	0.073*	
C207	−0.4620 (6)	−0.3491 (6)	0.7494 (4)	0.0446 (18)	
C208	−0.5408 (5)	−0.3389 (6)	0.6880 (4)	0.0462 (18)	
H208	−0.5374	−0.2756	0.6692	0.055*	
C209	−0.6234 (6)	−0.4213 (7)	0.6548 (5)	0.056 (2)	
H209	−0.6767	−0.4145	0.6135	0.068*	
C210	−0.6281 (7)	−0.5149 (7)	0.6825 (5)	0.070 (3)	
H210	−0.6844	−0.5713	0.6593	0.083*	
C211	−0.5522 (8)	−0.5262 (7)	0.7431 (6)	0.072 (3)	
H211	−0.5563	−0.5897	0.7618	0.086*	
C212	−0.4681 (7)	−0.4419 (6)	0.7769 (5)	0.058 (2)	
H212	−0.4154	−0.4487	0.8186	0.069*	
C301	−0.5010 (6)	−0.0138 (6)	0.7748 (4)	0.0418 (17)	
C302	−0.5909 (6)	−0.0976 (6)	0.7612 (4)	0.053 (2)	
H302	−0.5775	−0.1620	0.7548	0.064*	
C303	−0.6999 (6)	−0.0886 (7)	0.7567 (5)	0.062 (2)	
H303	−0.7600	−0.1465	0.7481	0.074*	
C304	−0.7200 (6)	0.0057 (8)	0.7648 (4)	0.060 (2)	
H304	−0.7941	0.0123	0.7614	0.072*	
C305	−0.6329 (7)	0.0893 (7)	0.7777 (4)	0.057 (2)	
H305	−0.6472	0.1534	0.7824	0.068*	
C306	−0.5232 (6)	0.0807 (6)	0.7838 (4)	0.052 (2)	
H306	−0.4636	0.1389	0.7941	0.063*	
C307	−0.3001 (6)	0.0081 (6)	0.8841 (4)	0.0466 (19)	
C308	−0.1853 (6)	0.0207 (7)	0.9072 (5)	0.071 (3)	
H308	−0.1390	0.0106	0.8712	0.085*	
C309	−0.1405 (7)	0.0481 (8)	0.9833 (5)	0.084 (3)	
H309	−0.0630	0.0574	0.9987	0.101*	
C310	−0.2066 (7)	0.0621 (7)	1.0376 (5)	0.071 (3)	
H310	−0.1753	0.0795	1.0895	0.085*	
C311	−0.3204 (6)	0.0499 (6)	1.0135 (4)	0.059 (2)	
H311	−0.3664	0.0598	1.0497	0.071*	
C312	−0.3670 (6)	0.0239 (6)	0.9383 (4)	0.0493 (19)	
H312	−0.4443	0.0167	0.9231	0.059*	
C407	−0.1952 (5)	0.1127 (6)	0.5980 (4)	0.0473 (19)	
C408	−0.2121 (6)	0.0529 (6)	0.5294 (4)	0.056 (2)	
H408	−0.2111	−0.0155	0.5295	0.067*	
C409	−0.2304 (8)	0.0951 (8)	0.4601 (5)	0.071 (2)	
H409	−0.2410	0.0547	0.4137	0.085*	
C410	−0.2332 (8)	0.1947 (8)	0.4586 (6)	0.074 (3)	
H410	−0.2464	0.2223	0.4117	0.088*	
C411	−0.2163 (7)	0.2534 (7)	0.5275 (7)	0.076 (3)	
H411	−0.2176	0.3216	0.5269	0.091*	
C412	−0.1977 (7)	0.2145 (7)	0.5966 (5)	0.064 (2)	
H412	−0.1866	0.2557	0.6427	0.076*	
O1	−0.4427 (5)	−0.0485 (4)	0.6132 (3)	0.0596 (14)	
O2	−0.4118 (4)	−0.1706 (4)	0.5351 (3)	0.0514 (13)	
O3	−0.1320 (7)	−0.1339 (6)	0.4883 (4)	0.100 (2)	
O4	−0.0633 (4)	−0.2499 (4)	0.5440 (3)	0.0587 (14)	
C4	−0.3334 (6)	−0.1604 (5)	0.6621 (4)	0.0390 (17)	
H4	−0.353 (4)	−0.2318 (16)	0.654 (3)	0.011 (13)*	
C5	−0.4024 (5)	−0.1187 (6)	0.6027 (4)	0.0411 (17)	
C6	−0.4691 (7)	−0.1344 (7)	0.4686 (4)	0.063 (2)	
H6A	−0.4417	−0.0612	0.4685	0.075*	
H6B	−0.5502	−0.1509	0.4686	0.075*	
C7	−0.4415 (9)	−0.1883 (8)	0.4004 (5)	0.088 (3)	
H7A	−0.4771	−0.1677	0.3537	0.131*	
H7B	−0.3609	−0.1714	0.4017	0.131*	
H7C	−0.4687	−0.2605	0.4018	0.131*	
C8	−0.0453 (6)	−0.1001 (6)	0.6211 (4)	0.054 (2)	
H8A	−0.0163	−0.0292	0.6126	0.064*	
H8B	0.0179	−0.1237	0.6464	0.064*	
C9	−0.0851 (7)	−0.1581 (7)	0.5455 (5)	0.059 (2)	
C10	−0.0925 (8)	−0.3085 (7)	0.4698 (5)	0.074 (3)	
H10A	−0.0483	−0.2737	0.4330	0.088*	
H10B	−0.1722	−0.3169	0.4501	0.088*	
C11	−0.0689 (8)	−0.4077 (7)	0.4797 (6)	0.093 (3)	
H11A	−0.0879	−0.4481	0.4307	0.140*	
H11B	0.0102	−0.3986	0.4989	0.140*	
H11C	−0.1134	−0.4416	0.5159	0.140*	
O5	−0.2422 (14)	0.5220 (9)	0.8989 (9)	0.107 (5)	0.5
C12	−0.190 (5)	0.564 (4)	0.9732 (15)	0.25 (3)	0.5
O6	−0.5573 (15)	0.4914 (9)	1.0173 (8)	0.101 (5)	0.5
C13	−0.665 (6)	0.4857 (18)	0.979 (2)	0.28 (4)	0.5
O7	−0.3475 (17)	0.5078 (11)	1.0734 (10)	0.113 (6)	0.5
C401	−0.0507 (14)	0.1668 (14)	0.7345 (10)	0.039 (5)*	0.5
C402	−0.0572 (18)	0.2344 (16)	0.7933 (12)	0.083 (6)*	0.5
H402	−0.1241	0.2327	0.8130	0.099*	0.5
C403	0.044 (2)	0.3058 (19)	0.8217 (15)	0.108 (7)*	0.5
H403	0.0442	0.3517	0.8633	0.129*	0.5
C404	0.137 (2)	0.3123 (17)	0.7935 (12)	0.085 (6)*	0.5
H404	0.1997	0.3663	0.8120	0.102*	0.5
C405	0.1458 (16)	0.2471 (15)	0.7416 (14)	0.055 (5)*	0.5
H405	0.2133	0.2501	0.7223	0.065*	0.5
C406	0.047 (2)	0.1700 (17)	0.7148 (14)	0.065 (8)*	0.5
H406	0.0528	0.1173	0.6803	0.077*	0.5
C41A	−0.0372 (18)	0.1384 (16)	0.7366 (12)	0.055 (7)*	0.5
C42A	−0.0192 (18)	0.1861 (15)	0.8085 (12)	0.079 (6)*	0.5
H42A	−0.0785	0.1777	0.8373	0.095*	0.5
C43A	0.093 (2)	0.2514 (18)	0.8423 (15)	0.103 (7)*	0.5
H43A	0.1097	0.2786	0.8938	0.123*	0.5
C44A	0.171 (2)	0.2690 (18)	0.7943 (14)	0.083 (6)*	0.5
H44A	0.2410	0.3123	0.8137	0.100*	0.5
C45A	0.1536 (17)	0.2269 (15)	0.7166 (12)	0.062 (6)*	0.5
H45A	0.2095	0.2422	0.6853	0.074*	0.5
C46A	0.0517 (15)	0.1634 (13)	0.6908 (12)	0.037 (5)*	0.5
H46A	0.0380	0.1339	0.6400	0.045*	0.5

### (Bis{[(diphenylphosphanyl)methyl]diphenylphosphanylidene}(ethoxyoxoethanylidene)methane-κ4P,C,C',P')chlorido(ethoxyoxoethanido)iridium(III) chloride–methanol–water (1/1/0.5) (5) Atomic displacement parameters (Å^2^)

**Table:**

	*U*^11^	*U*^22^	*U*^33^	*U*^12^	*U*^13^	*U*^23^
Ir1	0.02378 (16)	0.0610 (2)	0.03666 (18)	0.00846 (12)	0.00368 (11)	−0.00960 (13)
P1	0.0282 (10)	0.0686 (14)	0.0392 (11)	0.0161 (9)	0.0022 (8)	−0.0081 (9)
P2	0.0285 (9)	0.0612 (13)	0.0326 (10)	0.0099 (9)	0.0029 (8)	−0.0066 (9)
P3	0.0307 (10)	0.0554 (12)	0.0360 (10)	0.0092 (9)	0.0059 (8)	−0.0092 (9)
P4	0.0311 (10)	0.0633 (13)	0.0418 (11)	0.0025 (9)	0.0069 (9)	−0.0082 (9)
Cl1	0.0271 (9)	0.0823 (14)	0.0511 (12)	0.0104 (9)	−0.0032 (8)	−0.0146 (10)
Cl2	0.170 (4)	0.105 (3)	0.150 (3)	0.013 (2)	0.049 (3)	−0.001 (2)
C1	0.028 (4)	0.055 (4)	0.034 (4)	0.014 (3)	0.001 (3)	−0.004 (3)
C2	0.039 (4)	0.070 (5)	0.034 (4)	0.018 (4)	0.000 (3)	−0.009 (4)
C3	0.055 (5)	0.049 (5)	0.055 (5)	0.008 (4)	0.025 (4)	−0.005 (4)
C101	0.034 (4)	0.075 (6)	0.052 (5)	0.022 (4)	0.001 (4)	−0.007 (4)
C102	0.048 (5)	0.117 (8)	0.059 (6)	0.038 (5)	−0.002 (4)	−0.010 (5)
C103	0.058 (6)	0.124 (9)	0.088 (8)	0.048 (6)	0.001 (6)	−0.025 (6)
C104	0.046 (5)	0.113 (9)	0.115 (9)	0.044 (6)	0.001 (6)	0.003 (7)
C105	0.058 (6)	0.137 (10)	0.078 (7)	0.041 (6)	−0.018 (5)	−0.004 (7)
C106	0.052 (5)	0.110 (8)	0.059 (6)	0.036 (5)	−0.003 (5)	−0.007 (5)
C107	0.037 (4)	0.055 (5)	0.047 (5)	0.016 (4)	0.006 (4)	−0.009 (4)
C108	0.034 (4)	0.056 (5)	0.040 (5)	0.010 (4)	0.003 (3)	−0.015 (4)
C109	0.050 (5)	0.076 (7)	0.056 (5)	0.025 (5)	0.006 (4)	−0.019 (5)
C110	0.051 (5)	0.068 (7)	0.068 (6)	0.009 (4)	0.009 (5)	−0.023 (5)
C111	0.072 (6)	0.055 (6)	0.078 (7)	0.015 (5)	0.010 (5)	−0.009 (5)
C112	0.063 (5)	0.071 (6)	0.053 (5)	0.029 (5)	0.012 (4)	0.000 (5)
C201	0.045 (4)	0.058 (5)	0.030 (4)	0.012 (4)	0.004 (3)	−0.003 (3)
C202	0.044 (5)	0.075 (6)	0.042 (5)	0.016 (4)	0.008 (4)	0.000 (4)
C203	0.064 (6)	0.081 (6)	0.058 (6)	0.018 (5)	0.027 (5)	−0.006 (5)
C204	0.085 (7)	0.077 (6)	0.057 (6)	0.017 (5)	0.034 (6)	−0.006 (5)
C205	0.091 (7)	0.098 (7)	0.038 (5)	0.036 (6)	0.006 (5)	−0.003 (5)
C206	0.059 (5)	0.080 (6)	0.044 (5)	0.023 (5)	0.008 (4)	−0.004 (4)
C207	0.035 (4)	0.052 (5)	0.044 (4)	0.007 (3)	0.009 (4)	−0.011 (4)
C208	0.031 (4)	0.058 (5)	0.045 (4)	0.006 (4)	0.003 (4)	−0.013 (4)
C209	0.038 (4)	0.072 (6)	0.053 (5)	0.003 (4)	0.013 (4)	−0.012 (4)
C210	0.056 (6)	0.077 (7)	0.060 (6)	−0.011 (5)	0.012 (5)	−0.018 (5)
C211	0.071 (6)	0.063 (6)	0.079 (7)	0.007 (5)	0.020 (6)	0.004 (5)
C212	0.056 (5)	0.056 (6)	0.057 (5)	0.005 (4)	0.008 (4)	0.008 (4)
C301	0.035 (4)	0.058 (5)	0.034 (4)	0.016 (4)	0.006 (3)	−0.002 (3)
C302	0.039 (4)	0.065 (5)	0.056 (5)	0.013 (4)	0.011 (4)	−0.008 (4)
C303	0.028 (4)	0.089 (7)	0.058 (5)	0.001 (4)	0.001 (4)	−0.006 (5)
C304	0.032 (4)	0.106 (7)	0.048 (5)	0.027 (5)	0.008 (4)	0.000 (5)
C305	0.050 (5)	0.080 (6)	0.050 (5)	0.034 (5)	0.010 (4)	0.001 (4)
C306	0.047 (5)	0.071 (6)	0.039 (4)	0.017 (4)	0.005 (4)	−0.005 (4)
C307	0.036 (4)	0.064 (5)	0.037 (4)	0.010 (4)	0.007 (3)	−0.011 (4)
C308	0.036 (5)	0.110 (7)	0.060 (6)	0.020 (5)	−0.003 (4)	−0.031 (5)
C309	0.041 (5)	0.136 (9)	0.062 (6)	0.025 (5)	−0.017 (5)	−0.045 (6)
C310	0.056 (5)	0.108 (7)	0.045 (5)	0.027 (5)	−0.005 (4)	−0.028 (5)
C311	0.045 (5)	0.089 (6)	0.042 (5)	0.019 (4)	0.006 (4)	−0.012 (4)
C312	0.034 (4)	0.073 (5)	0.038 (4)	0.014 (4)	0.003 (3)	−0.011 (4)
C407	0.023 (4)	0.061 (5)	0.057 (5)	0.006 (3)	0.010 (3)	0.002 (4)
C408	0.051 (5)	0.070 (6)	0.046 (5)	0.012 (4)	0.011 (4)	0.000 (4)
C409	0.074 (6)	0.085 (7)	0.055 (6)	0.019 (5)	0.019 (5)	0.002 (5)
C410	0.070 (6)	0.092 (8)	0.066 (6)	0.026 (6)	0.019 (5)	0.022 (6)
C411	0.060 (6)	0.069 (6)	0.106 (9)	0.022 (5)	0.018 (6)	0.022 (6)
C412	0.053 (5)	0.070 (6)	0.066 (6)	0.019 (4)	0.002 (4)	−0.008 (5)
O1	0.066 (4)	0.072 (4)	0.054 (3)	0.038 (3)	0.015 (3)	0.004 (3)
O2	0.055 (3)	0.064 (3)	0.035 (3)	0.022 (3)	−0.004 (2)	−0.006 (2)
O3	0.144 (7)	0.126 (6)	0.052 (4)	0.081 (6)	0.010 (4)	−0.003 (4)
O4	0.052 (3)	0.072 (4)	0.051 (3)	0.016 (3)	0.009 (3)	−0.008 (3)
C4	0.038 (4)	0.045 (5)	0.034 (4)	0.010 (3)	0.012 (3)	−0.008 (3)
C5	0.029 (4)	0.058 (5)	0.038 (4)	0.011 (4)	0.007 (3)	0.002 (4)
C6	0.050 (5)	0.097 (7)	0.042 (5)	0.023 (5)	−0.002 (4)	0.016 (4)
C7	0.101 (8)	0.117 (8)	0.039 (5)	0.022 (7)	0.001 (5)	0.008 (5)
C8	0.039 (4)	0.066 (5)	0.054 (5)	0.012 (4)	0.008 (4)	−0.017 (4)
C9	0.044 (5)	0.076 (6)	0.059 (6)	0.019 (4)	0.014 (4)	−0.007 (5)
C10	0.073 (6)	0.088 (7)	0.057 (6)	0.019 (5)	0.016 (5)	−0.024 (5)
C11	0.080 (7)	0.079 (7)	0.116 (9)	0.011 (6)	0.029 (6)	−0.037 (6)
O5	0.156 (15)	0.052 (8)	0.109 (12)	0.022 (9)	0.004 (10)	0.027 (8)
C12	0.49 (8)	0.31 (5)	0.030 (15)	0.29 (6)	−0.03 (3)	0.00 (2)
O6	0.182 (18)	0.053 (8)	0.064 (10)	0.025 (10)	0.004 (10)	0.029 (7)
C13	0.59 (10)	0.038 (13)	0.09 (2)	−0.02 (3)	−0.21 (4)	0.016 (15)
O7	0.164 (16)	0.067 (10)	0.078 (11)	−0.018 (10)	0.002 (11)	−0.006 (9)

### (Bis{[(diphenylphosphanyl)methyl]diphenylphosphanylidene}(ethoxyoxoethanylidene)methane-κ4P,C,C',P')chlorido(ethoxyoxoethanido)iridium(III) chloride–methanol–water (1/1/0.5) (5) Geometric parameters (Å, º)

**Table:**

Ir1—C4	2.046 (7)	C304—H304	0.9400
Ir1—C8	2.163 (7)	C305—C306	1.385 (10)
Ir1—C1	2.279 (6)	C305—H305	0.9400
Ir1—P4	2.306 (2)	C306—H306	0.9400
Ir1—P1	2.318 (2)	C307—C312	1.392 (9)
Ir1—Cl1	2.4607 (18)	C307—C308	1.395 (10)
P1—C107	1.831 (7)	C308—C309	1.378 (11)
P1—C101	1.840 (7)	C308—H308	0.9400
P1—C2	1.850 (7)	C309—C310	1.380 (11)
P2—C1	1.788 (7)	C309—H309	0.9400
P2—C2	1.794 (7)	C310—C311	1.385 (11)
P2—C201	1.799 (7)	C310—H310	0.9400
P2—C207	1.823 (7)	C311—C312	1.366 (10)
P3—C1	1.789 (7)	C311—H311	0.9400
P3—C301	1.799 (7)	C312—H312	0.9400
P3—C3	1.800 (7)	C407—C408	1.388 (10)
P3—C307	1.816 (7)	C407—C412	1.404 (11)
P4—C41A	1.80 (2)	C408—C409	1.396 (11)
P4—C407	1.832 (8)	C408—H408	0.9400
P4—C3	1.850 (7)	C409—C410	1.375 (12)
P4—C401	1.871 (18)	C409—H409	0.9400
C1—C4	1.507 (9)	C410—C411	1.385 (13)
C2—H2A	0.9800	C410—H410	0.9400
C2—H2B	0.9800	C411—C412	1.372 (12)
C3—H3A	0.9800	C411—H411	0.9400
C3—H3B	0.9800	C412—H412	0.9400
C101—C102	1.371 (10)	O1—C5	1.203 (8)
C101—C106	1.378 (11)	O2—C5	1.332 (8)
C102—C103	1.364 (11)	O2—C6	1.461 (8)
C102—H102	0.9400	O3—C9	1.199 (10)
C103—C104	1.350 (13)	O4—C9	1.348 (10)
C103—H103	0.9400	O4—C10	1.453 (9)
C104—C105	1.366 (13)	C4—C5	1.488 (10)
C104—H104	0.9400	C4—H4	0.947 (19)
C105—C106	1.382 (12)	C6—C7	1.500 (11)
C105—H105	0.9400	C6—H6A	0.9800
C106—H106	0.9400	C6—H6B	0.9800
C107—C108	1.373 (10)	C7—H7A	0.9700
C107—C112	1.395 (11)	C7—H7B	0.9700
C108—C109	1.380 (10)	C7—H7C	0.9700
C108—H108	0.9400	C8—C9	1.475 (11)
C109—C110	1.377 (12)	C8—H8A	0.9800
C109—H109	0.9400	C8—H8B	0.9800
C110—C111	1.363 (12)	C10—C11	1.472 (13)
C110—H110	0.9400	C10—H10A	0.9800
C111—C112	1.388 (12)	C10—H10B	0.9800
C111—H111	0.9400	C11—H11A	0.9700
C112—H112	0.9400	C11—H11B	0.9700
C201—C202	1.391 (10)	C11—H11C	0.9700
C201—C206	1.393 (10)	O5—C12	1.41 (3)
C202—C203	1.366 (10)	O6—C13	1.39 (6)
C202—H202	0.9400	O6—O6^i^	1.60 (3)
C203—C204	1.366 (12)	C13—O7^i^	0.96 (5)
C203—H203	0.9400	O7—C13^i^	0.96 (5)
C204—C205	1.390 (12)	C401—C406	1.30 (3)
C204—H204	0.9400	C401—C402	1.38 (3)
C205—C206	1.375 (11)	C402—C403	1.40 (3)
C205—H205	0.9400	C402—H402	0.9400
C206—H206	0.9400	C403—C404	1.32 (3)
C207—C212	1.376 (10)	C403—H403	0.9400
C207—C208	1.394 (10)	C404—C405	1.28 (3)
C208—C209	1.371 (10)	C404—H404	0.9400
C208—H208	0.9400	C405—C406	1.42 (3)
C209—C210	1.389 (12)	C405—H405	0.9400
C209—H209	0.9400	C406—H406	0.9400
C210—C211	1.368 (12)	C41A—C42A	1.36 (3)
C210—H210	0.9400	C41A—C46A	1.45 (3)
C211—C212	1.400 (12)	C42A—C43A	1.49 (3)
C211—H211	0.9400	C42A—H42A	0.9400
C212—H212	0.9400	C43A—C44A	1.36 (3)
C301—C302	1.382 (10)	C43A—H43A	0.9400
C301—C306	1.393 (10)	C44A—C45A	1.43 (3)
C302—C303	1.381 (10)	C44A—H44A	0.9400
C302—H302	0.9400	C45A—C46A	1.36 (3)
C303—C304	1.378 (11)	C45A—H45A	0.9400
C303—H303	0.9400	C46A—H46A	0.9400
C304—C305	1.359 (11)		
			
C4—Ir1—C8	120.8 (3)	C306—C301—P3	118.3 (6)
C4—Ir1—C1	40.3 (2)	C303—C302—C301	121.4 (8)
C8—Ir1—C1	161.1 (3)	C303—C302—H302	119.3
C4—Ir1—P4	93.6 (2)	C301—C302—H302	119.3
C8—Ir1—P4	93.0 (2)	C304—C303—C302	119.6 (8)
C1—Ir1—P4	89.61 (18)	C304—C303—H303	120.2
C4—Ir1—P1	88.5 (2)	C302—C303—H303	120.2
C8—Ir1—P1	91.2 (2)	C305—C304—C303	120.1 (7)
C1—Ir1—P1	88.02 (18)	C305—C304—H304	120.0
P4—Ir1—P1	173.53 (7)	C303—C304—H304	120.0
C4—Ir1—Cl1	152.49 (19)	C304—C305—C306	120.7 (8)
C8—Ir1—Cl1	85.9 (2)	C304—C305—H305	119.7
C1—Ir1—Cl1	112.78 (17)	C306—C305—H305	119.7
P4—Ir1—Cl1	91.38 (7)	C305—C306—C301	120.2 (8)
P1—Ir1—Cl1	84.00 (7)	C305—C306—H306	119.9
C107—P1—C101	101.9 (3)	C301—C306—H306	119.9
C107—P1—C2	103.5 (3)	C312—C307—C308	119.4 (7)
C101—P1—C2	102.5 (3)	C312—C307—P3	121.5 (5)
C107—P1—Ir1	121.5 (3)	C308—C307—P3	118.9 (5)
C101—P1—Ir1	123.4 (3)	C309—C308—C307	119.2 (7)
C2—P1—Ir1	100.7 (3)	C309—C308—H308	120.4
C1—P2—C2	107.0 (3)	C307—C308—H308	120.4
C1—P2—C201	118.4 (3)	C308—C309—C310	121.7 (8)
C2—P2—C201	106.6 (3)	C308—C309—H309	119.2
C1—P2—C207	112.4 (3)	C310—C309—H309	119.2
C2—P2—C207	108.0 (3)	C309—C310—C311	118.3 (7)
C201—P2—C207	104.0 (3)	C309—C310—H310	120.9
C1—P3—C301	117.2 (3)	C311—C310—H310	120.9
C1—P3—C3	110.5 (3)	C312—C311—C310	121.4 (7)
C301—P3—C3	105.7 (4)	C312—C311—H311	119.3
C1—P3—C307	110.7 (3)	C310—C311—H311	119.3
C301—P3—C307	105.7 (3)	C311—C312—C307	120.0 (7)
C3—P3—C307	106.5 (4)	C311—C312—H312	120.0
C41A—P4—C407	104.1 (7)	C307—C312—H312	120.0
C41A—P4—C3	111.6 (7)	C408—C407—C412	119.5 (7)
C407—P4—C3	100.5 (3)	C408—C407—P4	120.4 (6)
C407—P4—C401	96.0 (6)	C412—C407—P4	120.1 (6)
C3—P4—C401	102.3 (6)	C407—C408—C409	119.4 (8)
C41A—P4—Ir1	108.0 (7)	C407—C408—H408	120.3
C407—P4—Ir1	123.5 (3)	C409—C408—H408	120.3
C3—P4—Ir1	108.9 (2)	C410—C409—C408	121.2 (8)
C401—P4—Ir1	122.0 (6)	C410—C409—H409	119.4
C4—C1—P2	113.5 (5)	C408—C409—H409	119.4
C4—C1—P3	121.9 (5)	C409—C410—C411	118.6 (9)
P2—C1—P3	120.6 (4)	C409—C410—H410	120.7
C4—C1—Ir1	61.5 (3)	C411—C410—H410	120.7
P2—C1—Ir1	112.4 (3)	C412—C411—C410	121.7 (9)
P3—C1—Ir1	111.3 (3)	C412—C411—H411	119.2
P2—C2—P1	111.0 (4)	C410—C411—H411	119.2
P2—C2—H2A	109.4	C411—C412—C407	119.5 (8)
P1—C2—H2A	109.4	C411—C412—H412	120.2
P2—C2—H2B	109.4	C407—C412—H412	120.2
P1—C2—H2B	109.4	C5—O2—C6	116.8 (6)
H2A—C2—H2B	108.0	C9—O4—C10	115.6 (7)
P3—C3—P4	113.8 (4)	C5—C4—C1	121.8 (6)
P3—C3—H3A	108.8	C5—C4—Ir1	126.1 (5)
P4—C3—H3A	108.8	C1—C4—Ir1	78.2 (4)
P3—C3—H3B	108.8	C5—C4—H4	110 (3)
P4—C3—H3B	108.8	C1—C4—H4	106 (3)
H3A—C3—H3B	107.7	Ir1—C4—H4	111 (3)
C102—C101—C106	117.6 (7)	O1—C5—O2	124.9 (6)
C102—C101—P1	119.2 (6)	O1—C5—C4	125.8 (6)
C106—C101—P1	123.2 (6)	O2—C5—C4	109.4 (6)
C103—C102—C101	121.9 (9)	O2—C6—C7	104.9 (6)
C103—C102—H102	119.1	O2—C6—H6A	110.8
C101—C102—H102	119.1	C7—C6—H6A	110.8
C104—C103—C102	120.2 (9)	O2—C6—H6B	110.8
C104—C103—H103	119.9	C7—C6—H6B	110.8
C102—C103—H103	119.9	H6A—C6—H6B	108.8
C103—C104—C105	119.7 (8)	C6—C7—H7A	109.5
C103—C104—H104	120.2	C6—C7—H7B	109.5
C105—C104—H104	120.2	H7A—C7—H7B	109.5
C104—C105—C106	120.3 (9)	C6—C7—H7C	109.5
C104—C105—H105	119.9	H7A—C7—H7C	109.5
C106—C105—H105	119.9	H7B—C7—H7C	109.5
C101—C106—C105	120.3 (8)	C9—C8—Ir1	117.5 (5)
C101—C106—H106	119.8	C9—C8—H8A	107.9
C105—C106—H106	119.8	Ir1—C8—H8A	107.9
C108—C107—C112	119.1 (7)	C9—C8—H8B	107.9
C108—C107—P1	121.5 (6)	Ir1—C8—H8B	107.9
C112—C107—P1	119.4 (6)	H8A—C8—H8B	107.2
C107—C108—C109	120.1 (8)	O3—C9—O4	118.8 (8)
C107—C108—H108	119.9	O3—C9—C8	128.8 (9)
C109—C108—H108	119.9	O4—C9—C8	112.4 (8)
C110—C109—C108	120.6 (8)	O4—C10—C11	108.2 (8)
C110—C109—H109	119.7	O4—C10—H10A	110.1
C108—C109—H109	119.7	C11—C10—H10A	110.1
C111—C110—C109	120.0 (8)	O4—C10—H10B	110.1
C111—C110—H110	120.0	C11—C10—H10B	110.1
C109—C110—H110	120.0	H10A—C10—H10B	108.4
C110—C111—C112	119.9 (9)	C10—C11—H11A	109.5
C110—C111—H111	120.0	C10—C11—H11B	109.5
C112—C111—H111	120.0	H11A—C11—H11B	109.5
C111—C112—C107	120.1 (8)	C10—C11—H11C	109.5
C111—C112—H112	119.9	H11A—C11—H11C	109.5
C107—C112—H112	119.9	H11B—C11—H11C	109.5
C202—C201—C206	118.4 (7)	C13—O6—O6^i^	128 (3)
C202—C201—P2	119.3 (5)	O7^i^—C13—O6	103 (7)
C206—C201—P2	122.2 (6)	C406—C401—C402	118 (2)
C203—C202—C201	121.3 (8)	C406—C401—P4	117.2 (16)
C203—C202—H202	119.4	C402—C401—P4	123.9 (14)
C201—C202—H202	119.4	C401—C402—C403	115 (2)
C204—C203—C202	119.8 (8)	C401—C402—H402	122.5
C204—C203—H203	120.1	C403—C402—H402	122.5
C202—C203—H203	120.1	C404—C403—C402	124 (2)
C203—C204—C205	120.5 (8)	C404—C403—H403	118.1
C203—C204—H204	119.7	C402—C403—H403	118.1
C205—C204—H204	119.7	C405—C404—C403	122 (2)
C206—C205—C204	119.5 (9)	C405—C404—H404	119.2
C206—C205—H205	120.2	C403—C404—H404	119.2
C204—C205—H205	120.2	C404—C405—C406	116 (2)
C205—C206—C201	120.5 (8)	C404—C405—H405	122.1
C205—C206—H206	119.8	C406—C405—H405	122.1
C201—C206—H206	119.8	C401—C406—C405	125 (2)
C212—C207—C208	119.7 (7)	C401—C406—H406	117.7
C212—C207—P2	116.7 (6)	C405—C406—H406	117.7
C208—C207—P2	123.5 (6)	C42A—C41A—C46A	118 (2)
C209—C208—C207	120.1 (8)	C42A—C41A—P4	124.6 (17)
C209—C208—H208	120.0	C46A—C41A—P4	117.3 (15)
C207—C208—H208	120.0	C41A—C42A—C43A	121 (2)
C208—C209—C210	119.8 (8)	C41A—C42A—H42A	119.5
C208—C209—H209	120.1	C43A—C42A—H42A	119.5
C210—C209—H209	120.1	C44A—C43A—C42A	116 (2)
C211—C210—C209	121.0 (8)	C44A—C43A—H43A	122.1
C211—C210—H210	119.5	C42A—C43A—H43A	122.1
C209—C210—H210	119.5	C43A—C44A—C45A	125 (2)
C210—C211—C212	119.0 (9)	C43A—C44A—H44A	117.4
C210—C211—H211	120.5	C45A—C44A—H44A	117.4
C212—C211—H211	120.5	C46A—C45A—C44A	116 (2)
C207—C212—C211	120.4 (8)	C46A—C45A—H45A	122.1
C207—C212—H212	119.8	C44A—C45A—H45A	122.1
C211—C212—H212	119.8	C45A—C46A—C41A	124 (2)
C302—C301—C306	118.1 (6)	C45A—C46A—H46A	118.0
C302—C301—P3	123.6 (5)	C41A—C46A—H46A	118.0
			
C2—P2—C1—C4	77.0 (5)	C307—P3—C301—C302	−106.7 (6)
C201—P2—C1—C4	−162.8 (5)	C1—P3—C301—C306	−163.7 (5)
C207—P2—C1—C4	−41.4 (5)	C3—P3—C301—C306	−40.2 (6)
C2—P2—C1—P3	−124.9 (4)	C307—P3—C301—C306	72.5 (6)
C201—P2—C1—P3	−4.7 (5)	C306—C301—C302—C303	−0.2 (11)
C207—P2—C1—P3	116.6 (4)	P3—C301—C302—C303	179.0 (6)
C2—P2—C1—Ir1	9.6 (4)	C301—C302—C303—C304	1.0 (12)
C201—P2—C1—Ir1	129.8 (3)	C302—C303—C304—C305	−0.5 (12)
C207—P2—C1—Ir1	−108.9 (3)	C303—C304—C305—C306	−0.9 (12)
C301—P3—C1—C4	79.1 (6)	C304—C305—C306—C301	1.7 (11)
C3—P3—C1—C4	−42.0 (6)	C302—C301—C306—C305	−1.2 (10)
C307—P3—C1—C4	−159.7 (5)	P3—C301—C306—C305	179.6 (6)
C301—P3—C1—P2	−77.1 (5)	C1—P3—C307—C312	−124.5 (6)
C3—P3—C1—P2	161.8 (4)	C301—P3—C307—C312	3.4 (7)
C307—P3—C1—P2	44.1 (5)	C3—P3—C307—C312	115.5 (7)
C301—P3—C1—Ir1	147.9 (3)	C1—P3—C307—C308	59.7 (8)
C3—P3—C1—Ir1	26.9 (4)	C301—P3—C307—C308	−172.4 (7)
C307—P3—C1—Ir1	−90.8 (4)	C3—P3—C307—C308	−60.4 (8)
C1—P2—C2—P1	−39.1 (5)	C312—C307—C308—C309	0.3 (14)
C201—P2—C2—P1	−166.6 (4)	P3—C307—C308—C309	176.2 (8)
C207—P2—C2—P1	82.2 (5)	C307—C308—C309—C310	0.8 (16)
C107—P1—C2—P2	−77.5 (5)	C308—C309—C310—C311	−1.2 (16)
C101—P1—C2—P2	176.8 (4)	C309—C310—C311—C312	0.5 (14)
Ir1—P1—C2—P2	48.8 (4)	C310—C311—C312—C307	0.6 (13)
C1—P3—C3—P4	−21.3 (6)	C308—C307—C312—C311	−1.0 (12)
C301—P3—C3—P4	−149.0 (4)	P3—C307—C312—C311	−176.8 (6)
C307—P3—C3—P4	98.9 (4)	C41A—P4—C407—C408	123.0 (9)
C41A—P4—C3—P3	−112.8 (8)	C3—P4—C407—C408	−121.4 (6)
C407—P4—C3—P3	137.3 (4)	C401—P4—C407—C408	134.9 (8)
C401—P4—C3—P3	−124.2 (7)	Ir1—P4—C407—C408	−0.3 (7)
Ir1—P4—C3—P3	6.2 (5)	C41A—P4—C407—C412	−57.1 (9)
C107—P1—C101—C102	45.5 (8)	C3—P4—C407—C412	58.5 (6)
C2—P1—C101—C102	152.5 (7)	C401—P4—C407—C412	−45.2 (8)
Ir1—P1—C101—C102	−95.6 (7)	Ir1—P4—C407—C412	179.6 (5)
C107—P1—C101—C106	−135.4 (8)	C412—C407—C408—C409	0.4 (11)
C2—P1—C101—C106	−28.4 (9)	P4—C407—C408—C409	−179.6 (6)
Ir1—P1—C101—C106	83.6 (8)	C407—C408—C409—C410	−0.6 (12)
C106—C101—C102—C103	2.6 (15)	C408—C409—C410—C411	0.6 (13)
P1—C101—C102—C103	−178.3 (8)	C409—C410—C411—C412	−0.4 (14)
C101—C102—C103—C104	−1.2 (17)	C410—C411—C412—C407	0.2 (13)
C102—C103—C104—C105	−0.9 (18)	C408—C407—C412—C411	−0.2 (11)
C103—C104—C105—C106	1.4 (17)	P4—C407—C412—C411	179.9 (6)
C102—C101—C106—C105	−2.0 (15)	P2—C1—C4—C5	130.9 (6)
P1—C101—C106—C105	178.9 (8)	P3—C1—C4—C5	−26.8 (9)
C104—C105—C106—C101	0.0 (17)	Ir1—C1—C4—C5	−125.4 (7)
C101—P1—C107—C108	−126.6 (6)	P2—C1—C4—Ir1	−103.7 (4)
C2—P1—C107—C108	127.3 (6)	P3—C1—C4—Ir1	98.5 (5)
Ir1—P1—C107—C108	15.5 (7)	C6—O2—C5—O1	4.3 (10)
C101—P1—C107—C112	53.6 (6)	C6—O2—C5—C4	−175.0 (6)
C2—P1—C107—C112	−52.5 (6)	C1—C4—C5—O1	15.8 (11)
Ir1—P1—C107—C112	−164.3 (5)	Ir1—C4—C5—O1	−83.0 (8)
C112—C107—C108—C109	2.2 (10)	C1—C4—C5—O2	−164.9 (6)
P1—C107—C108—C109	−177.6 (5)	Ir1—C4—C5—O2	96.2 (6)
C107—C108—C109—C110	−1.3 (11)	C5—O2—C6—C7	165.9 (7)
C108—C109—C110—C111	0.7 (12)	C10—O4—C9—O3	4.3 (11)
C109—C110—C111—C112	−1.0 (12)	C10—O4—C9—C8	−175.6 (7)
C110—C111—C112—C107	2.0 (12)	Ir1—C8—C9—O3	84.8 (11)
C108—C107—C112—C111	−2.6 (11)	Ir1—C8—C9—O4	−95.3 (7)
P1—C107—C112—C111	177.2 (6)	C9—O4—C10—C11	−177.0 (7)
C1—P2—C201—C202	90.5 (7)	O6^i^—O6—C13—O7^i^	2 (4)
C2—P2—C201—C202	−149.0 (6)	C407—P4—C401—C406	−79.1 (17)
C207—P2—C201—C202	−35.1 (7)	C3—P4—C401—C406	178.8 (16)
C1—P2—C201—C206	−93.7 (7)	Ir1—P4—C401—C406	57.0 (18)
C2—P2—C201—C206	26.8 (8)	C407—P4—C401—C402	109.3 (17)
C207—P2—C201—C206	140.8 (7)	C3—P4—C401—C402	7.2 (19)
C206—C201—C202—C203	0.1 (12)	Ir1—P4—C401—C402	−114.6 (17)
P2—C201—C202—C203	176.1 (6)	C406—C401—C402—C403	5 (3)
C201—C202—C203—C204	−0.7 (13)	P4—C401—C402—C403	176.6 (16)
C202—C203—C204—C205	1.8 (14)	C401—C402—C403—C404	3 (4)
C203—C204—C205—C206	−2.4 (14)	C402—C403—C404—C405	−6 (4)
C204—C205—C206—C201	1.8 (13)	C403—C404—C405—C406	2 (4)
C202—C201—C206—C205	−0.7 (12)	C402—C401—C406—C405	−10 (3)
P2—C201—C206—C205	−176.5 (7)	P4—C401—C406—C405	178.3 (17)
C1—P2—C207—C212	166.0 (5)	C404—C405—C406—C401	6 (3)
C2—P2—C207—C212	48.1 (6)	C407—P4—C41A—C42A	126.6 (19)
C201—P2—C207—C212	−64.8 (6)	C3—P4—C41A—C42A	19 (2)
C1—P2—C207—C208	−16.9 (7)	Ir1—P4—C41A—C42A	−101 (2)
C2—P2—C207—C208	−134.7 (6)	C407—P4—C41A—C46A	−44.6 (16)
C201—P2—C207—C208	112.4 (6)	C3—P4—C41A—C46A	−152.1 (13)
C212—C207—C208—C209	−0.5 (10)	Ir1—P4—C41A—C46A	88.2 (15)
P2—C207—C208—C209	−177.6 (5)	C46A—C41A—C42A—C43A	−8 (3)
C207—C208—C209—C210	−0.2 (10)	P4—C41A—C42A—C43A	−179.0 (17)
C208—C209—C210—C211	0.7 (12)	C41A—C42A—C43A—C44A	7 (3)
C209—C210—C211—C212	−0.6 (13)	C42A—C43A—C44A—C45A	−3 (4)
C208—C207—C212—C211	0.6 (11)	C43A—C44A—C45A—C46A	−1 (3)
P2—C207—C212—C211	177.9 (6)	C44A—C45A—C46A—C41A	1 (3)
C210—C211—C212—C207	−0.1 (12)	C42A—C41A—C46A—C45A	4 (3)
C1—P3—C301—C302	17.1 (7)	P4—C41A—C46A—C45A	175.7 (16)
C3—P3—C301—C302	140.7 (6)		

Symmetry code: (i) −*x*−1, −*y*+1, −*z*+2.

### (Bis{[(diphenylphosphanyl)methyl]diphenylphosphanylidene}(ethoxyoxoethanylidene)methane-κ4P,C,C',P')chlorido(ethoxyoxoethanido)iridium(III) chloride–methanol–water (1/1/0.5) (5) Hydrogen-bond geometry (Å, º)

**Table:**

*D*—H···*A*	*D*—H	H···*A*	*D*···*A*	*D*—H···*A*
C2—H2*A*···O5^ii^	0.98	2.22	3.139 (15)	156
C3—H3*A*···Cl2	0.98	2.91	3.693 (8)	137
C3—H3*B*···O1	0.98	2.40	2.895 (10)	111
C102—H102···O4	0.94	2.48	3.263 (11)	141
C212—H212···O5^ii^	0.94	2.54	3.445 (18)	163
C306—H306···Cl2	0.94	2.57	3.491 (9)	167
C308—H308···Cl1	0.94	2.56	3.464 (8)	162
C408—H408···O3	0.94	2.23	3.046 (10)	145

Symmetry code: (ii) *x*, *y*−1, *z*.

### (Bis{[(diphenylphosphanyl)methyl]diphenylphosphanylidene}(ethoxyoxoethanylidene)methane-κ4P,C,C',P')dichloridoiridium(III) chloride–methanol–water (1/1/2) (6) Crystal data

**Table:**

[IrCl_2_(C_55_H_50_O_2_P_4_)]Cl·CH_4_O·2H_2_O	*Z* = 2
*M**_r_* = 1233.45	*F*(000) = 1244
Triclinic, *P*1	*D*_x_ = 1.512 Mg m^−^^3^
*a* = 11.2371 (2) Å	Mo *K*α radiation, λ = 0.71073 Å
*b* = 12.9144 (2) Å	Cell parameters from 58878 reflections
*c* = 19.2371 (3) Å	θ = 1.0–25.3°
α = 89.439 (1)°	µ = 2.78 mm^−^^1^
β = 77.863 (1)°	*T* = 233 K
γ = 83.114 (1)°	Prism, light yellow
*V* = 2709.27 (8) Å^3^	0.11 × 0.05 × 0.03 mm

### (Bis{[(diphenylphosphanyl)methyl]diphenylphosphanylidene}(ethoxyoxoethanylidene)methane-κ4P,C,C',P')dichloridoiridium(III) chloride–methanol–water (1/1/2) (6) Data collection

**Table:**

Nonius KappaCCD diffractometer	8083 reflections with *I* > 2σ(*I*)
Radiation source: fine-focus sealed tube	*R*_int_ = 0.035
Graphite monochromator	θ_max_ = 25.0°, θ_min_ = 1.9°
phi– and ω–scans	*h* = −13→13
17984 measured reflections	*k* = −15→15
9526 independent reflections	*l* = −22→22

### (Bis{[(diphenylphosphanyl)methyl]diphenylphosphanylidene}(ethoxyoxoethanylidene)methane-κ4P,C,C',P')dichloridoiridium(III) chloride–methanol–water (1/1/2) (6) Refinement

**Table:**

Refinement on *F*^2^	1 restraint
Least-squares matrix: full	Hydrogen site location: mixed
*R*[*F*^2^ > 2σ(*F*^2^)] = 0.034	H atoms treated by a mixture of independent and constrained refinement
*wR*(*F*^2^) = 0.073	*w* = 1/[σ^2^(*F*_o_^2^) + (0.0253*P*)^2^ + 3.0412*P*] where *P* = (*F*_o_^2^ + 2*F*_c_^2^)/3
*S* = 1.05	(Δ/σ)_max_ = 0.002
9526 reflections	Δρ_max_ = 0.75 e Å^−^^3^
626 parameters	Δρ_min_ = −1.01 e Å^−^^3^

### (Bis{[(diphenylphosphanyl)methyl]diphenylphosphanylidene}(ethoxyoxoethanylidene)methane-κ4P,C,C',P')dichloridoiridium(III) chloride–methanol–water (1/1/2) (6) Special details

**Table:**

Geometry. All esds (except the esd in the dihedral angle between two l.s. planes) are estimated using the full covariance matrix. The cell esds are taken into account individually in the estimation of esds in distances, angles and torsion angles; correlations between esds in cell parameters are only used when they are defined by crystal symmetry. An approximate (isotropic) treatment of cell esds is used for estimating esds involving l.s. planes.
Refinement. Hydrogen atom at C4 found and refined isotropically with bond restraint (d = 96 pm). Hydrogens at solvent water and methanol could not be exact localized and were omitted.

### (Bis{[(diphenylphosphanyl)methyl]diphenylphosphanylidene}(ethoxyoxoethanylidene)methane-κ4P,C,C',P')dichloridoiridium(III) chloride–methanol–water (1/1/2) (6) Fractional atomic coordinates and isotropic or equivalent isotropic displacement parameters (Å^2^)

**Table:**

	*x*	*y*	*z*	*U*_iso_*/*U*_eq_	
Ir1	0.20012 (2)	0.37531 (2)	0.74968 (2)	0.02861 (6)	
P1	0.20027 (9)	0.20029 (8)	0.72614 (6)	0.0298 (2)	
P2	0.46847 (9)	0.22736 (8)	0.70579 (6)	0.0308 (2)	
P3	0.46936 (10)	0.46722 (9)	0.73724 (6)	0.0317 (2)	
P4	0.20277 (10)	0.55291 (8)	0.76838 (6)	0.0324 (2)	
Cl1	−0.01947 (9)	0.39399 (9)	0.79680 (6)	0.0416 (3)	
Cl2	0.16469 (10)	0.43456 (9)	0.63301 (5)	0.0412 (3)	
Cl3	0.44061 (18)	0.83094 (12)	0.72218 (9)	0.0876 (5)	
O1	0.3698 (3)	0.1714 (3)	0.85456 (16)	0.0483 (8)	
O2	0.2473 (3)	0.2825 (2)	0.93555 (14)	0.0423 (7)	
C1	0.3924 (4)	0.3490 (3)	0.7501 (2)	0.0316 (9)	
C2	0.3590 (4)	0.1363 (3)	0.7100 (2)	0.0347 (10)	
H2A	0.3676	0.0870	0.7482	0.042*	
H2B	0.3767	0.0966	0.6651	0.042*	
C3	0.3579 (4)	0.5712 (3)	0.7202 (2)	0.0351 (10)	
H3A	0.3597	0.5738	0.6691	0.042*	
H3B	0.3792	0.6379	0.7348	0.042*	
C4	0.3107 (4)	0.3481 (3)	0.8234 (2)	0.0306 (9)	
H4	0.313 (3)	0.406 (2)	0.8533 (16)	0.021 (9)*	
C5	0.3146 (4)	0.2571 (4)	0.8709 (2)	0.0355 (10)	
C6	0.2445 (5)	0.2004 (4)	0.9893 (2)	0.0522 (13)	
H6A	0.3261	0.1823	0.9996	0.063*	
H6B	0.2181	0.1375	0.9721	0.063*	
C7	0.1549 (5)	0.2435 (4)	1.0549 (2)	0.0625 (15)	
H7A	0.1501	0.1916	1.0918	0.094*	
H7B	0.1820	0.3056	1.0712	0.094*	
H7C	0.0746	0.2610	1.0438	0.094*	
C101	0.1249 (4)	0.1183 (3)	0.7955 (2)	0.0318 (9)	
C102	0.0582 (4)	0.1593 (3)	0.8602 (2)	0.0379 (10)	
H102	0.0496	0.2317	0.8689	0.046*	
C103	0.0044 (4)	0.0949 (4)	0.9122 (2)	0.0486 (12)	
H103	−0.0408	0.1236	0.9560	0.058*	
C104	0.0168 (5)	−0.0112 (4)	0.9002 (3)	0.0498 (12)	
H104	−0.0205	−0.0547	0.9356	0.060*	
C105	0.0834 (5)	−0.0537 (4)	0.8366 (3)	0.0516 (13)	
H105	0.0928	−0.1262	0.8286	0.062*	
C106	0.1362 (4)	0.0110 (3)	0.7848 (3)	0.0434 (11)	
H106	0.1809	−0.0181	0.7411	0.052*	
C107	0.1414 (4)	0.1673 (3)	0.6485 (2)	0.0378 (10)	
C108	0.0346 (4)	0.2249 (4)	0.6382 (3)	0.0497 (12)	
H108	−0.0046	0.2783	0.6709	0.060*	
C109	−0.0149 (5)	0.2047 (5)	0.5806 (3)	0.0643 (15)	
H109	−0.0866	0.2452	0.5736	0.077*	
C110	0.0406 (6)	0.1255 (5)	0.5335 (3)	0.0779 (19)	
H110	0.0062	0.1109	0.4947	0.093*	
C111	0.1454 (7)	0.0682 (5)	0.5430 (3)	0.088 (2)	
H111	0.1836	0.0144	0.5104	0.106*	
C112	0.1964 (5)	0.0885 (4)	0.6006 (3)	0.0651 (16)	
H112	0.2687	0.0483	0.6068	0.078*	
C201	0.5972 (4)	0.1633 (3)	0.7380 (2)	0.0353 (10)	
C202	0.6859 (4)	0.2193 (4)	0.7544 (2)	0.0464 (12)	
H202	0.6763	0.2924	0.7511	0.056*	
C203	0.7880 (4)	0.1694 (5)	0.7753 (3)	0.0574 (14)	
H203	0.8472	0.2080	0.7868	0.069*	
C204	0.8020 (5)	0.0625 (5)	0.7790 (3)	0.0674 (16)	
H204	0.8720	0.0279	0.7925	0.081*	
C205	0.7154 (5)	0.0058 (4)	0.7634 (3)	0.0660 (16)	
H205	0.7256	−0.0672	0.7668	0.079*	
C206	0.6130 (4)	0.0555 (4)	0.7425 (3)	0.0504 (12)	
H206	0.5541	0.0162	0.7314	0.061*	
C207	0.5223 (4)	0.2456 (3)	0.6120 (2)	0.0345 (10)	
C208	0.4374 (4)	0.2733 (3)	0.5700 (2)	0.0411 (11)	
H208	0.3542	0.2910	0.5912	0.049*	
C209	0.4752 (5)	0.2748 (4)	0.4969 (2)	0.0530 (13)	
H209	0.4176	0.2930	0.4685	0.064*	
C210	0.5969 (5)	0.2499 (4)	0.4655 (3)	0.0602 (14)	
H210	0.6220	0.2506	0.4158	0.072*	
C211	0.6822 (5)	0.2238 (4)	0.5066 (3)	0.0619 (15)	
H211	0.7656	0.2085	0.4849	0.074*	
C212	0.6456 (4)	0.2203 (4)	0.5801 (2)	0.0470 (12)	
H212	0.7036	0.2009	0.6081	0.056*	
C301	0.6002 (4)	0.4688 (3)	0.6643 (2)	0.0342 (10)	
C302	0.5845 (4)	0.4801 (3)	0.5945 (2)	0.0401 (11)	
H302	0.5055	0.4828	0.5849	0.048*	
C303	0.6836 (4)	0.4873 (4)	0.5395 (3)	0.0511 (13)	
H303	0.6725	0.4945	0.4925	0.061*	
C304	0.7992 (5)	0.4838 (4)	0.5537 (3)	0.0596 (14)	
H304	0.8667	0.4888	0.5160	0.072*	
C305	0.8176 (4)	0.4733 (4)	0.6219 (3)	0.0570 (14)	
H305	0.8971	0.4704	0.6307	0.068*	
C306	0.7178 (4)	0.4669 (4)	0.6779 (2)	0.0430 (11)	
H306	0.7295	0.4614	0.7248	0.052*	
C307	0.5217 (4)	0.4991 (4)	0.8154 (2)	0.0411 (11)	
C308	0.5459 (4)	0.4239 (4)	0.8648 (2)	0.0504 (13)	
H308	0.5278	0.3555	0.8601	0.060*	
C309	0.5969 (5)	0.4504 (5)	0.9211 (3)	0.0668 (16)	
H309	0.6150	0.3997	0.9540	0.080*	
C310	0.6206 (6)	0.5507 (6)	0.9284 (3)	0.082 (2)	
H310	0.6544	0.5684	0.9668	0.098*	
C311	0.5960 (6)	0.6261 (5)	0.8810 (3)	0.0756 (18)	
H311	0.6119	0.6948	0.8874	0.091*	
C312	0.5475 (5)	0.6009 (4)	0.8232 (3)	0.0577 (14)	
H312	0.5323	0.6519	0.7899	0.069*	
C401	0.1033 (4)	0.6511 (3)	0.7316 (2)	0.0416 (11)	
C402	−0.0197 (5)	0.6456 (5)	0.7435 (4)	0.085 (2)	
H402	−0.0531	0.5918	0.7714	0.103*	
C403	−0.0963 (6)	0.7187 (5)	0.7150 (5)	0.105 (3)	
H403	−0.1804	0.7123	0.7221	0.126*	
C404	−0.0511 (7)	0.7988 (5)	0.6771 (3)	0.0805 (19)	
H404	−0.1032	0.8469	0.6569	0.097*	
C405	0.0713 (7)	0.8101 (4)	0.6681 (3)	0.0760 (19)	
H405	0.1023	0.8673	0.6431	0.091*	
C406	0.1491 (5)	0.7372 (4)	0.6958 (3)	0.0628 (15)	
H406	0.2324	0.7458	0.6906	0.075*	
C407	0.1915 (4)	0.5998 (3)	0.8588 (2)	0.0380 (10)	
C408	0.1376 (4)	0.5429 (4)	0.9163 (2)	0.0441 (11)	
H408	0.1069	0.4805	0.9082	0.053*	
C409	0.1289 (4)	0.5774 (4)	0.9851 (3)	0.0510 (13)	
H409	0.0932	0.5382	1.0236	0.061*	
C410	0.1727 (4)	0.6695 (4)	0.9971 (3)	0.0531 (13)	
H410	0.1668	0.6932	1.0439	0.064*	
C411	0.2245 (4)	0.7260 (4)	0.9410 (3)	0.0520 (13)	
H411	0.2535	0.7890	0.9494	0.062*	
C412	0.2349 (4)	0.6920 (4)	0.8722 (2)	0.0442 (11)	
H412	0.2715	0.7316	0.8341	0.053*	
C8	0.3855 (8)	−0.1059 (8)	1.0516 (4)	0.124 (3)	
O3	0.4612 (6)	−0.1618 (6)	0.9936 (4)	0.156 (3)	
O4	0.3991 (6)	−0.0653 (4)	0.8766 (3)	0.127 (2)	
O5	0.5148 (8)	0.9672 (4)	0.5852 (4)	0.171 (3)	

### (Bis{[(diphenylphosphanyl)methyl]diphenylphosphanylidene}(ethoxyoxoethanylidene)methane-κ4P,C,C',P')dichloridoiridium(III) chloride–methanol–water (1/1/2) (6) Atomic displacement parameters (Å^2^)

**Table:**

	*U*^11^	*U*^22^	*U*^33^	*U*^12^	*U*^13^	*U*^23^
Ir1	0.02499 (9)	0.03004 (10)	0.03009 (10)	−0.00432 (6)	−0.00380 (6)	0.00424 (6)
P1	0.0260 (6)	0.0305 (6)	0.0336 (6)	−0.0062 (5)	−0.0062 (5)	0.0035 (5)
P2	0.0254 (6)	0.0334 (6)	0.0328 (6)	−0.0032 (5)	−0.0043 (4)	0.0021 (5)
P3	0.0293 (6)	0.0358 (6)	0.0300 (6)	−0.0091 (5)	−0.0036 (5)	0.0019 (5)
P4	0.0310 (6)	0.0299 (6)	0.0332 (6)	−0.0039 (5)	0.0000 (5)	0.0030 (5)
Cl1	0.0270 (5)	0.0447 (7)	0.0494 (7)	−0.0032 (5)	−0.0003 (5)	0.0070 (5)
Cl2	0.0394 (6)	0.0490 (7)	0.0335 (6)	0.0004 (5)	−0.0074 (5)	0.0092 (5)
Cl3	0.1165 (14)	0.0610 (10)	0.0883 (11)	−0.0425 (10)	−0.0108 (10)	0.0024 (8)
O1	0.048 (2)	0.044 (2)	0.0483 (19)	0.0049 (16)	−0.0053 (15)	0.0074 (15)
O2	0.0508 (19)	0.0459 (19)	0.0287 (16)	−0.0077 (15)	−0.0043 (14)	0.0107 (13)
C1	0.030 (2)	0.030 (2)	0.034 (2)	−0.0041 (18)	−0.0044 (18)	0.0008 (18)
C2	0.030 (2)	0.033 (2)	0.040 (2)	−0.0042 (19)	−0.0038 (19)	0.0006 (19)
C3	0.038 (2)	0.029 (2)	0.036 (2)	−0.0079 (19)	−0.0013 (19)	0.0037 (18)
C4	0.032 (2)	0.034 (2)	0.027 (2)	−0.0089 (19)	−0.0041 (18)	0.0009 (18)
C5	0.029 (2)	0.046 (3)	0.034 (2)	−0.009 (2)	−0.0101 (19)	0.003 (2)
C6	0.058 (3)	0.061 (3)	0.039 (3)	−0.011 (3)	−0.011 (2)	0.023 (2)
C7	0.076 (4)	0.075 (4)	0.039 (3)	−0.028 (3)	−0.006 (3)	0.012 (3)
C101	0.025 (2)	0.030 (2)	0.042 (2)	−0.0065 (18)	−0.0106 (19)	0.0082 (19)
C102	0.036 (2)	0.038 (3)	0.040 (3)	−0.004 (2)	−0.010 (2)	0.004 (2)
C103	0.052 (3)	0.054 (3)	0.040 (3)	−0.013 (2)	−0.007 (2)	0.011 (2)
C104	0.053 (3)	0.053 (3)	0.046 (3)	−0.015 (3)	−0.013 (2)	0.022 (2)
C105	0.058 (3)	0.035 (3)	0.061 (3)	−0.007 (2)	−0.009 (3)	0.015 (2)
C106	0.041 (3)	0.036 (3)	0.050 (3)	−0.002 (2)	−0.003 (2)	0.006 (2)
C107	0.039 (3)	0.038 (3)	0.040 (2)	−0.011 (2)	−0.012 (2)	0.003 (2)
C108	0.037 (3)	0.062 (3)	0.052 (3)	−0.005 (2)	−0.015 (2)	−0.002 (2)
C109	0.053 (3)	0.079 (4)	0.070 (4)	−0.006 (3)	−0.034 (3)	−0.004 (3)
C110	0.095 (5)	0.077 (4)	0.078 (4)	−0.010 (4)	−0.056 (4)	−0.009 (4)
C111	0.120 (6)	0.070 (4)	0.086 (5)	0.010 (4)	−0.060 (4)	−0.038 (4)
C112	0.082 (4)	0.051 (3)	0.069 (4)	0.010 (3)	−0.039 (3)	−0.017 (3)
C201	0.030 (2)	0.038 (3)	0.034 (2)	0.002 (2)	−0.0022 (18)	0.0012 (19)
C202	0.040 (3)	0.047 (3)	0.056 (3)	−0.014 (2)	−0.016 (2)	0.010 (2)
C203	0.037 (3)	0.073 (4)	0.068 (3)	−0.007 (3)	−0.025 (3)	0.012 (3)
C204	0.041 (3)	0.076 (4)	0.086 (4)	0.009 (3)	−0.028 (3)	0.021 (3)
C205	0.051 (3)	0.044 (3)	0.104 (5)	0.008 (3)	−0.025 (3)	0.007 (3)
C206	0.034 (3)	0.042 (3)	0.077 (4)	−0.002 (2)	−0.016 (2)	0.000 (2)
C207	0.034 (2)	0.033 (2)	0.034 (2)	−0.0073 (19)	−0.0010 (19)	0.0009 (18)
C208	0.040 (3)	0.041 (3)	0.041 (3)	−0.005 (2)	−0.006 (2)	0.002 (2)
C209	0.062 (3)	0.060 (3)	0.038 (3)	−0.003 (3)	−0.014 (2)	0.007 (2)
C210	0.069 (4)	0.070 (4)	0.035 (3)	−0.001 (3)	0.001 (3)	0.003 (3)
C211	0.046 (3)	0.082 (4)	0.047 (3)	−0.001 (3)	0.009 (3)	−0.001 (3)
C212	0.033 (3)	0.060 (3)	0.044 (3)	−0.004 (2)	−0.001 (2)	0.001 (2)
C301	0.029 (2)	0.034 (2)	0.038 (2)	−0.0064 (19)	−0.0035 (19)	0.0058 (19)
C302	0.034 (2)	0.047 (3)	0.036 (3)	−0.006 (2)	−0.0008 (19)	0.006 (2)
C303	0.047 (3)	0.060 (3)	0.039 (3)	0.003 (2)	0.001 (2)	0.010 (2)
C304	0.042 (3)	0.073 (4)	0.051 (3)	0.000 (3)	0.013 (2)	0.009 (3)
C305	0.028 (3)	0.067 (4)	0.070 (4)	−0.007 (2)	0.002 (2)	0.013 (3)
C306	0.033 (2)	0.050 (3)	0.049 (3)	−0.013 (2)	−0.011 (2)	0.012 (2)
C307	0.032 (2)	0.052 (3)	0.039 (3)	−0.013 (2)	−0.003 (2)	−0.006 (2)
C308	0.045 (3)	0.071 (4)	0.039 (3)	−0.021 (3)	−0.011 (2)	0.002 (2)
C309	0.055 (3)	0.111 (5)	0.041 (3)	−0.025 (3)	−0.019 (3)	0.011 (3)
C310	0.077 (4)	0.126 (6)	0.052 (4)	−0.040 (4)	−0.021 (3)	−0.014 (4)
C311	0.076 (4)	0.083 (5)	0.079 (4)	−0.036 (4)	−0.026 (3)	−0.021 (4)
C312	0.056 (3)	0.060 (3)	0.059 (3)	−0.017 (3)	−0.012 (3)	−0.011 (3)
C401	0.046 (3)	0.035 (3)	0.040 (3)	0.003 (2)	−0.005 (2)	0.003 (2)
C402	0.047 (3)	0.053 (4)	0.157 (7)	−0.003 (3)	−0.027 (4)	0.045 (4)
C403	0.061 (4)	0.060 (4)	0.197 (9)	0.000 (3)	−0.041 (5)	0.035 (5)
C404	0.084 (5)	0.065 (4)	0.086 (5)	0.031 (4)	−0.025 (4)	0.011 (3)
C405	0.099 (5)	0.050 (4)	0.064 (4)	0.014 (3)	0.001 (3)	0.022 (3)
C406	0.068 (4)	0.051 (3)	0.060 (3)	0.000 (3)	0.004 (3)	0.013 (3)
C407	0.036 (2)	0.036 (3)	0.040 (3)	−0.005 (2)	−0.001 (2)	0.002 (2)
C408	0.046 (3)	0.041 (3)	0.040 (3)	−0.009 (2)	0.003 (2)	−0.001 (2)
C409	0.047 (3)	0.056 (3)	0.044 (3)	−0.009 (3)	0.006 (2)	0.004 (2)
C410	0.048 (3)	0.065 (4)	0.041 (3)	−0.002 (3)	−0.001 (2)	−0.009 (3)
C411	0.051 (3)	0.050 (3)	0.055 (3)	−0.011 (2)	−0.006 (2)	−0.013 (3)
C412	0.044 (3)	0.041 (3)	0.044 (3)	−0.007 (2)	0.000 (2)	0.001 (2)
C8	0.114 (7)	0.164 (9)	0.085 (6)	0.000 (6)	−0.011 (5)	−0.033 (6)
O3	0.119 (5)	0.197 (7)	0.158 (6)	0.022 (5)	−0.060 (4)	−0.045 (5)
O4	0.188 (6)	0.105 (4)	0.098 (4)	−0.055 (4)	−0.035 (4)	0.022 (3)
O5	0.305 (10)	0.074 (4)	0.157 (6)	0.004 (5)	−0.112 (6)	−0.028 (4)

### (Bis{[(diphenylphosphanyl)methyl]diphenylphosphanylidene}(ethoxyoxoethanylidene)methane-κ4P,C,C',P')dichloridoiridium(III) chloride–methanol–water (1/1/2) (6) Geometric parameters (Å, º)

**Table:**

Ir1—C4	2.076 (4)	C203—H203	0.9400
Ir1—C1	2.149 (4)	C204—C205	1.369 (7)
Ir1—P1	2.3093 (11)	C204—H204	0.9400
Ir1—P4	2.3298 (11)	C205—C206	1.381 (7)
Ir1—Cl1	2.4268 (10)	C205—H205	0.9400
Ir1—Cl2	2.4597 (10)	C206—H206	0.9400
P1—C101	1.825 (4)	C207—C208	1.389 (6)
P1—C107	1.830 (4)	C207—C212	1.394 (6)
P1—C2	1.838 (4)	C208—C209	1.382 (6)
P2—C2	1.790 (4)	C208—H208	0.9400
P2—C207	1.800 (4)	C209—C210	1.374 (7)
P2—C201	1.801 (4)	C209—H209	0.9400
P2—C1	1.822 (4)	C210—C211	1.376 (7)
P3—C307	1.793 (4)	C210—H210	0.9400
P3—C3	1.800 (4)	C211—C212	1.389 (6)
P3—C301	1.809 (4)	C211—H211	0.9400
P3—C1	1.833 (4)	C212—H212	0.9400
P4—C407	1.821 (4)	C301—C302	1.395 (6)
P4—C401	1.825 (4)	C301—C306	1.397 (6)
P4—C3	1.835 (4)	C302—C303	1.378 (6)
O1—C5	1.213 (5)	C302—H302	0.9400
O2—C5	1.333 (5)	C303—C304	1.378 (7)
O2—C6	1.472 (5)	C303—H303	0.9400
C1—C4	1.513 (5)	C304—C305	1.373 (7)
C2—H2A	0.9800	C304—H304	0.9400
C2—H2B	0.9800	C305—C306	1.392 (6)
C3—H3A	0.9800	C305—H305	0.9400
C3—H3B	0.9800	C306—H306	0.9400
C4—C5	1.483 (6)	C307—C308	1.396 (7)
C4—H4	0.954 (18)	C307—C312	1.397 (7)
C6—C7	1.504 (7)	C308—C309	1.391 (7)
C6—H6A	0.9800	C308—H308	0.9400
C6—H6B	0.9800	C309—C310	1.368 (9)
C7—H7A	0.9700	C309—H309	0.9400
C7—H7B	0.9700	C310—C311	1.371 (9)
C7—H7C	0.9700	C310—H310	0.9400
C101—C102	1.385 (6)	C311—C312	1.394 (7)
C101—C106	1.389 (6)	C311—H311	0.9400
C102—C103	1.382 (6)	C312—H312	0.9400
C102—H102	0.9400	C401—C402	1.364 (7)
C103—C104	1.379 (7)	C401—C406	1.396 (7)
C103—H103	0.9400	C402—C403	1.388 (8)
C104—C105	1.373 (7)	C402—H402	0.9400
C104—H104	0.9400	C403—C404	1.346 (9)
C105—C106	1.378 (6)	C403—H403	0.9400
C105—H105	0.9400	C404—C405	1.375 (9)
C106—H106	0.9400	C404—H404	0.9400
C107—C112	1.376 (7)	C405—C406	1.387 (8)
C107—C108	1.383 (6)	C405—H405	0.9400
C108—C109	1.381 (7)	C406—H406	0.9400
C108—H108	0.9400	C407—C412	1.386 (6)
C109—C110	1.373 (8)	C407—C408	1.393 (6)
C109—H109	0.9400	C408—C409	1.380 (6)
C110—C111	1.358 (8)	C408—H408	0.9400
C110—H110	0.9400	C409—C410	1.380 (7)
C111—C112	1.391 (7)	C409—H409	0.9400
C111—H111	0.9400	C410—C411	1.364 (7)
C112—H112	0.9400	C410—H410	0.9400
C201—C206	1.386 (6)	C411—C412	1.376 (6)
C201—C202	1.387 (6)	C411—H411	0.9400
C202—C203	1.381 (6)	C412—H412	0.9400
C202—H202	0.9400	C8—O3	1.400 (9)
C203—C204	1.373 (8)		
			
C4—Ir1—C1	41.94 (15)	C107—C112—C111	120.2 (5)
C4—Ir1—P1	93.94 (12)	C107—C112—H112	119.9
C1—Ir1—P1	90.24 (11)	C111—C112—H112	119.9
C4—Ir1—P4	87.46 (12)	C206—C201—C202	118.8 (4)
C1—Ir1—P4	89.52 (11)	C206—C201—P2	119.9 (3)
P1—Ir1—P4	177.61 (4)	C202—C201—P2	121.1 (3)
C4—Ir1—Cl1	116.21 (11)	C203—C202—C201	121.0 (5)
C1—Ir1—Cl1	158.13 (11)	C203—C202—H202	119.5
P1—Ir1—Cl1	90.79 (4)	C201—C202—H202	119.5
P4—Ir1—Cl1	90.32 (4)	C204—C203—C202	119.1 (5)
C4—Ir1—Cl2	151.91 (11)	C204—C203—H203	120.5
C1—Ir1—Cl2	111.50 (11)	C202—C203—H203	120.5
P1—Ir1—Cl2	95.28 (4)	C205—C204—C203	120.8 (5)
P4—Ir1—Cl2	82.61 (4)	C205—C204—H204	119.6
Cl1—Ir1—Cl2	90.15 (4)	C203—C204—H204	119.6
C101—P1—C107	103.22 (19)	C204—C205—C206	120.3 (5)
C101—P1—C2	101.10 (19)	C204—C205—H205	119.9
C107—P1—C2	105.4 (2)	C206—C205—H205	119.9
C101—P1—Ir1	119.47 (14)	C205—C206—C201	120.0 (5)
C107—P1—Ir1	116.96 (15)	C205—C206—H206	120.0
C2—P1—Ir1	108.79 (13)	C201—C206—H206	120.0
C2—P2—C207	104.15 (19)	C208—C207—C212	119.6 (4)
C2—P2—C201	107.7 (2)	C208—C207—P2	119.1 (3)
C207—P2—C201	106.31 (19)	C212—C207—P2	120.8 (3)
C2—P2—C1	109.35 (19)	C209—C208—C207	120.0 (4)
C207—P2—C1	111.24 (19)	C209—C208—H208	120.0
C201—P2—C1	117.21 (19)	C207—C208—H208	120.0
C307—P3—C3	109.2 (2)	C210—C209—C208	120.2 (5)
C307—P3—C301	105.7 (2)	C210—C209—H209	119.9
C3—P3—C301	105.53 (19)	C208—C209—H209	119.9
C307—P3—C1	111.0 (2)	C209—C210—C211	120.4 (5)
C3—P3—C1	106.85 (19)	C209—C210—H210	119.8
C301—P3—C1	118.24 (19)	C211—C210—H210	119.8
C407—P4—C401	103.8 (2)	C210—C211—C212	120.2 (5)
C407—P4—C3	105.7 (2)	C210—C211—H211	119.9
C401—P4—C3	104.1 (2)	C212—C211—H211	119.9
C407—P4—Ir1	118.43 (14)	C211—C212—C207	119.5 (5)
C401—P4—Ir1	121.43 (15)	C211—C212—H212	120.2
C3—P4—Ir1	101.43 (14)	C207—C212—H212	120.2
C5—O2—C6	116.4 (4)	C302—C301—C306	119.1 (4)
C4—C1—P2	120.5 (3)	C302—C301—P3	120.6 (3)
C4—C1—P3	111.2 (3)	C306—C301—P3	120.1 (3)
P2—C1—P3	119.9 (2)	C303—C302—C301	120.5 (4)
C4—C1—Ir1	66.5 (2)	C303—C302—H302	119.7
P2—C1—Ir1	113.31 (19)	C301—C302—H302	119.7
P3—C1—Ir1	113.9 (2)	C302—C303—C304	119.7 (5)
P2—C2—P1	112.6 (2)	C302—C303—H303	120.2
P2—C2—H2A	109.1	C304—C303—H303	120.2
P1—C2—H2A	109.1	C305—C304—C303	121.2 (4)
P2—C2—H2B	109.1	C305—C304—H304	119.4
P1—C2—H2B	109.1	C303—C304—H304	119.4
H2A—C2—H2B	107.8	C304—C305—C306	119.6 (5)
P3—C3—P4	111.4 (2)	C304—C305—H305	120.2
P3—C3—H3A	109.3	C306—C305—H305	120.2
P4—C3—H3A	109.3	C305—C306—C301	119.9 (4)
P3—C3—H3B	109.3	C305—C306—H306	120.1
P4—C3—H3B	109.3	C301—C306—H306	120.1
H3A—C3—H3B	108.0	C308—C307—C312	119.7 (4)
C5—C4—C1	122.7 (4)	C308—C307—P3	122.1 (4)
C5—C4—Ir1	126.2 (3)	C312—C307—P3	118.0 (4)
C1—C4—Ir1	71.6 (2)	C309—C308—C307	120.0 (5)
C5—C4—H4	104 (2)	C309—C308—H308	120.0
C1—C4—H4	115 (2)	C307—C308—H308	120.0
Ir1—C4—H4	116 (2)	C310—C309—C308	119.6 (6)
O1—C5—O2	123.9 (4)	C310—C309—H309	120.2
O1—C5—C4	126.0 (4)	C308—C309—H309	120.2
O2—C5—C4	110.1 (4)	C309—C310—C311	121.4 (5)
O2—C6—C7	106.7 (4)	C309—C310—H310	119.3
O2—C6—H6A	110.4	C311—C310—H310	119.3
C7—C6—H6A	110.4	C310—C311—C312	120.1 (6)
O2—C6—H6B	110.4	C310—C311—H311	120.0
C7—C6—H6B	110.4	C312—C311—H311	120.0
H6A—C6—H6B	108.6	C311—C312—C307	119.2 (5)
C6—C7—H7A	109.5	C311—C312—H312	120.4
C6—C7—H7B	109.5	C307—C312—H312	120.4
H7A—C7—H7B	109.5	C402—C401—C406	118.5 (5)
C6—C7—H7C	109.5	C402—C401—P4	120.1 (4)
H7A—C7—H7C	109.5	C406—C401—P4	121.2 (4)
H7B—C7—H7C	109.5	C401—C402—C403	120.8 (6)
C102—C101—C106	118.1 (4)	C401—C402—H402	119.6
C102—C101—P1	122.0 (3)	C403—C402—H402	119.6
C106—C101—P1	119.9 (3)	C404—C403—C402	120.6 (6)
C103—C102—C101	120.6 (4)	C404—C403—H403	119.7
C103—C102—H102	119.7	C402—C403—H403	119.7
C101—C102—H102	119.7	C403—C404—C405	119.9 (6)
C104—C103—C102	120.1 (5)	C403—C404—H404	120.1
C104—C103—H103	119.9	C405—C404—H404	120.1
C102—C103—H103	119.9	C404—C405—C406	120.2 (6)
C105—C104—C103	120.2 (4)	C404—C405—H405	119.9
C105—C104—H104	119.9	C406—C405—H405	119.9
C103—C104—H104	119.9	C405—C406—C401	119.8 (6)
C104—C105—C106	119.3 (5)	C405—C406—H406	120.1
C104—C105—H105	120.3	C401—C406—H406	120.1
C106—C105—H105	120.3	C412—C407—C408	118.6 (4)
C105—C106—C101	121.6 (4)	C412—C407—P4	121.5 (3)
C105—C106—H106	119.2	C408—C407—P4	120.0 (3)
C101—C106—H106	119.2	C409—C408—C407	120.5 (4)
C112—C107—C108	118.7 (4)	C409—C408—H408	119.7
C112—C107—P1	123.8 (4)	C407—C408—H408	119.7
C108—C107—P1	117.5 (3)	C408—C409—C410	119.9 (5)
C109—C108—C107	120.7 (5)	C408—C409—H409	120.1
C109—C108—H108	119.7	C410—C409—H409	120.1
C107—C108—H108	119.7	C411—C410—C409	119.9 (5)
C110—C109—C108	120.0 (5)	C411—C410—H410	120.1
C110—C109—H109	120.0	C409—C410—H410	120.1
C108—C109—H109	120.0	C410—C411—C412	120.9 (5)
C111—C110—C109	119.9 (5)	C410—C411—H411	119.6
C111—C110—H110	120.0	C412—C411—H411	119.6
C109—C110—H110	120.0	C411—C412—C407	120.3 (4)
C110—C111—C112	120.5 (6)	C411—C412—H412	119.9
C110—C111—H111	119.8	C407—C412—H412	119.9
C112—C111—H111	119.8		
			
C2—P2—C1—C4	48.9 (4)	P2—C201—C202—C203	−176.4 (4)
C207—P2—C1—C4	163.4 (3)	C201—C202—C203—C204	0.8 (8)
C201—P2—C1—C4	−74.0 (4)	C202—C203—C204—C205	−1.0 (9)
C2—P2—C1—P3	−165.7 (2)	C203—C204—C205—C206	1.0 (9)
C207—P2—C1—P3	−51.2 (3)	C204—C205—C206—C201	−0.6 (9)
C201—P2—C1—P3	71.4 (3)	C202—C201—C206—C205	0.4 (7)
C2—P2—C1—Ir1	−26.5 (3)	P2—C201—C206—C205	176.3 (4)
C207—P2—C1—Ir1	88.0 (2)	C2—P2—C207—C208	53.0 (4)
C201—P2—C1—Ir1	−149.5 (2)	C201—P2—C207—C208	166.7 (3)
C307—P3—C1—C4	41.4 (3)	C1—P2—C207—C208	−64.7 (4)
C3—P3—C1—C4	−77.6 (3)	C2—P2—C207—C212	−119.3 (4)
C301—P3—C1—C4	163.7 (3)	C201—P2—C207—C212	−5.6 (4)
C307—P3—C1—P2	−107.0 (3)	C1—P2—C207—C212	123.0 (4)
C3—P3—C1—P2	134.1 (2)	C212—C207—C208—C209	0.6 (7)
C301—P3—C1—P2	15.4 (3)	P2—C207—C208—C209	−171.8 (4)
C307—P3—C1—Ir1	114.1 (2)	C207—C208—C209—C210	−0.6 (7)
C3—P3—C1—Ir1	−4.9 (3)	C208—C209—C210—C211	−0.5 (8)
C301—P3—C1—Ir1	−123.6 (2)	C209—C210—C211—C212	1.5 (9)
C207—P2—C2—P1	−98.2 (2)	C210—C211—C212—C207	−1.5 (8)
C201—P2—C2—P1	149.2 (2)	C208—C207—C212—C211	0.5 (7)
C1—P2—C2—P1	20.8 (3)	P2—C207—C212—C211	172.7 (4)
C101—P1—C2—P2	−133.9 (2)	C307—P3—C301—C302	−159.6 (4)
C107—P1—C2—P2	118.9 (2)	C3—P3—C301—C302	−44.0 (4)
Ir1—P1—C2—P2	−7.3 (3)	C1—P3—C301—C302	75.4 (4)
C307—P3—C3—P4	−87.5 (3)	C307—P3—C301—C306	15.4 (4)
C301—P3—C3—P4	159.3 (2)	C3—P3—C301—C306	131.0 (4)
C1—P3—C3—P4	32.7 (3)	C1—P3—C301—C306	−109.6 (4)
C407—P4—C3—P3	81.0 (3)	C306—C301—C302—C303	1.2 (7)
C401—P4—C3—P3	−170.0 (2)	P3—C301—C302—C303	176.2 (4)
Ir1—P4—C3—P3	−43.2 (2)	C301—C302—C303—C304	−0.3 (7)
P2—C1—C4—C5	17.6 (5)	C302—C303—C304—C305	0.0 (8)
P3—C1—C4—C5	−130.5 (3)	C303—C304—C305—C306	−0.6 (8)
Ir1—C1—C4—C5	121.8 (4)	C304—C305—C306—C301	1.5 (8)
P2—C1—C4—Ir1	−104.1 (3)	C302—C301—C306—C305	−1.8 (7)
P3—C1—C4—Ir1	107.8 (2)	P3—C301—C306—C305	−176.9 (4)
C6—O2—C5—O1	1.6 (6)	C3—P3—C307—C308	141.9 (4)
C6—O2—C5—C4	−178.7 (3)	C301—P3—C307—C308	−105.0 (4)
C1—C4—C5—O1	−9.5 (6)	C1—P3—C307—C308	24.3 (4)
Ir1—C4—C5—O1	80.8 (5)	C3—P3—C307—C312	−43.2 (4)
C1—C4—C5—O2	170.8 (3)	C301—P3—C307—C312	69.9 (4)
Ir1—C4—C5—O2	−98.9 (4)	C1—P3—C307—C312	−160.7 (4)
C5—O2—C6—C7	−174.9 (4)	C312—C307—C308—C309	−0.7 (7)
C107—P1—C101—C102	−126.3 (3)	P3—C307—C308—C309	174.1 (4)
C2—P1—C101—C102	124.8 (3)	C307—C308—C309—C310	1.3 (8)
Ir1—P1—C101—C102	5.6 (4)	C308—C309—C310—C311	−0.5 (10)
C107—P1—C101—C106	55.4 (4)	C309—C310—C311—C312	−0.9 (10)
C2—P1—C101—C106	−53.5 (4)	C310—C311—C312—C307	1.6 (9)
Ir1—P1—C101—C106	−172.7 (3)	C308—C307—C312—C311	−0.7 (7)
C106—C101—C102—C103	−0.3 (6)	P3—C307—C312—C311	−175.8 (4)
P1—C101—C102—C103	−178.6 (3)	C407—P4—C401—C402	−84.0 (5)
C101—C102—C103—C104	0.2 (7)	C3—P4—C401—C402	165.6 (5)
C102—C103—C104—C105	0.4 (7)	Ir1—P4—C401—C402	52.5 (5)
C103—C104—C105—C106	−0.9 (7)	C407—P4—C401—C406	90.9 (4)
C104—C105—C106—C101	0.7 (7)	C3—P4—C401—C406	−19.6 (4)
C102—C101—C106—C105	−0.1 (6)	Ir1—P4—C401—C406	−132.7 (4)
P1—C101—C106—C105	178.2 (4)	C406—C401—C402—C403	6.0 (10)
C101—P1—C107—C112	−89.9 (5)	P4—C401—C402—C403	−178.9 (6)
C2—P1—C107—C112	15.7 (5)	C401—C402—C403—C404	−2.5 (12)
Ir1—P1—C107—C112	136.7 (4)	C402—C403—C404—C405	−1.7 (12)
C101—P1—C107—C108	89.5 (4)	C403—C404—C405—C406	2.2 (10)
C2—P1—C107—C108	−164.9 (4)	C404—C405—C406—C401	1.4 (9)
Ir1—P1—C107—C108	−43.8 (4)	C402—C401—C406—C405	−5.5 (8)
C112—C107—C108—C109	−0.9 (8)	P4—C401—C406—C405	179.6 (4)
P1—C107—C108—C109	179.7 (4)	C401—P4—C407—C412	−65.3 (4)
C107—C108—C109—C110	1.3 (9)	C3—P4—C407—C412	43.9 (4)
C108—C109—C110—C111	−1.1 (10)	Ir1—P4—C407—C412	156.7 (3)
C109—C110—C111—C112	0.6 (11)	C401—P4—C407—C408	114.3 (4)
C108—C107—C112—C111	0.4 (8)	C3—P4—C407—C408	−136.5 (4)
P1—C107—C112—C111	179.8 (5)	Ir1—P4—C407—C408	−23.8 (4)
C110—C111—C112—C107	−0.3 (11)	C412—C407—C408—C409	−0.7 (7)
C2—P2—C201—C206	16.9 (4)	P4—C407—C408—C409	179.8 (4)
C207—P2—C201—C206	−94.3 (4)	C407—C408—C409—C410	0.7 (7)
C1—P2—C201—C206	140.7 (4)	C408—C409—C410—C411	−0.1 (8)
C2—P2—C201—C202	−167.2 (4)	C409—C410—C411—C412	−0.6 (8)
C207—P2—C201—C202	81.6 (4)	C410—C411—C412—C407	0.6 (7)
C1—P2—C201—C202	−43.5 (4)	C408—C407—C412—C411	0.0 (7)
C206—C201—C202—C203	−0.4 (7)	P4—C407—C412—C411	179.6 (4)

### (Bis{[(diphenylphosphanyl)methyl]diphenylphosphanylidene}(ethoxyoxoethanylidene)methane-κ4P,C,C',P')dichloridoiridium(III) chloride–methanol–water (1/1/2) (6) Hydrogen-bond geometry (Å, º)

**Table:**

*D*—H···*A*	*D*—H	H···*A*	*D*···*A*	*D*—H···*A*
C2—H2*A*···O1	0.98	2.33	2.852 (5)	112
C2—H2*B*···O5^i^	0.98	2.45	3.320 (8)	148
C3—H3*B*···Cl3	0.98	2.66	3.589 (4)	158
C6—H6*A*···O3^ii^	0.98	2.40	3.369 (8)	169
C102—H102···Cl1	0.94	2.63	3.343 (4)	133
C108—H108···Cl1	0.94	2.82	3.671 (5)	151
C206—H206···Cl3^i^	0.94	2.87	3.742 (5)	156
C208—H208···Cl2	0.94	2.64	3.487 (5)	150
C312—H312···Cl3	0.94	2.84	3.749 (6)	164
C402—H402···Cl1	0.94	2.59	3.398 (6)	144
C406—H406···Cl3	0.94	2.88	3.757 (6)	156
C412—H412···Cl3	0.94	2.95	3.870 (5)	167

Symmetry codes: (i) *x*, *y*−1, *z*; (ii) −*x*+1, −*y*, −*z*+2.

### (Bis{[(diphenylphosphanyl)methyl]diphenylphosphanylidene}(ethoxyoxoethanylidene)methane-κ4P,C,C',P')carbonyl(ethoxyoxoethanide)iridium(III) dichloride–methylene chloride–water (1/2/1.5) (7) Crystal data

**Table:**

[Ir(C_4_H_7_O_2_)(C_55_H_50_O_2_P_4_)(CO)]Cl_2_·2CH_2_Cl_2_·1.5H_2_O	*Z* = 2
*M**_r_* = 1441.91	*F*(000) = 1454
Triclinic, *P*1	*D*_x_ = 1.505 Mg m^−^^3^
*a* = 11.7326 (2) Å	Mo *K*α radiation, λ = 0.71073 Å
*b* = 13.8815 (2) Å	Cell parameters from 109564 reflections
*c* = 22.2615 (3) Å	θ = 1.0–27.4°
α = 75.477 (1)°	µ = 2.50 mm^−^^1^
β = 86.508 (1)°	*T* = 233 K
γ = 65.212 (1)°	Prism, yellow
*V* = 3182.38 (9) Å^3^	0.31 × 0.23 × 0.19 mm

### (Bis{[(diphenylphosphanyl)methyl]diphenylphosphanylidene}(ethoxyoxoethanylidene)methane-κ4P,C,C',P')carbonyl(ethoxyoxoethanide)iridium(III) dichloride–methylene chloride–water (1/2/1.5) (7) Data collection

**Table:**

Nonius KappaCCD diffractometer	11695 reflections with *I* > 2σ(*I*)
Radiation source: fine-focus sealed tube	*R*_int_ = 0.024
Graphite monochromator	θ_max_ = 26.0°, θ_min_ = 1.9°
phi– and ω–scans	*h* = −14→14
23329 measured reflections	*k* = −17→16
12496 independent reflections	*l* = −26→27

### (Bis{[(diphenylphosphanyl)methyl]diphenylphosphanylidene}(ethoxyoxoethanylidene)methane-κ4P,C,C',P')carbonyl(ethoxyoxoethanide)iridium(III) dichloride–methylene chloride–water (1/2/1.5) (7) Refinement

**Table:**

Refinement on *F*^2^	3 restraints
Least-squares matrix: full	Hydrogen site location: mixed
*R*[*F*^2^ > 2σ(*F*^2^)] = 0.028	H atoms treated by a mixture of independent and constrained refinement
*wR*(*F*^2^) = 0.070	*w* = 1/[σ^2^(*F*_o_^2^) + (0.026*P*)^2^ + 5.2527*P*] where *P* = (*F*_o_^2^ + 2*F*_c_^2^)/3
*S* = 1.05	(Δ/σ)_max_ = 0.001
12496 reflections	Δρ_max_ = 0.97 e Å^−^^3^
751 parameters	Δρ_min_ = −1.29 e Å^−^^3^

### (Bis{[(diphenylphosphanyl)methyl]diphenylphosphanylidene}(ethoxyoxoethanylidene)methane-κ4P,C,C',P')carbonyl(ethoxyoxoethanide)iridium(III) dichloride–methylene chloride–water (1/2/1.5) (7) Special details

**Table:**

Experimental. All data sets were measured with several scans to increase the number of redundant reflections. In our experience this method of averaging redundant reflections replaces in a good approximation semi-empirical absorptions methods (absorption correction programs like SORTAV lead to no better data sets).
Geometry. All esds (except the esd in the dihedral angle between two l.s. planes) are estimated using the full covariance matrix. The cell esds are taken into account individually in the estimation of esds in distances, angles and torsion angles; correlations between esds in cell parameters are only used when they are defined by crystal symmetry. An approximate (isotropic) treatment of cell esds is used for estimating esds involving l.s. planes.
Refinement. Hydrogen at C4 was found and refined isotropically with bond restraint (d=96pm). Hydrogens at water O6 were found and refined isotropically with bond restraints (d=84pm). The water molecule O7 was half occupied and hydrogen of it were omitted. The chlorine atoms at solvent dichloromethane CL5-C14-Cl6 were positional disordered in ratio around 2:1 (CL5-6: Cl5A-6A). C14=C14A with equal coordinates and displacement parameters for hydrogen calculation.

### (Bis{[(diphenylphosphanyl)methyl]diphenylphosphanylidene}(ethoxyoxoethanylidene)methane-κ4P,C,C',P')carbonyl(ethoxyoxoethanide)iridium(III) dichloride–methylene chloride–water (1/2/1.5) (7) Fractional atomic coordinates and isotropic or equivalent isotropic displacement parameters (Å^2^)

**Table:**

	*x*	*y*	*z*	*U*_iso_*/*U*_eq_	Occ. (<1)
Ir1	0.18100 (2)	0.32675 (2)	0.19059 (2)	0.02360 (4)	
P1	−0.01647 (7)	0.32287 (7)	0.20407 (4)	0.02798 (16)	
P2	0.08519 (8)	0.24968 (7)	0.33707 (4)	0.02951 (17)	
P3	0.36173 (7)	0.23582 (6)	0.31877 (3)	0.02553 (16)	
P4	0.38428 (7)	0.32406 (6)	0.18358 (3)	0.02485 (15)	
O1	0.2405 (2)	0.4989 (2)	0.30318 (12)	0.0441 (6)	
O2	0.0747 (2)	0.60098 (18)	0.23599 (11)	0.0398 (6)	
O3	0.2584 (3)	0.4317 (3)	0.02412 (12)	0.0555 (7)	
O4	0.1299 (3)	0.3486 (2)	0.02816 (11)	0.0512 (7)	
O5	0.2606 (3)	0.1321 (2)	0.13439 (14)	0.0542 (7)	
C1	0.1994 (3)	0.2948 (2)	0.29337 (13)	0.0258 (6)	
C2	0.0016 (3)	0.2266 (3)	0.28035 (14)	0.0322 (7)	
H2A	−0.0816	0.2349	0.2950	0.039*	
H2B	0.0475	0.1515	0.2761	0.039*	
C3	0.4342 (3)	0.3087 (3)	0.26346 (13)	0.0275 (6)	
H3A	0.5259	0.2688	0.2690	0.033*	
H3B	0.4109	0.3813	0.2707	0.033*	
C4	0.1312 (3)	0.4148 (2)	0.26037 (13)	0.0264 (6)	
H4	0.0434 (18)	0.441 (3)	0.2635 (15)	0.030 (9)*	
C5	0.1589 (3)	0.5053 (3)	0.26978 (15)	0.0320 (7)	
C6	0.0842 (4)	0.7004 (3)	0.2418 (2)	0.0530 (10)	
H6A	0.1702	0.6939	0.2351	0.064*	
H6B	0.0618	0.7123	0.2833	0.064*	
C7	−0.0043 (6)	0.7923 (4)	0.1938 (3)	0.093 (2)	
H7A	−0.0011	0.8601	0.1960	0.140*	
H7B	0.0190	0.7795	0.1530	0.140*	
H7C	−0.0889	0.7977	0.2010	0.140*	
C8	0.1075 (3)	0.4468 (3)	0.10236 (14)	0.0334 (7)	
H8A	0.0184	0.4633	0.0967	0.040*	
H8B	0.1127	0.5147	0.1041	0.040*	
C9	0.1736 (3)	0.4113 (3)	0.04821 (14)	0.0368 (7)	
C10	0.1946 (5)	0.3027 (4)	−0.0222 (2)	0.0668 (13)	
H10A	0.2829	0.2543	−0.0095	0.080*	
H10B	0.1916	0.3612	−0.0584	0.080*	
C11	0.1298 (7)	0.2398 (5)	−0.0379 (3)	0.099 (2)	
H11A	0.1708	0.2076	−0.0717	0.149*	
H11B	0.1335	0.1821	−0.0018	0.149*	
H11C	0.0426	0.2885	−0.0505	0.149*	
C12	0.2317 (3)	0.2051 (3)	0.15415 (15)	0.0331 (7)	
C101	−0.0676 (3)	0.2706 (3)	0.14917 (15)	0.0335 (7)	
C102	−0.0305 (4)	0.1594 (3)	0.15564 (19)	0.0524 (10)	
H102	0.0193	0.1079	0.1906	0.063*	
C103	−0.0678 (5)	0.1250 (4)	0.1099 (2)	0.0660 (13)	
H103	−0.0443	0.0498	0.1144	0.079*	
C104	−0.1382 (5)	0.1990 (4)	0.0586 (2)	0.0607 (12)	
H104	−0.1625	0.1743	0.0280	0.073*	
C105	−0.1736 (4)	0.3080 (4)	0.05112 (18)	0.0506 (10)	
H105	−0.2219	0.3583	0.0155	0.061*	
C106	−0.1383 (3)	0.3449 (3)	0.09598 (16)	0.0416 (8)	
H106	−0.1622	0.4203	0.0906	0.050*	
C107	−0.1551 (3)	0.4470 (3)	0.20753 (15)	0.0343 (7)	
C108	−0.1557 (3)	0.5505 (3)	0.19268 (16)	0.0397 (8)	
H108	−0.0819	0.5588	0.1798	0.048*	
C109	−0.2639 (4)	0.6416 (3)	0.1967 (2)	0.0544 (10)	
H109	−0.2636	0.7114	0.1869	0.065*	
C110	−0.3727 (4)	0.6289 (4)	0.2153 (2)	0.0639 (12)	
H110	−0.4463	0.6903	0.2186	0.077*	
C111	−0.3735 (4)	0.5262 (4)	0.2292 (2)	0.0586 (11)	
H111	−0.4478	0.5183	0.2416	0.070*	
C112	−0.2664 (3)	0.4361 (3)	0.22511 (17)	0.0454 (9)	
H112	−0.2678	0.3667	0.2341	0.055*	
C201	0.1375 (3)	0.1290 (3)	0.40066 (14)	0.0360 (7)	
C202	0.1785 (4)	0.0254 (3)	0.39114 (18)	0.0494 (9)	
H202	0.1838	0.0162	0.3505	0.059*	
C203	0.2117 (5)	−0.0648 (4)	0.4412 (2)	0.0710 (14)	
H203	0.2409	−0.1352	0.4344	0.085*	
C204	0.2025 (5)	−0.0524 (4)	0.5010 (2)	0.0754 (15)	
H204	0.2255	−0.1142	0.5348	0.091*	
C205	0.1602 (5)	0.0495 (4)	0.51097 (19)	0.0699 (14)	
H205	0.1536	0.0576	0.5519	0.084*	
C206	0.1266 (4)	0.1415 (4)	0.46165 (17)	0.0538 (10)	
H206	0.0968	0.2116	0.4690	0.065*	
C207	−0.0190 (3)	0.3598 (3)	0.36982 (15)	0.0404 (8)	
C208	0.0283 (4)	0.4210 (4)	0.39200 (18)	0.0542 (10)	
H208	0.1143	0.4053	0.3896	0.065*	
C209	−0.0505 (6)	0.5050 (5)	0.4177 (2)	0.0834 (17)	
H209	−0.0180	0.5465	0.4327	0.100*	
C210	−0.1743 (7)	0.5277 (6)	0.4213 (3)	0.100 (2)	
H210	−0.2280	0.5860	0.4379	0.121*	
C211	−0.2220 (5)	0.4656 (6)	0.4006 (3)	0.100 (2)	
H211	−0.3078	0.4811	0.4043	0.119*	
C212	−0.1449 (4)	0.3800 (4)	0.37442 (19)	0.0615 (12)	
H212	−0.1773	0.3375	0.3604	0.074*	
C301	0.3972 (3)	0.2405 (3)	0.39562 (14)	0.0319 (7)	
C302	0.3965 (4)	0.1578 (3)	0.44585 (16)	0.0452 (9)	
H302	0.3775	0.1016	0.4394	0.054*	
C303	0.4237 (5)	0.1584 (4)	0.50518 (18)	0.0617 (12)	
H303	0.4215	0.1033	0.5392	0.074*	
C304	0.4536 (5)	0.2385 (4)	0.51477 (18)	0.0635 (13)	
H304	0.4723	0.2378	0.5554	0.076*	
C305	0.4568 (4)	0.3201 (4)	0.46571 (19)	0.0589 (11)	
H305	0.4781	0.3747	0.4727	0.071*	
C306	0.4285 (4)	0.3214 (3)	0.40556 (17)	0.0444 (9)	
H306	0.4305	0.3770	0.3718	0.053*	
C307	0.4396 (3)	0.0935 (2)	0.31728 (14)	0.0294 (6)	
C308	0.3872 (3)	0.0411 (3)	0.28949 (16)	0.0382 (8)	
H308	0.3039	0.0785	0.2728	0.046*	
C309	0.4580 (4)	−0.0664 (3)	0.28642 (19)	0.0503 (9)	
H309	0.4217	−0.1023	0.2686	0.060*	
C310	0.5809 (4)	−0.1206 (3)	0.3093 (2)	0.0522 (10)	
H310	0.6287	−0.1930	0.3063	0.063*	
C311	0.6345 (4)	−0.0693 (3)	0.33646 (18)	0.0451 (9)	
H311	0.7187	−0.1065	0.3517	0.054*	
C312	0.5640 (3)	0.0370 (3)	0.34124 (15)	0.0362 (7)	
H312	0.5999	0.0712	0.3607	0.043*	
C401	0.4056 (3)	0.4445 (3)	0.13947 (14)	0.0298 (6)	
C402	0.3124 (3)	0.5476 (3)	0.13833 (16)	0.0397 (8)	
H402	0.2396	0.5549	0.1605	0.048*	
C403	0.3264 (4)	0.6402 (3)	0.10452 (19)	0.0488 (9)	
H403	0.2632	0.7101	0.1040	0.059*	
C404	0.4319 (4)	0.6305 (3)	0.07180 (18)	0.0481 (9)	
H404	0.4409	0.6936	0.0491	0.058*	
C405	0.5241 (4)	0.5287 (3)	0.07225 (18)	0.0478 (9)	
H405	0.5961	0.5221	0.0496	0.057*	
C406	0.5115 (3)	0.4355 (3)	0.10599 (16)	0.0394 (8)	
H406	0.5752	0.3660	0.1062	0.047*	
C407	0.5050 (3)	0.2106 (3)	0.15751 (15)	0.0316 (7)	
C408	0.4890 (4)	0.1989 (3)	0.09900 (18)	0.0458 (9)	
H408	0.4177	0.2500	0.0735	0.055*	
C409	0.5773 (4)	0.1125 (4)	0.0779 (2)	0.0590 (11)	
H409	0.5663	0.1051	0.0381	0.071*	
C410	0.6809 (5)	0.0375 (4)	0.1154 (2)	0.0652 (13)	
H410	0.7395	−0.0225	0.1016	0.078*	
C411	0.6996 (4)	0.0494 (4)	0.1723 (2)	0.0665 (13)	
H411	0.7718	−0.0017	0.1972	0.080*	
C412	0.6133 (4)	0.1358 (3)	0.19386 (18)	0.0474 (9)	
H412	0.6276	0.1443	0.2329	0.057*	
Cl1	−0.26323 (9)	0.19319 (10)	0.33214 (5)	0.0573 (3)	
Cl2	0.17302 (11)	−0.07900 (9)	0.26051 (5)	0.0607 (3)	
C13	0.3341 (7)	0.8877 (5)	0.1243 (3)	0.097 (2)	
H13A	0.2976	0.9296	0.1555	0.117*	
H13B	0.3525	0.8108	0.1435	0.117*	
Cl3	0.4728 (3)	0.8992 (2)	0.10094 (13)	0.1458 (8)	
Cl4	0.2257 (2)	0.93632 (14)	0.06158 (9)	0.1169 (6)	
C14	−0.7130 (7)	0.6657 (5)	0.3733 (3)	0.0917 (19)	0.65
H14A	−0.7622	0.7437	0.3543	0.110*	0.65
H14B	−0.7438	0.6236	0.3546	0.110*	0.65
Cl5	−0.7328 (5)	0.6391 (3)	0.45165 (15)	0.1329 (15)	0.65
Cl6	−0.5546 (6)	0.6307 (7)	0.3585 (4)	0.167 (3)	0.65
C14A	−0.7130 (7)	0.6657 (5)	0.3733 (3)	0.0917 (19)	0.35
H14C	−0.7144	0.6199	0.3465	0.110*	0.35
H14D	−0.7421	0.7406	0.3471	0.110*	0.35
Cl5A	−0.832 (3)	0.6662 (17)	0.4298 (7)	0.355 (14)	0.35
Cl6A	−0.5736 (14)	0.6287 (15)	0.3920 (10)	0.276 (12)	0.35
O6	−0.0892 (5)	−0.0527 (5)	0.3189 (3)	0.1181 (17)	
H6OA	−0.013 (3)	−0.063 (6)	0.316 (4)	0.13 (3)*	
H6OB	−0.133 (5)	0.012 (2)	0.323 (3)	0.078 (19)*	
O7	−1.0377 (12)	0.7546 (9)	0.4039 (6)	0.141 (4)	0.5

### (Bis{[(diphenylphosphanyl)methyl]diphenylphosphanylidene}(ethoxyoxoethanylidene)methane-κ4P,C,C',P')carbonyl(ethoxyoxoethanide)iridium(III) dichloride–methylene chloride–water (1/2/1.5) (7) Atomic displacement parameters (Å^2^)

**Table:**

	*U*^11^	*U*^22^	*U*^33^	*U*^12^	*U*^13^	*U*^23^
Ir1	0.02408 (6)	0.02543 (7)	0.02014 (6)	−0.01177 (5)	−0.00110 (4)	−0.00062 (4)
P1	0.0247 (4)	0.0330 (4)	0.0248 (4)	−0.0140 (3)	−0.0033 (3)	−0.0001 (3)
P2	0.0283 (4)	0.0362 (4)	0.0228 (4)	−0.0159 (3)	0.0004 (3)	−0.0008 (3)
P3	0.0255 (4)	0.0285 (4)	0.0210 (3)	−0.0125 (3)	−0.0025 (3)	−0.0003 (3)
P4	0.0235 (4)	0.0255 (4)	0.0236 (4)	−0.0111 (3)	0.0011 (3)	−0.0013 (3)
O1	0.0451 (14)	0.0380 (13)	0.0475 (14)	−0.0129 (11)	−0.0168 (12)	−0.0114 (11)
O2	0.0393 (13)	0.0257 (11)	0.0495 (14)	−0.0082 (10)	−0.0123 (11)	−0.0071 (10)
O3	0.0543 (17)	0.077 (2)	0.0410 (14)	−0.0398 (16)	0.0130 (13)	−0.0048 (13)
O4	0.0694 (19)	0.0627 (17)	0.0328 (13)	−0.0365 (15)	0.0086 (12)	−0.0165 (12)
O5	0.0646 (19)	0.0441 (15)	0.0625 (18)	−0.0250 (14)	0.0087 (14)	−0.0249 (14)
C1	0.0271 (15)	0.0310 (15)	0.0190 (13)	−0.0142 (13)	0.0007 (11)	−0.0018 (11)
C2	0.0299 (16)	0.0413 (18)	0.0273 (15)	−0.0217 (14)	−0.0020 (12)	0.0010 (13)
C3	0.0251 (15)	0.0302 (15)	0.0267 (15)	−0.0138 (12)	−0.0009 (12)	−0.0017 (12)
C4	0.0233 (15)	0.0288 (15)	0.0230 (14)	−0.0083 (12)	−0.0001 (11)	−0.0041 (12)
C5	0.0318 (17)	0.0286 (16)	0.0303 (16)	−0.0083 (13)	0.0024 (13)	−0.0066 (13)
C6	0.060 (3)	0.0301 (18)	0.067 (3)	−0.0143 (18)	−0.015 (2)	−0.0126 (18)
C7	0.098 (4)	0.032 (2)	0.134 (5)	−0.011 (2)	−0.055 (4)	−0.008 (3)
C8	0.0356 (17)	0.0345 (17)	0.0230 (15)	−0.0131 (14)	−0.0050 (13)	0.0037 (12)
C9	0.0389 (19)	0.0400 (18)	0.0230 (15)	−0.0151 (15)	−0.0038 (13)	0.0052 (13)
C10	0.086 (4)	0.071 (3)	0.039 (2)	−0.024 (3)	0.003 (2)	−0.022 (2)
C11	0.161 (7)	0.079 (4)	0.070 (4)	−0.053 (4)	0.006 (4)	−0.034 (3)
C12	0.0338 (17)	0.0349 (17)	0.0302 (16)	−0.0163 (14)	−0.0016 (13)	−0.0033 (14)
C101	0.0306 (16)	0.0398 (18)	0.0325 (16)	−0.0190 (14)	−0.0019 (13)	−0.0043 (14)
C102	0.062 (3)	0.045 (2)	0.052 (2)	−0.026 (2)	−0.0127 (19)	−0.0044 (18)
C103	0.085 (3)	0.051 (3)	0.075 (3)	−0.038 (2)	−0.009 (3)	−0.018 (2)
C104	0.070 (3)	0.069 (3)	0.056 (3)	−0.035 (2)	−0.013 (2)	−0.022 (2)
C105	0.051 (2)	0.065 (3)	0.041 (2)	−0.028 (2)	−0.0091 (17)	−0.0112 (18)
C106	0.042 (2)	0.045 (2)	0.0369 (18)	−0.0198 (17)	−0.0063 (15)	−0.0058 (15)
C107	0.0288 (16)	0.0409 (18)	0.0290 (16)	−0.0123 (14)	−0.0037 (13)	−0.0045 (13)
C108	0.0335 (18)	0.0423 (19)	0.0357 (18)	−0.0115 (15)	−0.0036 (14)	−0.0031 (15)
C109	0.048 (2)	0.044 (2)	0.059 (2)	−0.0092 (18)	−0.0060 (19)	−0.0071 (19)
C110	0.038 (2)	0.062 (3)	0.070 (3)	0.000 (2)	0.002 (2)	−0.018 (2)
C111	0.032 (2)	0.070 (3)	0.068 (3)	−0.017 (2)	0.0058 (19)	−0.016 (2)
C112	0.0328 (18)	0.055 (2)	0.047 (2)	−0.0175 (17)	0.0020 (15)	−0.0099 (17)
C201	0.0348 (17)	0.0460 (19)	0.0253 (15)	−0.0225 (15)	−0.0028 (13)	0.0055 (14)
C202	0.063 (3)	0.045 (2)	0.0362 (19)	−0.0252 (19)	−0.0037 (17)	0.0026 (16)
C203	0.103 (4)	0.043 (2)	0.056 (3)	−0.030 (3)	−0.008 (3)	0.010 (2)
C204	0.096 (4)	0.068 (3)	0.046 (3)	−0.038 (3)	−0.009 (2)	0.024 (2)
C205	0.093 (4)	0.084 (4)	0.026 (2)	−0.042 (3)	−0.002 (2)	0.008 (2)
C206	0.068 (3)	0.060 (3)	0.0281 (18)	−0.028 (2)	−0.0005 (17)	0.0002 (17)
C207	0.0394 (19)	0.047 (2)	0.0286 (16)	−0.0144 (16)	0.0083 (14)	−0.0062 (15)
C208	0.068 (3)	0.058 (3)	0.041 (2)	−0.029 (2)	0.0203 (19)	−0.0191 (19)
C209	0.114 (5)	0.075 (4)	0.065 (3)	−0.035 (3)	0.033 (3)	−0.041 (3)
C210	0.090 (5)	0.094 (5)	0.096 (5)	−0.004 (4)	0.031 (4)	−0.055 (4)
C211	0.050 (3)	0.133 (6)	0.085 (4)	0.001 (3)	0.020 (3)	−0.049 (4)
C212	0.040 (2)	0.087 (3)	0.048 (2)	−0.016 (2)	0.0090 (18)	−0.021 (2)
C301	0.0282 (16)	0.0372 (17)	0.0246 (15)	−0.0097 (13)	−0.0045 (12)	−0.0037 (13)
C302	0.052 (2)	0.051 (2)	0.0295 (17)	−0.0227 (18)	−0.0041 (15)	−0.0013 (16)
C303	0.080 (3)	0.070 (3)	0.0256 (18)	−0.028 (3)	−0.0077 (19)	0.0006 (18)
C304	0.070 (3)	0.077 (3)	0.030 (2)	−0.015 (2)	−0.0125 (19)	−0.016 (2)
C305	0.068 (3)	0.059 (3)	0.048 (2)	−0.020 (2)	−0.019 (2)	−0.018 (2)
C306	0.050 (2)	0.043 (2)	0.0374 (19)	−0.0162 (17)	−0.0116 (16)	−0.0079 (16)
C307	0.0285 (16)	0.0286 (15)	0.0273 (15)	−0.0121 (13)	0.0013 (12)	−0.0001 (12)
C308	0.0355 (18)	0.0368 (18)	0.0402 (18)	−0.0157 (15)	−0.0040 (14)	−0.0036 (15)
C309	0.058 (2)	0.040 (2)	0.056 (2)	−0.0215 (19)	−0.0046 (19)	−0.0138 (18)
C310	0.055 (2)	0.0330 (19)	0.058 (2)	−0.0088 (18)	0.0016 (19)	−0.0101 (17)
C311	0.0363 (19)	0.0384 (19)	0.047 (2)	−0.0077 (16)	−0.0011 (16)	−0.0004 (16)
C312	0.0350 (18)	0.0348 (17)	0.0353 (17)	−0.0149 (14)	−0.0019 (14)	−0.0014 (14)
C401	0.0338 (16)	0.0315 (16)	0.0240 (14)	−0.0170 (13)	−0.0024 (12)	0.0003 (12)
C402	0.043 (2)	0.0355 (18)	0.0407 (19)	−0.0199 (16)	0.0069 (15)	−0.0046 (15)
C403	0.056 (2)	0.0312 (18)	0.055 (2)	−0.0185 (17)	−0.0004 (19)	−0.0017 (16)
C404	0.061 (2)	0.045 (2)	0.043 (2)	−0.035 (2)	0.0030 (18)	0.0031 (16)
C405	0.050 (2)	0.054 (2)	0.045 (2)	−0.0336 (19)	0.0110 (17)	−0.0024 (17)
C406	0.0371 (18)	0.0406 (19)	0.0423 (19)	−0.0222 (16)	0.0083 (15)	−0.0041 (15)
C407	0.0310 (16)	0.0291 (16)	0.0339 (16)	−0.0144 (13)	0.0082 (13)	−0.0047 (13)
C408	0.040 (2)	0.051 (2)	0.048 (2)	−0.0175 (17)	0.0072 (16)	−0.0195 (18)
C409	0.065 (3)	0.064 (3)	0.062 (3)	−0.032 (2)	0.021 (2)	−0.035 (2)
C410	0.063 (3)	0.044 (2)	0.075 (3)	−0.007 (2)	0.021 (2)	−0.025 (2)
C411	0.054 (3)	0.048 (2)	0.064 (3)	0.004 (2)	0.011 (2)	−0.006 (2)
C412	0.038 (2)	0.045 (2)	0.041 (2)	−0.0052 (16)	0.0055 (16)	−0.0030 (16)
Cl1	0.0438 (5)	0.0810 (7)	0.0495 (5)	−0.0391 (5)	−0.0037 (4)	0.0043 (5)
Cl2	0.0611 (6)	0.0621 (6)	0.0573 (6)	−0.0275 (5)	−0.0059 (5)	−0.0071 (5)
C13	0.154 (6)	0.068 (3)	0.083 (4)	−0.052 (4)	−0.001 (4)	−0.031 (3)
Cl3	0.153 (2)	0.1225 (17)	0.162 (2)	−0.0639 (16)	0.0071 (17)	−0.0252 (15)
Cl4	0.1430 (17)	0.0734 (10)	0.1140 (14)	−0.0276 (10)	0.0145 (12)	−0.0223 (9)
C14	0.123 (5)	0.071 (4)	0.082 (4)	−0.041 (4)	−0.031 (4)	−0.012 (3)
Cl5	0.222 (5)	0.0797 (17)	0.0748 (18)	−0.050 (2)	0.002 (2)	−0.0040 (14)
Cl6	0.092 (3)	0.148 (4)	0.213 (6)	−0.023 (3)	0.027 (3)	−0.016 (4)
C14A	0.123 (5)	0.071 (4)	0.082 (4)	−0.041 (4)	−0.031 (4)	−0.012 (3)
Cl5A	0.63 (4)	0.40 (2)	0.173 (13)	−0.38 (3)	−0.003 (18)	−0.010 (14)
Cl6A	0.169 (13)	0.207 (13)	0.47 (3)	−0.030 (9)	−0.151 (16)	−0.171 (18)
O6	0.084 (4)	0.102 (4)	0.173 (5)	−0.049 (3)	0.035 (4)	−0.031 (4)
O7	0.137 (10)	0.108 (8)	0.188 (12)	−0.055 (7)	0.021 (9)	−0.049 (8)

### (Bis{[(diphenylphosphanyl)methyl]diphenylphosphanylidene}(ethoxyoxoethanylidene)methane-κ4P,C,C',P')carbonyl(ethoxyoxoethanide)iridium(III) dichloride–methylene chloride–water (1/2/1.5) (7) Geometric parameters (Å, º)

**Table:**

Ir1—C12	1.910 (3)	C203—H203	0.9400
Ir1—C4	2.119 (3)	C204—C205	1.361 (7)
Ir1—C8	2.177 (3)	C204—H204	0.9400
Ir1—C1	2.225 (3)	C205—C206	1.385 (6)
Ir1—P1	2.3393 (8)	C205—H205	0.9400
Ir1—P4	2.3658 (8)	C206—H206	0.9400
P1—C101	1.811 (3)	C207—C208	1.382 (6)
P1—C107	1.822 (3)	C207—C212	1.385 (6)
P1—C2	1.839 (3)	C208—C209	1.381 (6)
P2—C201	1.800 (3)	C208—H208	0.9400
P2—C207	1.802 (4)	C209—C210	1.353 (9)
P2—C2	1.810 (3)	C209—H209	0.9400
P2—C1	1.837 (3)	C210—C211	1.380 (10)
P3—C1	1.791 (3)	C210—H210	0.9400
P3—C3	1.798 (3)	C211—C212	1.398 (7)
P3—C307	1.804 (3)	C211—H211	0.9400
P3—C301	1.808 (3)	C212—H212	0.9400
P4—C407	1.820 (3)	C301—C306	1.387 (5)
P4—C401	1.821 (3)	C301—C302	1.390 (5)
P4—C3	1.840 (3)	C302—C303	1.381 (5)
O1—C5	1.208 (4)	C302—H302	0.9400
O2—C5	1.340 (4)	C303—C304	1.364 (7)
O2—C6	1.468 (4)	C303—H303	0.9400
O3—C9	1.205 (4)	C304—C305	1.374 (7)
O4—C9	1.348 (4)	C304—H304	0.9400
O4—C10	1.446 (5)	C305—C306	1.393 (5)
O5—C12	1.120 (4)	C305—H305	0.9400
C1—C4	1.515 (4)	C306—H306	0.9400
C2—H2A	0.9800	C307—C308	1.392 (5)
C2—H2B	0.9800	C307—C312	1.398 (4)
C3—H3A	0.9800	C308—C309	1.388 (5)
C3—H3B	0.9800	C308—H308	0.9400
C4—C5	1.488 (4)	C309—C310	1.376 (6)
C4—H4	0.942 (18)	C309—H309	0.9400
C6—C7	1.479 (6)	C310—C311	1.381 (6)
C6—H6A	0.9800	C310—H310	0.9400
C6—H6B	0.9800	C311—C312	1.384 (5)
C7—H7A	0.9700	C311—H311	0.9400
C7—H7B	0.9700	C312—H312	0.9400
C7—H7C	0.9700	C401—C402	1.385 (5)
C8—C9	1.470 (5)	C401—C406	1.386 (5)
C8—H8A	0.9800	C402—C403	1.386 (5)
C8—H8B	0.9800	C402—H402	0.9400
C10—C11	1.485 (8)	C403—C404	1.371 (6)
C10—H10A	0.9800	C403—H403	0.9400
C10—H10B	0.9800	C404—C405	1.371 (6)
C11—H11A	0.9700	C404—H404	0.9400
C11—H11B	0.9700	C405—C406	1.385 (5)
C11—H11C	0.9700	C405—H405	0.9400
C101—C102	1.388 (5)	C406—H406	0.9400
C101—C106	1.394 (5)	C407—C408	1.385 (5)
C102—C103	1.389 (6)	C407—C412	1.395 (5)
C102—H102	0.9400	C408—C409	1.383 (5)
C103—C104	1.363 (6)	C408—H408	0.9400
C103—H103	0.9400	C409—C410	1.373 (7)
C104—C105	1.359 (6)	C409—H409	0.9400
C104—H104	0.9400	C410—C411	1.359 (7)
C105—C106	1.384 (5)	C410—H410	0.9400
C105—H105	0.9400	C411—C412	1.380 (6)
C106—H106	0.9400	C411—H411	0.9400
C107—C108	1.389 (5)	C412—H412	0.9400
C107—C112	1.398 (5)	C13—Cl3	1.736 (8)
C108—C109	1.383 (5)	C13—Cl4	1.746 (7)
C108—H108	0.9400	C13—H13A	0.9800
C109—C110	1.386 (7)	C13—H13B	0.9800
C109—H109	0.9400	C14—Cl5	1.713 (7)
C110—C111	1.385 (7)	C14—Cl6	1.745 (10)
C110—H110	0.9400	C14—H14A	0.9800
C111—C112	1.370 (6)	C14—H14B	0.9800
C111—H111	0.9400	C14A—Cl6A	1.545 (15)
C112—H112	0.9400	C14A—Cl5A	1.82 (2)
C201—C202	1.381 (5)	C14A—H14C	0.9800
C201—C206	1.403 (5)	C14A—H14D	0.9800
C202—C203	1.380 (5)	O6—H6OA	0.85 (2)
C202—H202	0.9400	O6—H6OB	0.845 (19)
C203—C204	1.377 (7)		
			
C12—Ir1—C4	158.78 (12)	C112—C111—H111	119.9
C12—Ir1—C8	93.36 (13)	C110—C111—H111	119.9
C4—Ir1—C8	106.70 (12)	C111—C112—C107	120.2 (4)
C12—Ir1—C1	118.82 (12)	C111—C112—H112	119.9
C4—Ir1—C1	40.72 (11)	C107—C112—H112	119.9
C8—Ir1—C1	147.39 (12)	C202—C201—C206	119.1 (3)
C12—Ir1—P1	88.65 (10)	C202—C201—P2	121.8 (3)
C4—Ir1—P1	85.11 (9)	C206—C201—P2	118.9 (3)
C8—Ir1—P1	88.50 (9)	C203—C202—C201	120.2 (4)
C1—Ir1—P1	87.22 (8)	C203—C202—H202	119.9
C12—Ir1—P4	91.76 (10)	C201—C202—H202	119.9
C4—Ir1—P4	93.24 (8)	C204—C203—C202	120.5 (5)
C8—Ir1—P4	95.09 (9)	C204—C203—H203	119.7
C1—Ir1—P4	89.41 (8)	C202—C203—H203	119.7
P1—Ir1—P4	176.35 (3)	C205—C204—C203	119.9 (4)
C101—P1—C107	102.78 (15)	C205—C204—H204	120.1
C101—P1—C2	106.38 (15)	C203—C204—H204	120.1
C107—P1—C2	105.77 (15)	C204—C205—C206	120.9 (4)
C101—P1—Ir1	116.72 (11)	C204—C205—H205	119.6
C107—P1—Ir1	120.29 (11)	C206—C205—H205	119.6
C2—P1—Ir1	103.76 (10)	C205—C206—C201	119.4 (4)
C201—P2—C207	105.53 (16)	C205—C206—H206	120.3
C201—P2—C2	107.18 (15)	C201—C206—H206	120.3
C207—P2—C2	110.99 (17)	C208—C207—C212	120.6 (4)
C201—P2—C1	120.40 (15)	C208—C207—P2	119.7 (3)
C207—P2—C1	107.20 (16)	C212—C207—P2	119.7 (3)
C2—P2—C1	105.50 (14)	C209—C208—C207	120.2 (5)
C1—P3—C3	106.12 (14)	C209—C208—H208	119.9
C1—P3—C307	111.84 (14)	C207—C208—H208	119.9
C3—P3—C307	107.34 (14)	C210—C209—C208	120.1 (6)
C1—P3—C301	117.32 (14)	C210—C209—H209	120.0
C3—P3—C301	108.18 (15)	C208—C209—H209	120.0
C307—P3—C301	105.63 (15)	C209—C210—C211	120.3 (5)
C407—P4—C401	104.51 (14)	C209—C210—H210	119.9
C407—P4—C3	105.94 (15)	C211—C210—H210	119.9
C401—P4—C3	103.41 (14)	C210—C211—C212	121.1 (5)
C407—P4—Ir1	116.18 (11)	C210—C211—H211	119.5
C401—P4—Ir1	119.97 (11)	C212—C211—H211	119.5
C3—P4—Ir1	105.34 (10)	C207—C212—C211	117.7 (5)
C5—O2—C6	116.8 (3)	C207—C212—H212	121.1
C9—O4—C10	116.1 (3)	C211—C212—H212	121.1
C4—C1—P3	121.7 (2)	C306—C301—C302	119.5 (3)
C4—C1—P2	108.3 (2)	C306—C301—P3	122.3 (3)
P3—C1—P2	122.16 (16)	C302—C301—P3	118.2 (3)
C4—C1—Ir1	65.88 (15)	C303—C302—C301	119.8 (4)
P3—C1—Ir1	110.27 (14)	C303—C302—H302	120.1
P2—C1—Ir1	115.59 (14)	C301—C302—H302	120.1
P2—C2—P1	111.79 (17)	C304—C303—C302	120.5 (4)
P2—C2—H2A	109.3	C304—C303—H303	119.8
P1—C2—H2A	109.3	C302—C303—H303	119.8
P2—C2—H2B	109.3	C303—C304—C305	120.7 (4)
P1—C2—H2B	109.3	C303—C304—H304	119.6
H2A—C2—H2B	107.9	C305—C304—H304	119.6
P3—C3—P4	110.67 (16)	C304—C305—C306	119.6 (4)
P3—C3—H3A	109.5	C304—C305—H305	120.2
P4—C3—H3A	109.5	C306—C305—H305	120.2
P3—C3—H3B	109.5	C301—C306—C305	119.9 (4)
P4—C3—H3B	109.5	C301—C306—H306	120.1
H3A—C3—H3B	108.1	C305—C306—H306	120.1
C5—C4—C1	125.7 (3)	C308—C307—C312	119.2 (3)
C5—C4—Ir1	131.9 (2)	C308—C307—P3	124.0 (2)
C1—C4—Ir1	73.40 (16)	C312—C307—P3	116.5 (2)
C5—C4—H4	104 (2)	C309—C308—C307	119.9 (3)
C1—C4—H4	112 (2)	C309—C308—H308	120.1
Ir1—C4—H4	108 (2)	C307—C308—H308	120.1
O1—C5—O2	122.8 (3)	C310—C309—C308	120.4 (4)
O1—C5—C4	127.9 (3)	C310—C309—H309	119.8
O2—C5—C4	109.2 (3)	C308—C309—H309	119.8
O2—C6—C7	106.6 (3)	C309—C310—C311	120.3 (4)
O2—C6—H6A	110.4	C309—C310—H310	119.8
C7—C6—H6A	110.4	C311—C310—H310	119.8
O2—C6—H6B	110.4	C310—C311—C312	119.9 (3)
C7—C6—H6B	110.4	C310—C311—H311	120.0
H6A—C6—H6B	108.6	C312—C311—H311	120.0
C6—C7—H7A	109.5	C311—C312—C307	120.2 (3)
C6—C7—H7B	109.5	C311—C312—H312	119.9
H7A—C7—H7B	109.5	C307—C312—H312	119.9
C6—C7—H7C	109.5	C402—C401—C406	119.1 (3)
H7A—C7—H7C	109.5	C402—C401—P4	119.2 (2)
H7B—C7—H7C	109.5	C406—C401—P4	121.7 (3)
C9—C8—Ir1	114.1 (2)	C401—C402—C403	120.0 (3)
C9—C8—H8A	108.7	C401—C402—H402	120.0
Ir1—C8—H8A	108.7	C403—C402—H402	120.0
C9—C8—H8B	108.7	C404—C403—C402	120.4 (4)
Ir1—C8—H8B	108.7	C404—C403—H403	119.8
H8A—C8—H8B	107.6	C402—C403—H403	119.8
O3—C9—O4	122.3 (3)	C405—C404—C403	120.0 (3)
O3—C9—C8	126.2 (3)	C405—C404—H404	120.0
O4—C9—C8	111.5 (3)	C403—C404—H404	120.0
O4—C10—C11	107.2 (5)	C404—C405—C406	120.2 (4)
O4—C10—H10A	110.3	C404—C405—H405	119.9
C11—C10—H10A	110.3	C406—C405—H405	119.9
O4—C10—H10B	110.3	C405—C406—C401	120.3 (3)
C11—C10—H10B	110.3	C405—C406—H406	119.9
H10A—C10—H10B	108.5	C401—C406—H406	119.9
C10—C11—H11A	109.5	C408—C407—C412	118.9 (3)
C10—C11—H11B	109.5	C408—C407—P4	118.6 (3)
H11A—C11—H11B	109.5	C412—C407—P4	122.6 (3)
C10—C11—H11C	109.5	C409—C408—C407	120.4 (4)
H11A—C11—H11C	109.5	C409—C408—H408	119.8
H11B—C11—H11C	109.5	C407—C408—H408	119.8
O5—C12—Ir1	177.9 (3)	C410—C409—C408	119.7 (4)
C102—C101—C106	119.1 (3)	C410—C409—H409	120.2
C102—C101—P1	122.4 (3)	C408—C409—H409	120.2
C106—C101—P1	118.2 (3)	C411—C410—C409	120.6 (4)
C101—C102—C103	119.3 (4)	C411—C410—H410	119.7
C101—C102—H102	120.4	C409—C410—H410	119.7
C103—C102—H102	120.4	C410—C411—C412	120.5 (4)
C104—C103—C102	120.7 (4)	C410—C411—H411	119.7
C104—C103—H103	119.6	C412—C411—H411	119.7
C102—C103—H103	119.6	C411—C412—C407	119.8 (4)
C105—C104—C103	120.7 (4)	C411—C412—H412	120.1
C105—C104—H104	119.7	C407—C412—H412	120.1
C103—C104—H104	119.7	Cl3—C13—Cl4	111.3 (4)
C104—C105—C106	120.0 (4)	Cl3—C13—H13A	109.4
C104—C105—H105	120.0	Cl4—C13—H13A	109.4
C106—C105—H105	120.0	Cl3—C13—H13B	109.4
C105—C106—C101	120.2 (4)	Cl4—C13—H13B	109.4
C105—C106—H106	119.9	H13A—C13—H13B	108.0
C101—C106—H106	119.9	Cl5—C14—Cl6	110.5 (4)
C108—C107—C112	119.1 (3)	Cl5—C14—H14A	109.6
C108—C107—P1	123.4 (3)	Cl6—C14—H14A	109.6
C112—C107—P1	117.5 (3)	Cl5—C14—H14B	109.6
C109—C108—C107	120.7 (4)	Cl6—C14—H14B	109.6
C109—C108—H108	119.7	H14A—C14—H14B	108.1
C107—C108—H108	119.7	Cl6A—C14A—Cl5A	123.0 (11)
C108—C109—C110	119.4 (4)	Cl6A—C14A—H14C	106.6
C108—C109—H109	120.3	Cl5A—C14A—H14C	106.6
C110—C109—H109	120.3	Cl6A—C14A—H14D	106.6
C111—C110—C109	120.3 (4)	Cl5A—C14A—H14D	106.6
C111—C110—H110	119.9	H14C—C14A—H14D	106.5
C109—C110—H110	119.9	H6OA—O6—H6OB	109 (7)
C112—C111—C110	120.3 (4)		
			
C3—P3—C1—C4	−32.3 (3)	C1—P2—C201—C206	101.9 (3)
C307—P3—C1—C4	−149.1 (2)	C206—C201—C202—C203	−1.9 (6)
C301—P3—C1—C4	88.7 (3)	P2—C201—C202—C203	−176.6 (4)
C3—P3—C1—P2	−177.92 (18)	C201—C202—C203—C204	1.0 (8)
C307—P3—C1—P2	65.3 (2)	C202—C203—C204—C205	0.1 (9)
C301—P3—C1—P2	−56.9 (2)	C203—C204—C205—C206	−0.4 (9)
C3—P3—C1—Ir1	41.19 (18)	C204—C205—C206—C201	−0.5 (8)
C307—P3—C1—Ir1	−75.56 (17)	C202—C201—C206—C205	1.6 (6)
C301—P3—C1—Ir1	162.17 (14)	P2—C201—C206—C205	176.5 (4)
C201—P2—C1—C4	−159.6 (2)	C201—P2—C207—C208	91.7 (3)
C207—P2—C1—C4	−39.2 (2)	C2—P2—C207—C208	−152.5 (3)
C2—P2—C1—C4	79.2 (2)	C1—P2—C207—C208	−37.7 (3)
C201—P2—C1—P3	−10.0 (3)	C201—P2—C207—C212	−86.1 (3)
C207—P2—C1—P3	110.4 (2)	C2—P2—C207—C212	29.7 (4)
C2—P2—C1—P3	−131.2 (2)	C1—P2—C207—C212	144.4 (3)
C201—P2—C1—Ir1	128.96 (17)	C212—C207—C208—C209	−1.7 (6)
C207—P2—C1—Ir1	−110.60 (17)	P2—C207—C208—C209	−179.5 (4)
C2—P2—C1—Ir1	7.8 (2)	C207—C208—C209—C210	0.1 (8)
C201—P2—C2—P1	−163.12 (18)	C208—C209—C210—C211	1.4 (10)
C207—P2—C2—P1	82.1 (2)	C209—C210—C211—C212	−1.4 (11)
C1—P2—C2—P1	−33.7 (2)	C208—C207—C212—C211	1.6 (6)
C101—P1—C2—P2	167.17 (18)	P2—C207—C212—C211	179.4 (4)
C107—P1—C2—P2	−84.0 (2)	C210—C211—C212—C207	−0.1 (9)
Ir1—P1—C2—P2	43.49 (19)	C1—P3—C301—C306	−96.7 (3)
C1—P3—C3—P4	−45.4 (2)	C3—P3—C301—C306	23.2 (3)
C307—P3—C3—P4	74.32 (19)	C307—P3—C301—C306	137.9 (3)
C301—P3—C3—P4	−172.12 (15)	C1—P3—C301—C302	85.3 (3)
C407—P4—C3—P3	−94.87 (18)	C3—P3—C301—C302	−154.8 (3)
C401—P4—C3—P3	155.50 (17)	C307—P3—C301—C302	−40.1 (3)
Ir1—P4—C3—P3	28.78 (17)	C306—C301—C302—C303	1.6 (6)
P3—C1—C4—C5	−30.3 (4)	P3—C301—C302—C303	179.7 (3)
P2—C1—C4—C5	119.4 (3)	C301—C302—C303—C304	−1.3 (7)
Ir1—C1—C4—C5	−130.1 (3)	C302—C303—C304—C305	0.2 (8)
P3—C1—C4—Ir1	99.8 (2)	C303—C304—C305—C306	0.4 (7)
P2—C1—C4—Ir1	−110.49 (16)	C302—C301—C306—C305	−1.0 (6)
C6—O2—C5—O1	−0.8 (5)	P3—C301—C306—C305	−179.0 (3)
C6—O2—C5—C4	177.3 (3)	C304—C305—C306—C301	0.0 (6)
C1—C4—C5—O1	2.1 (5)	C1—P3—C307—C308	10.8 (3)
Ir1—C4—C5—O1	−97.9 (4)	C3—P3—C307—C308	−105.2 (3)
C1—C4—C5—O2	−175.9 (3)	C301—P3—C307—C308	139.5 (3)
Ir1—C4—C5—O2	84.2 (3)	C1—P3—C307—C312	−174.7 (2)
C5—O2—C6—C7	171.6 (4)	C3—P3—C307—C312	69.3 (3)
C10—O4—C9—O3	2.9 (5)	C301—P3—C307—C312	−46.0 (3)
C10—O4—C9—C8	−175.7 (3)	C312—C307—C308—C309	0.6 (5)
Ir1—C8—C9—O3	−95.1 (4)	P3—C307—C308—C309	175.0 (3)
Ir1—C8—C9—O4	83.4 (3)	C307—C308—C309—C310	−1.7 (6)
C9—O4—C10—C11	−178.5 (4)	C308—C309—C310—C311	1.1 (6)
C107—P1—C101—C102	−143.3 (3)	C309—C310—C311—C312	0.5 (6)
C2—P1—C101—C102	−32.4 (4)	C310—C311—C312—C307	−1.6 (5)
Ir1—P1—C101—C102	82.8 (3)	C308—C307—C312—C311	1.0 (5)
C107—P1—C101—C106	42.1 (3)	P3—C307—C312—C311	−173.8 (3)
C2—P1—C101—C106	153.0 (3)	C407—P4—C401—C402	166.4 (3)
Ir1—P1—C101—C106	−91.8 (3)	C3—P4—C401—C402	−82.9 (3)
C106—C101—C102—C103	−1.9 (6)	Ir1—P4—C401—C402	34.0 (3)
P1—C101—C102—C103	−176.5 (4)	C407—P4—C401—C406	−12.3 (3)
C101—C102—C103—C104	1.2 (7)	C3—P4—C401—C406	98.4 (3)
C102—C103—C104—C105	−0.1 (8)	Ir1—P4—C401—C406	−144.8 (2)
C103—C104—C105—C106	−0.1 (7)	C406—C401—C402—C403	−0.6 (5)
C104—C105—C106—C101	−0.6 (6)	P4—C401—C402—C403	−179.4 (3)
C102—C101—C106—C105	1.6 (6)	C401—C402—C403—C404	0.3 (6)
P1—C101—C106—C105	176.4 (3)	C402—C403—C404—C405	0.2 (6)
C101—P1—C107—C108	−121.0 (3)	C403—C404—C405—C406	−0.4 (6)
C2—P1—C107—C108	127.6 (3)	C404—C405—C406—C401	0.1 (6)
Ir1—P1—C107—C108	10.8 (3)	C402—C401—C406—C405	0.4 (5)
C101—P1—C107—C112	57.8 (3)	P4—C401—C406—C405	179.1 (3)
C2—P1—C107—C112	−53.6 (3)	C401—P4—C407—C408	−73.7 (3)
Ir1—P1—C107—C112	−170.4 (2)	C3—P4—C407—C408	177.4 (3)
C112—C107—C108—C109	1.7 (5)	Ir1—P4—C407—C408	60.9 (3)
P1—C107—C108—C109	−179.5 (3)	C401—P4—C407—C412	105.6 (3)
C107—C108—C109—C110	−0.5 (6)	C3—P4—C407—C412	−3.3 (3)
C108—C109—C110—C111	−0.6 (7)	Ir1—P4—C407—C412	−119.8 (3)
C109—C110—C111—C112	0.5 (7)	C412—C407—C408—C409	2.0 (6)
C110—C111—C112—C107	0.8 (6)	P4—C407—C408—C409	−178.7 (3)
C108—C107—C112—C111	−1.8 (5)	C407—C408—C409—C410	0.4 (7)
P1—C107—C112—C111	179.3 (3)	C408—C409—C410—C411	−2.1 (7)
C207—P2—C201—C202	155.4 (3)	C409—C410—C411—C412	1.3 (8)
C2—P2—C201—C202	37.0 (3)	C410—C411—C412—C407	1.2 (7)
C1—P2—C201—C202	−83.3 (3)	C408—C407—C412—C411	−2.8 (6)
C207—P2—C201—C206	−19.4 (3)	P4—C407—C412—C411	177.9 (3)
C2—P2—C201—C206	−137.7 (3)		

### (Bis{[(diphenylphosphanyl)methyl]diphenylphosphanylidene}(ethoxyoxoethanylidene)methane-κ4P,C,C',P')carbonyl(ethoxyoxoethanide)iridium(III) dichloride–methylene chloride–water (1/2/1.5) (7) Hydrogen-bond geometry (Å, º)

**Table:**

*D*—H···*A*	*D*—H	H···*A*	*D*···*A*	*D*—H···*A*
C2—H2*A*···Cl1	0.98	2.48	3.421 (3)	162
C3—H3*A*···Cl1^i^	0.98	2.59	3.488 (3)	152
C3—H3*B*···O1	0.98	2.21	2.968 (4)	134
C102—H102···Cl2	0.94	2.61	3.505 (4)	160
C108—H108···O2	0.94	2.61	3.313 (4)	132
C112—H112···Cl1	0.94	2.80	3.595 (4)	143
C202—H202···Cl2	0.94	2.70	3.574 (4)	156
C212—H212···Cl1	0.94	2.80	3.733 (5)	173
C306—H306···O1	0.94	2.47	3.061 (4)	121
C312—H312···Cl1^i^	0.94	2.73	3.503 (4)	140
C402—H402···O2	0.94	2.47	3.375 (4)	162
C408—H408···O3	0.94	2.44	3.326 (5)	156
C412—H412···Cl1^i^	0.94	2.97	3.866 (4)	161
C13—H13*A*···O5^ii^	0.98	2.58	3.194 (6)	121
C13—H13*A*···Cl2^ii^	0.98	2.68	3.500 (7)	141
C14—H14*A*···Cl2^iii^	0.98	2.65	3.553 (6)	153
C14—H14*B*···O1^iv^	0.98	2.37	3.327 (6)	164
C14*A*—H14*C*···O1^iv^	0.98	2.38	3.327 (6)	163
C14*A*—H14*D*···Cl2^iii^	0.98	2.59	3.553 (6)	168
O6—H6*OA*···Cl2	0.85 (2)	2.39 (4)	3.178 (5)	154 (7)
O6—H6*OB*···Cl1	0.85 (2)	2.39 (2)	3.239 (6)	178 (6)

Symmetry codes: (i) *x*+1, *y*, *z*; (ii) *x*, *y*+1, *z*; (iii) *x*−1, *y*+1, *z*; (iv) *x*−1, *y*, *z*.
